# Amidine- and Amidoxime-Substituted Heterocycles: Synthesis, Antiproliferative Evaluations and DNA Binding

**DOI:** 10.3390/molecules26227060

**Published:** 2021-11-22

**Authors:** Silvija Maračić, Petra Grbčić, Suresh Shammugam, Marijana Radić Stojković, Krešimir Pavelić, Mirela Sedić, Sandra Kraljević Pavelić, Silvana Raić-Malić

**Affiliations:** 1 Department of Organic Chemistry, Faculty of Chemical Engineering and Technology, University of Zagreb, Marulićev trg 19, HR-10000 Zagreb, Croatia; skorunda@fkit.hr; 2 Department of Biotechnology, University of Rijeka, Ulica Radmile Matejčić 2, HR-51000 Rijeka, Croatia; petra.grbcic@biotech.uniri.hr; 3 Division of Organic Chemistry and Biochemistry, Laboratory for Biomolecular Interactions and Spectroscopy, Ruđer Bošković Institute, Bijenička 54, HR-10000 Zagreb, Croatia; sshanmug@irb.hr; 4 Faculty of Medicine, Juraj Dobrila University of Pula, HR-52100 Pula, Croatia; pavelic@unipu.hr; 5 Centre for Applied Bioanthropology, Institute for Anthropological Research, Ljudevita Gaja 32, HR-10000 Zagreb, Croatia; mirela.sedic@inantro.hr; 6 Faculty of Health Studies, University of Rijeka, Ulica Viktora Cara Emina 5, HR-51000 Rijeka, Croatia; sandrakp@uniri.hr

**Keywords:** amidine, amidoxime, antiproliferative activity, DNA binding, fluorescence, CD spectroscopy, thermal denaturation

## Abstract

The novel 1,2,3-triazolyl-appended *N*- and *O*-heterocycles containing amidine **4**–**11** and amidoxime **12**–**22** moiety were prepared and evaluated for their antiproliferative activities in vitro. Among the series of amidine-substituted heterocycles, aromatic diamidine **5** and coumarine amidine **11** had the most potent growth-inhibitory effect on cervical carcinoma (HeLa), hepatocellular carcinoma (HepG2) and colorectal adenocarcinoma (SW620), with IC_50_ values in the nM range. Although compound **5** was toxic to non-tumor HFF cells, compound **11** showed certain selectivity. From the amidoxime series, quinoline amidoximes **18** and **20** showed antiproliferative effects on lung adenocarcinoma (A549), HeLa and SW620 cells emphasizing compound **20** that exhibited no cytostatic effect on normal HFF fibroblasts. Results of CD titrations and thermal melting experiments indicated that compounds **5** and **10** most likely bind inside the minor groove of AT-DNA and intercalate into AU-RNA. Compounds **6**, **9** and **11** bind to AT-DNA with mixed binding mode, most probably minor groove binding accompanied with aggregate binding along the DNA backbone.

## 1. Introduction

Nitrogen- and oxygen-containing heterocyclic compounds may be found in pharmaceuticals, natural products, dyes, organic materials, and particularly in biologically active molecules [1,2]. Natural and synthetic *N*- and *O*-heterocycles have been discovered as promising anti-cancer agents used in clinic or clinical evaluations, suggesting their prominent place in anti-cancer drugs development. Although the design of bioactive molecules is primarily focused on novel *N*-heterocycles, due to their ease of synthesis and possible mimicking of physiological molecules, *O*-heterocycles have provided equally potent molecules with a lesser risk of toxicity [2,3]. These compounds were found to act via various targets such as protein kinases, histone deacetylases, topoisomerase I and II, carbonic anhydrase, aromatase, sulfatase, vascular endothelial growth factor (VEGF), and epidermal growth factor (EGF) receptors, monocarboxylate transporters, DNA polynucleotide, tubulin/microtubule system [1,4,5,6,7,8,9,10,11,12,13,14]. 

**Figure 1:**
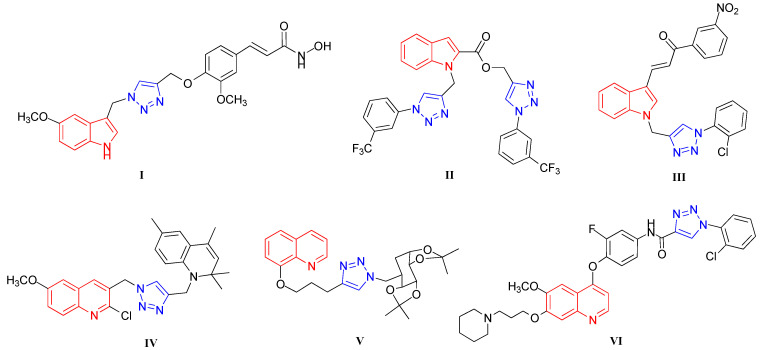
Triazolyl-appended *N*-heterocycles indole and quinoline with anticancer activities.

Applying the molecular hybridization approach for the synthesis of new molecules where 1,2,3-triazole ring acts as pharmacophore or linker leads to libraries of compounds with significant anticancer activity [15]. Thus, indole scaffold, found to be widely distributed in natural products and bioactive molecules, was linked to 1,2,3-triazole and yielded new hybrids with potent anticancer activity [11]. For example, in order to obtain histone deacetylases (HDAC) inhibitors with lower side effects, indole-derived N-hydroxyarylamide **I** with the triazole ring as a linker was obtained and showed the positive effects of 5-methoxy group and the one-carbon-bridge between triazole and the indole ring on antiproliferative activity (Figure 1) [16].

Bis-1,2,3-triazole–indole hybrids were further synthesized and evaluated for their in vitro and in vivo anticancer activity, among which compound **II** caused DNA cleavage and displayed significant activity against breast cancer (MCF-7), cervical carcinoma (HeLa) and embryonic kidney (HEK 293) cell lines, as compared with cisplatin [17]. Indole–1,2,3-triazole–chalcone **III** acted via noncovalent intercalative mode of binding to DNA, causing antiproliferative activity on human cervical cancer (SiHa) and colorectal epithelial carcinoma (SW620) cancer cell lines with no cytotoxicity in human embryonic kidney cells (HEK293) at the similar concentration [18]. Another triazolyl-appended *N*-heterocycles that exhibited anticancer activities are quinoline-based 1,2,3-triazole hybrids. New quinoline–1,2,3-triazole–dihydroquinoline derivatives were synthesized [19] and displayed cytotoxicity against lung adenocarcinoma (A549) and breast cancer (MCF7) cells, emphasizing the compound **IV** (Figure 1), that showed antiproliferative activity against A549 cells comparable to that of the reference drug doxorubicin. 8-Hydroxyquinoline derivative **V** with sugar moiety linked via 1,2,3-triazole ring exhibited potent antiproliferative activity and high selectivity toward ovarian cancer (OVCAR-03) cells, with higher activity than the doxorubicin [20]. The 1,2,3-triazole-4-carboxamide **VI** was identified as a multitargeted receptor tyrosine kinase inhibitor with a more potent growth-inhibition effect on human colon adenocarcinoma (HT-29) cells than foretinib [21].

**Figure 2:**
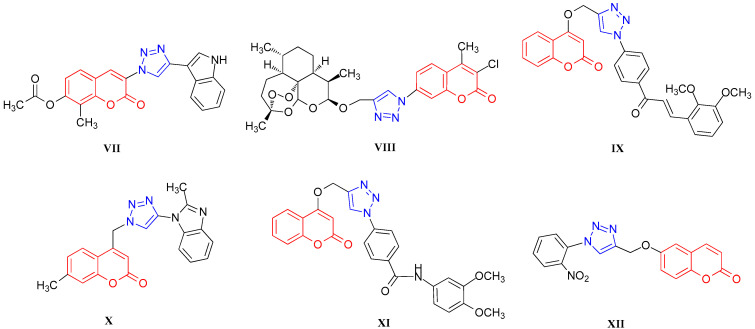
Coumarin-based 1,2,3-triazole hybrids with anticancer activities.

Hybridization of coumarin moiety with other anticancer pharmacophores was found to provide novel anticancer candidates with low toxicity, high specificity and excellent efficacy against drug-susceptible and drug-resistant cancers [3,13,22]. It was observed that substituent, length and position of alkyl spacer had a profound effect on the anticancer potency. 1,2,3-Triazole-containing novobiocin analogues with triazole ring at C-3 of coumarin were investigated [23] and showed that coumarin–1,2,3-triazole–indole **VII** (Figure 2) exhibited potent antiproliferative activity against two breast cancer cell lines (SKBr-3 and MCF-7), which was directly related to inhibition of the Hsp90-mediated protein folding process. Artemisinin–coumarin compound **VIII** accumulated in cancer-cell mitochondria efficiently and induced the generation of intracellular ROS, triggered cancer cell apoptosis, and consequently caused cancer cell death [24]. In addition, chalcone–coumarin hybrids displayed cytotoxicity against lymphoblastic leukemia (MOLT-3) cells without affecting normal cells [25]. Among these, compound **IX** showed strong activity against human liver cancer (HepG2) cells and no toxicity to Vero cells. Further investigations revealed that coumarin–1,2,3-triazole–benzimidazole derivative **X** induced cell death, mainly due to early apoptosis, and its inhibition of 5-lipoxygenase activity and perturbation of sphingolipid signaling by interfering with intracellular acid ceramidase activity [26]. Another coumarin–1,2,3-triazole hybrid **XI** containing amide showed moderate to excellent activity against human breast cancer (MDA-MB-231) cells, revealing general enhancement of proliferation-inhibiting activity under hypoxia, contrasted with normoxia [27]. 6-Hydroxycoumarin **XII** linked through triazole with ortho-substituted phenyl moiety showed to be selectively cytotoxic against lung cancer (A-549) cell line [28].

Heterocyclic diamidines have been extensively studied as DNA minor groove binders [29]. The research on amidines has been successful in demonstrating a correlation between the structure and biological activity with their ability to bind in minor grooves [29,30].

To improve bioavailability and oral absorption of amidines, the strategy of amidoxime prodrugs was successfully applied [31,32].

Based on the above-mentioned data and in continuation of our research on 1,2,3-triazolyl- appended heterocycles [26,33,34,35] and their amidino derivatives [8,36,37] as anticancer and antipathogenic agents, herein, we report the synthesis and antiproliferative evaluations of novel indole, quinoline and coumarin-based aromatic amidines **1**–**11** and amidoximes **12**–**22**. Since nucleic acids are a major target for a large number of anticancer drugs, one of our aims was to investigate the binding of small molecules to DNA and RNA [29]. Assessing the binding strength and mode of binding of small molecule-DNA/RNA interactions was performed using UV–Vis, fluorescence and circular dichroism (CD) spectroscopy.

## 2. Results and Discussion

### 2.1. Chemistry

**Scheme 1:**
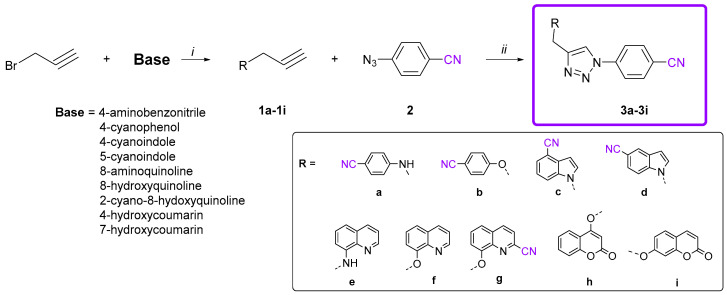
Synthesis of cyano-substituted 1,2,3-triazole derivatives. Reagents and conditions: (i) propargylation of hydroxyl group: propargyl bromide, acetone, K_2_CO_3_, reflux, 24 h; propargylation of amino group: propargyl bromide, DMF, K_2_CO_3_, 80 °C, 24 h; (ii) methanol, Cu(OAc)_2_, r.t., 24 h.

Novel hybrids of aromatic nitrile and heterocycle linked via 1,2,3-triazole scaffold **3a**–**3i** were synthesized by regioselective Cu(I)-catalyzed azide-alkyne 1,3-dipolar cycloaddition of 4-azidobenzonitrile and corresponding alkynes. Alkynyl derivatives of benzonitrile (**1a** and **1b**), indole (**1c** and **1d**), quinoline (**1e**–**1g**) and coumarin (**1h** and **1i**) were synthesized by alkylation with propargyl bromide in the presence of a base (Scheme 1), while azido derivative **2** was prepared by diazotation reaction according to the well-known procedure [38]. To study the influence of the type of linker between heterocycles on their antiproliferative activity, 1,2,3-triazolyl-appended aryl and quinoline analogues containing nitrogen- (**3a** and **3e**) and oxygen-containing (**3b**, **3f** and **3g**) aliphatic linkers were prepared. Coumarin was attached to aryl-triazole unit at C-4 (**3h**) and C-7 (**3i**) positions. 

**Scheme 2:**
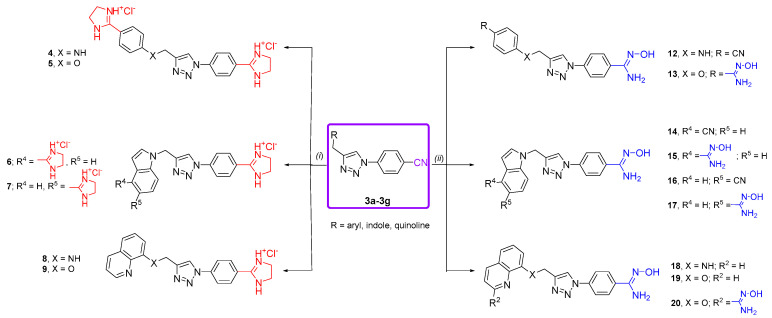
Synthesis of amidine- (**4**–**9**) and amidoxime-substituted (**12**–**20**) 1,2,3-triazolyl heterocycle derivatives. Reagents and conditions: (i) 1. HCl(g), abs. ethanol; 2. ethylenediamine (EDA), abs. ethanol, reflux, 24 h; 3. HCl(g), abs. ethanol; (ii) methanol:DMF = 2:1, Et_3_N, NH_2_OH⸱HCl, 100 °C, 6 h.

**Scheme 3:**
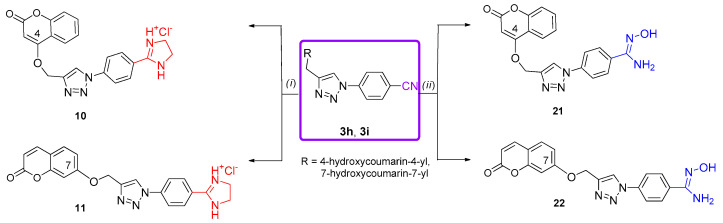
Synthesis of amidino- (**10** and **11**) and amidoxime-substituted (**12** and **22**) 1,2,3-triazolyl coumarin derivatives. Reagents and conditions: (i) 1. HCl(g), abs. ethanol; 2. EDA, abs. ethanol, reflux, 24 h; 3. HCl(g), abs. ethanol; (ii) methanol:DMF = 2:1, Et_3_N, NH_2_OH⸱HCl, 100 °C, 6 h.

Nitrile derivatives **3a**–**3i** were subsequently used as precursors for the synthesis of amidine- (**4**–**11**) and amidoxime-substituted (**12**–**22**) heterocycles (Scheme 2 and Scheme 3). Amidines were synthesized according to the Pinner method [39]. Treatment of corresponding cyano derivative **3a**–**3h** with dry gaseous HCl in absolute ethanol following by the reaction of the resulting imidate salt with ethylenediamine afforded targeted 2-imidazolinyl substituted derivatives **4**–**11**. Conversely, the reaction of nitriles **3a**–**3i** with hydroxylamine and triethylamine resulted in the desired amidoxime derivatives **12**–**22**. In the synthesis of amidoximes both mono- **12**, **14**, **16** and **19** and their bis-amidoxime analogues **13**, **15**, **17** and **20** were obtained (Scheme 2).

### 2.2. Biological Evaluation of Aromatic Amidines and Amidoximes 

#### 2.2.1. Evaluation of Antiproliferative Activity

Antiproliferative evaluations of amidine- and amidoxime-substituted heterocycles were performed on human tumor cell lines including cervical carcinoma (HeLa), colorectal adenocarcinoma, metastatic (SW620), lung adenocarcinoma (A549) and hepatocellular carcinoma (HepG2) and non-tumor HFF (human foreskin fibroblasts) cells (Table 1). 5-Fluorouracil (5-FU) was used as the reference drug.

**Figure 3:**
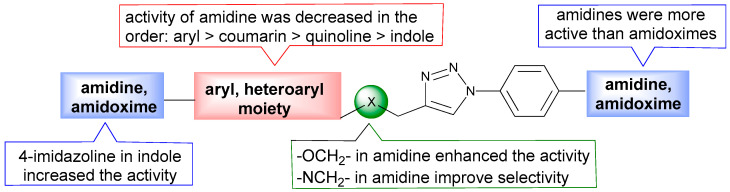
Structure-activity relationship of antiproliferative activity of the amidine- and amidoxime-substituted heterocycles.

From the amidine series, asymmetrical aryl bisamidine **5** linked via 4-methyleneoxy-1,2,3-triazolyl spacer showed strong antiproliferative activity on HeLa (IC_50_ = 0.80 μM), HepG2 (IC_50_ = 0.64 μM) and SW620 (IC_50_ = 0.22 μM) cell lines. Its analogue **4** bearing 4-aminomethylene-1,2,3-triazolyl spacer showed significantly reduced activity (Figure 3). Similarly, quinoline amidine **9** with 8-methyleneoxy-1,2,3-triazolyl unit exhibited 13-fold stronger inhibitory effects to tumor cells compared to its structural analogue **8**. The only exception was the growth-inhibition effect on HEPG2 cells that was 2-fold increased by **8** relative to its congener **9**. Conversely, aminomethylene units in amidines **4** and **8** contributed to their selectivity, showing less toxicity to normal HFF fibroblasts with selectivity index (SI) of 2 compared to amidines **5** and **9**, with methyleneoxy spacer, which showed high cytotoxicity to normal fibroblasts. Antiproliferative evaluations of indole diamidines showed that imidazoline 4-substituted indole **6** was favorable for the activity, particularly on HEPG2 cells (IC_50_ = 2.37 μM). Conversely, placement of the imidazoline in the 5-position reduced the cytostatic activity of compound **7**. Moreover, both 4- and 7-substituted coumarin amidines **10** and **11** exhibited strong cytostatic activity against all evaluated cell lines. However, 7-substituted coumarin **11** showed to be more selective compared to **10**, particularly on HepG2 cells with SI of 11.

Generally, antiproliferative evaluations of amidoximes **12**–**22** showed reduced antiproliferative activity compared to their corresponding amidine analogues **4**–**11**. Only moderate inhibitory activities of aryl amidoxime **12**, indole mono- **14** and diamidoximes **17** were observed on SW629 and HeLa cells. Coumarine amidoximes **21** and **22** were deprived of cytostatic activity, while quinoline amidoximes **18** and **20** displayed strong inhibitory effect on A549 (**18**, IC_50_ = 6.52 μM), HeLa (**20**, IC_50_ = 7.15 μM) and SW620 (**20**, IC_50_ = 7.24 μM) cells. Quinoline diamidoxime **20** was around 9-fold more active on HeLa and SW620 cells compared to its monoamidoxime **19**. Both quinolines **19** and **20** with methyleneoxy spacer did not exhibit an inhibitory effect on non-tumor HFF cells and selectivity was more expressed by inhibition of compound **20** on HeLa and SW620 cells (SI > 14). 

Comparison of the activity of evaluated compounds and reference drug (5-FU) showed that from amidine series, compounds **5**, **10** and **11** had better antiproliferative effect on HeLa and HepG2 cells, while from amidoximes, only compound **20** had activity comparable to that of 5-FU.

#### 2.2.2. DNA Binding Study

Based upon the antiproliferative activity, several compounds from a series of novel amidine- and amidoxime-substituted heterocycles (**5**, **6**, **8**–**11** and **20**) were selected for the study with *calf thymus* DNA (ctDNA), alternating AT-DNA (poly (dAdT)_2_) and AU-RNA (poly A-poly U). Compounds **5**, **6**, **8**–**11** were soluble in water (*c* = 2 × 10^−3^ mol dm^−3^). Due to the small aggregation of **20** in water, a stock solution was also prepared in DMSO (*c* = 2 × 10^−3^ mol dm^−3^) to check the dependence of UV–Vis changes on concentration increase. The absorbencies of buffered aqueous solutions of studied compounds were proportional to their concentrations up to *c* = 2 × 10^−5^ mol dm^−3^ (Supplementary information (SI), Figures S1–S8) indicating that studied compounds do not aggregate by intermolecular stacking at experimental conditions used. Absorption maxima and corresponding molar extinction coefficients (ε) are given in Table S1 (SI).

**Figure 4:**
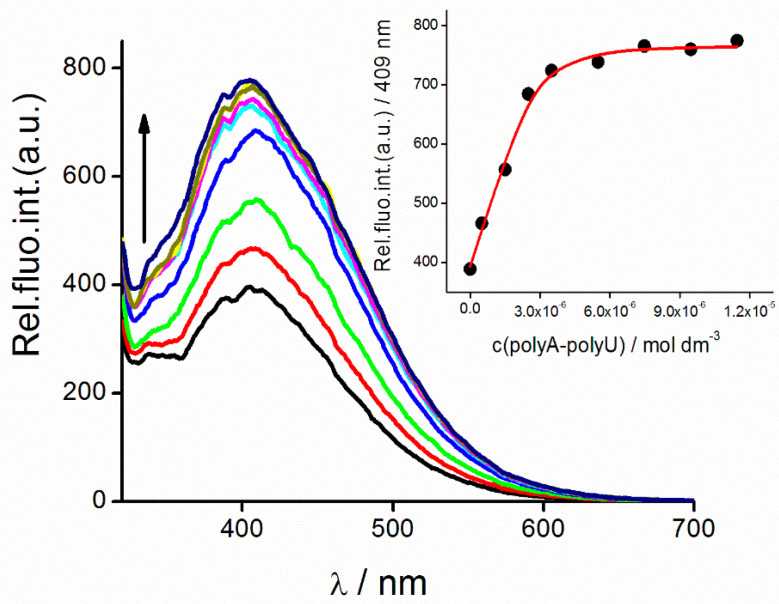
Changes in fluorescence spectrum of **5** (*c* = 2.0 × 10^−6^ mol dm^−3^, λ_exc_ = 300 nm) upon titration with poly A-poly U (*c* = 5.00 × 10^−7^–1.15 × 10^−5^ mol dm^−3^) with graph showing dependence of **5** absorbance at λ_max_ = 409 nm on c(poly A-poly U), at pH = 7, sodium cacodylate buffer, *I* = 0.05 mol dm^−3^.

The addition of ctDNA, poly A-poly U and poly (dAdT)_2_ yielded a fluorescence decrease of **6**, **9**, **11** and **20** whereas an emission increase was noticed in the case of **5, 8** and **10** with all studied polynucleotides (Table 2, Figure 4). The fluorescence changes of studied compounds were not dependent on the structure of polynucleotide added.

The binding constants *Ks* and ratios *n*_(bound compound)/(DNA/RNA)_ obtained by processing of fluorometric titration data with Scatchard equation [40] are summarized in Table 2. Studied compounds showed similar affinity toward ds-DNA and ds-RNA. Still, some compounds such as **5**, **6**, **9** and **10** showed greater affinities towards p(dAdT)_2_ compared to other compounds. In addition, **5** displayed the best affinity towards AU-RNA than other compounds. Fluorescence changes of **8** and **10** with ctDNA and poly A-poly U were too small for accurate calculation of binding constants. Compounds with cyclic amidines at both ends of heterocyclic core (**5**, **6**) or with cyclic amidine at one end and quinoline at the other (**9**) exhibited the preferential binding toward AT sequences. Interestingly, **9** exhibited greater affinities toward polynucleotides than **8**, although the only difference was the linker between triazole and quinoline rings, *O*-alkyl vs. *N*-alkyl linker. 

Compounds **9**–**11** and especially **5** and **6** showed moderate to big stabilization effect of AT-DNA (Table S2, Figure S41). In comparison to AT-DNA, stabilization of ctDNA by **5** and **6** was weaker as a consequence of the mixed base pair composition of ctDNA (58% AT, 42% GC). Other compounds showed either small or no stabilization effect of polynucleotides. Only a derivative possessing two imidazole moieties at the end of the heteroaromatic structure, **5** stabilized both DNA and RNA.

CD spectroscopy is a highly sensitive method for gaining insight into the conformational changes in the secondary structure of polynucleotides induced by small molecule binding [41]. In addition, it can provide information on the binding mode for small achiral molecules possessing UV–Vis spectra above 300 nm.

**Figure 5:**
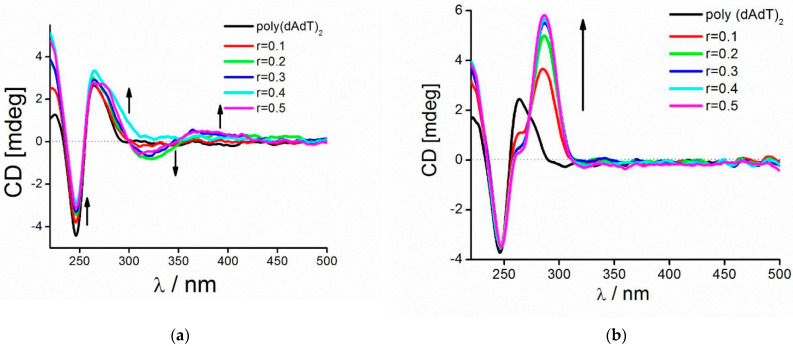
CD titration of poly (dAdT)_2_ (*c* = 3.0 × 10^−5^ mol dm^−3^) with (**a**) **11** and (**b**) **5** at molar ratios *r* = compound/polynucleotide (pH = 7.0, buffer sodium cacodylate, *I* = 0.05 mol dm^−3^).

Generally, the addition of compounds caused either a small decrease or a small increase of CD intensity of DNA and RNA polynucleotides at their maximal values (ctDNA at 275 nm, AT-DNA and AU-RNA at 260 nm). Still, significantly induced CD (ICD) bands appeared in titrations of **5**, **6** and **9**–**11** with AT-DNA (Figure 5, SI). Addition of AT-DNA to **6**, **9** and **11** induced positive ICD signals in the region from 270–303 nm at ratios, r < 0.2 which could be ascribed to minor groove binding [42]. However, positive and negative signals positioned at 320 and 380 nm appeared at ratios of r higher than 0.2, suggesting additional binding mode—formation and binding of dimers or higher-order aggregates along the polynucleotide backbone. In titrations with AT-DNA, compounds **5** and **10** caused strong positive ICD bands centered around 276 and 287 nm, respectively which is consistent with minor groove binding (Figure 5, SI). In titrations with AU-RNA with **10** and **11**, weak negative ICD signals visible around 300 nm are indicative of intercalative binding mode where the ligands are oriented “parallel” to the long axis of adjacent base pairs [42]. Compound **5** caused an appearance of similar ICD signals (strong positive ICD signals around 284 nm) with ctDNA as in titration with AT-DNA and additionally, positive and negative signals positioned at 320 and 390 nm were indicative of binding of aggregates along the polynucleotide backbone or most probably inside the groove.

In titrations of polynucleotides with **8** and **20**, there were no significantly induced CD signals possibly due to the formation of aggregates such as in titration of **20** with ctDNA or a lack of any dominant orientation of **8** and **20** molecules with respect to the polynucleotide chiral axis.

## 3. Experimental Part

### 3.1. Materials and Methods

All solvents were purified following recommended drying agents and/or distilled over 3 Å molecular sieves. For monitoring the progress of a reaction and for comparison purposes, thin layer chromatography (TLC) was performed on pre-coated *Merck* (Darmstadt, Germany) silica gel 60F-254 plates using an appropriate solvent system and the spots were detected under UV light (254 nm). For column chromatography silica gel (*Fluka*, Buchs, Switzerland, 0.063–0.2 mm) was employed, glass column was slurry-packed under gravity. Melting points (uncorrected) were determined with *Kofler* micro hot-stage (*Reichert*, Wien, Austria). The IR spectra were recorded on PerkinElmer Spectrum One spectrophotometer (Boston, MA, USA) with KBr disks. Elemental composition analyses of all novel compounds were within the 0.4% of the calculated values. ^1^H and ^13^C NMR spectra were acquired on a *Bruker* 300 and 600 MHz NMR spectrometer (Bruker Biospin, Rheinstetten, Germany). All data were recorded in DMSO-*d*_6_ at 298 K. Chemical shifts were referenced to the residual solvent signal of DMSO-*d*_6_ at δ 2.50 ppm for ^1^H and δ 39.50 ppm for ^13^C. Individual resonances were assigned on the basis of their chemical shifts, signal intensities, multiplicity of resonances and H–H coupling constants. ^1^H and ^13^C NMR spectra of compounds **4**–**22** are provided in Supporting Information (Figures S43–S61).

### 3.2. Synthetic Procedures

Compounds 4-azidobenzonitrile (**2**) [39] and alkynyl derivatives of benzonitrile (**1a** and **1b**) [43,44], quinoline (**1e** and **1f**) [45,46] and coumarin (**1h** and **1i**) [47] were synthesized according to the known procedure. 

### 3.3. Preparation of Alkynyl Derivatives ***1c***, ***1d*** and ***1g***

#### 3.3.1. 4-Cyano-1-((prop-2-ynyl)amino)-1*H*-indole (**1c**)

4-Cyanoindole (500 mg; 3.52 mmol) was dissolved in DMF (10 mL); K_2_CO_3_ (1.2 equiv.) was added and reaction mixture was heated at 80 °C. After 30 min, propargyl bromide (1.2 equiv.) was added. Reaction mixture was stirred about 24 h. Solvent was removed under reduced pressure and residue purified by column chromatography with dichloromethane as eluens. Compound **1c** was isolated as white powder (403 mg; 64%; m.p. = 137–140 °C). ^1^H NMR (300 MHz, DMSO-*d*_6_) (*δ*/ppm): 7.92 (d, *J* = 8.3 Hz, 1H, H-5), 7.72 (d, *J* = 3.2 Hz, 1H, H-2), 7.60 (dd, *J* = 7.4, 0.7 Hz, 1H, H-7), 7.39–7.31 (m, 1H, H-6), 6.63 (dd, *J* = 3.2, 0.8 Hz, 1H, H-3), 5.21 (d, *J* = 2.5 Hz, 2H, CH_2_), 3.46 (t, *J* = 2.5 Hz, 1H, CH). ^13^C NMR (151 MHz, DMSO-*d*_6_) (*δ*/ppm): 135.13 (C-7a), 131.70 (C-2), 129.10 (C-3a), 125.06 (C-5), 121.37 (C-6), 118.36 (CN), 115.59 (C-7), 101.67 C-4), 99.59 (C-3), 78.63 (C-2′), 76.04 (C-3′), 35.45 (CH_2_-1′).

#### 3.3.2. 5-Cyano-1-((prop-2-ynyl)amino)-1*H*-indole (**1d**)

5-Cyanoindole (1.00 g; 7.03 mmol) was dissolved in DMF (20 mL), K_2_CO_3_ (1.2 equiv was added and reaction mixture was heated at 80 °C. After 30 min propargyl bromide (1.2 equiv.) was added. Reaction mixture was stirred about 24 h. Solvent was removed under reduced pressure and residue purified by column chromatography with dichloromethane as eluens. Compound **1d** was isolated as pink powder (900 mg; 71%, m.p. = 107–109 °C). ^1^H NMR (300 MHz, DMSO-*d*_6_) (*δ*/ppm): 5.19 (d, *J* = 2.5 Hz, 2H, CH_2_), 3.44 (t, *J* = 2,4 Hz, 1H, CH), 7.54 (dd, *J* = 8.5, 1.4 Hz, 1H, H-6), 6.64 (d, *J* = 3.0 Hz, 1H, H-3), 7.62 (d, *J* = 3.2 Hz, 1H, H-2), 7.72 (d, *J* = 8.5, 1H, H-7), 8.12 (d, *J* = 0.8, 1H, H-4). ^13^C NMR (75 MHz, DMSO-*d*_6_) (*δ*/ppm): 137.02 (C-7a), 131.03 (C-2), 128.07 (C-3a), 126.15 (C-4), 124.11 (C-6), 120.48 (CN), 111.40 (C-7), 102.42 (C-3), 101.64 (C-5), 78.64 (C-2′), 76.01 (C-3′), 35.36 (CH_2_-1′).

#### 3.3.3. 2-Cyano-8-(prop-2-ynyloxy)-quinoline (**1g**)

To a solution of 8-hydroxy-2-carbonitrile (500 mg; 2.94 mmol) in acetone (18 mL), K_2_CO_3_ (6 equiv.) was added and reaction mixture was heated under reflux. After 30 min, propargyl bromide (0.4 mL; 2.94 mmol) was added. Reaction mixture was stirred and heated under reflux for about 24 h. Solvent was removed under reduced pressure and residue purified by column chromatography with petroleum ether:ethyl acetate = 6:4 as eluens. Compound **1g** was isolated as white powder (498 mg; 81%, m.p. = 144–147 °C). ^1^H NMR (600 MHz, DMSO-*d*_6_) (*δ*/ppm): 8.62 (d, *J* = 8.5 Hz, 1H, H-4), 8.05 (d, *J* = 8.4 Hz, 1H, H-3), 7.75 (t, *J* = 8.0 Hz, 1H, H-6), 7.69 (d, *J* = 7.5 Hz, 1H, H-5), 7.44 (d, *J* = 7.7 Hz, 1H, H-7), 5.10 (d, *J* = 2.3 Hz, 2H, CH_2_), 3.65 (t, *J* = 2.3 Hz, 1H, CH). ^13^C NMR (75 MHz, DMSO-*d*_6_) (δ/ppm): 152.62 (C-8), 139.53 (C-4a), 138.09 (C-4), 131.56 (C-8a), 130.00 (C-6), 129.77 (C-2), 124.32 (C-3), 120.28 (C-5), 117.72 (CN), 111.64 (C-7), 78.90 (C-3′), 78.68 (C-2′), 56.22 (C-1′).

### 3.4. General Procedure for Synthesis of Cyano-Substituted 1,2,3-Triazole Derivatives ***3a***–***3i***

4-Azidobenzonitrile (**2**) (1 equiv.) and corresponding alkyne **1a**–**1i** (1 equiv.) were dissolved in methanol, followed by addition of Cu(OAc)_2_ (0.05 equiv.). Reaction mixture was stirred for 24 h at room temperature. Solvent was removed under reduced pressure and residue purified by column chromatography with dichloromethane as eluent. 

#### 3.4.1. 4-((1-(4-Cyanophenyl)-1*H*-1,2,3-triazole-4-yl)methylamino)benzonitrile (**3a**)

Compound **3a** was prepared according to general procedure from 4-azidobenzonitrile (396 mg; 2.75 mmol) and compound **1a** (428 mg; 2.75 mmol) in methanol (30 mL). After purification by column chromatography with dichloromethane:methanol = 100:1 as eluens, compound **3a** was isolated as white powder (306.7 mg; 37%, m.p. = 247–248 °C). ^1^H NMR (300 MHz, DMSO-*d*_6_) (*δ*/ppm): 8.90 (s, 1H, CH-triazole), 8.12 (q, *J* = 9.0 Hz, 4H, Ph), 7.47 (d, *J* = 8.8 Hz, 2H, Ph), 7.25 (t, *J* = 5.6 Hz, 1H, NH), 6.75 (d, *J* = 8.8 Hz, 2H, Ph), 4,48 (d, *J* = 5.7 Hz, 2H, CH_2_). ^13^C NMR (75 MHz, DMSO-*d*_6_) (δ/ppm): 151.68 (Cq-Ph), 146.29 (Cq-Ph), 139.48 (Cq-triazole), 134.30 (C-Ph), 133.36 (C-Ph), 121.53 (CH-triazole), 120.46 (CN), 120.34 (C-Ph), 118.09 (CN), 112.19 (C-Ph), 110.97 (Cq-Ph), 96.36 (Cq-Ph), 37.64 (CH_2_).

#### 3.4.2. 4-((1-(4-Cyanophenyl)-1*H*-1,2,3-triazol-4-yl)methyloxy)benzonitrile (**3b**)

Compound **3b** was prepared according to general procedure from 4-azidobenzonitrile (250 mg; 1.74 mmol) and compound **1b** (273 mg; 1.74 mmol) in methanol (20 mL). Compound **3b** was isolated as white powder (418 mg; 80%, m.p. = 207–210 °C). ^1^H NMR (600 MHz, DMSO-*d*_6_) (*δ*/ppm): 9.13 (s, 1H, H-triazole), 8.14 (dd, *J* = 29.0, 8.8 Hz, 4H, Ph), 7.81 (d, *J* = 8.9 Hz, 2H, Ph), 7.26 (d, *J* = 8.9 Hz, 2H, Ph), 5.38 (s, 1H, CH_2_). ^13^C NMR (75 MHz, DMSO-*d*_6_) (δ/ppm): 161.36 (C-Ph), 143.66 (C-Ph), 139.44 (Cq-triazole), 134.36 (C-Ph), 134.31 (CH-triazole), 123.47 (C-Ph), 120.69 (C-Ph), 119.09 (CN), 118.11 (CN), 115.91 (C-Ph), 111.27 (C-Ph), 103.39 (C-Ph), 61.32 (CH_2_).

#### 3.4.3. 4-Cyano-1-((1-(4-cyanophenyl)-1*H*-1,2,3-triazol-4-yl)methylamino)-1*H*-indole (**3c**) 

Compound **3c** was prepared according to general procedure from 4-azidobenzonitrile (240 mg; 1.67 mmol) and compound **1c** (300 mg; 1.67 mmol) in methanol (20 mL). Compound **3c** was isolated as white powder (308 mg; 57%, m.p. = 200–203 °C). ^1^H NMR (300 MHz, DMSO-*d*_6_) (*δ*/ppm): 8.95 (s, 1H, H-triazole), 8.13–8.05 (m, 4H, H-5), 8.02 (d, *J* = 8.3 Hz, 1H, H-5), 7.80 (d, *J* = 3.2 Hz, 1H, H-2), 7.57 (d, *J* = 7.3 Hz, 1H, H-7), 7.36–7.27 (m, 1H, H-6), 6.65 (d, *J* = 2.7 Hz, 1H, H-3), 5.70 (s, 2H, CH_2_). ^13^C NMR (75 MHz, DMSO-*d*_6_) (δ/ppm): 144.65 (C-Ph), 139.36 (Cq-triazole), 135.31(C-7a), 134.23 (C-Ph), 132.14 (C-2), 129.08 (C-3a), 124.90 (C-5), 122.03 (CH-triazole), 121.28 (C-6), 120.54 (C-Ph), 118.46 (CN), 118.04 (CN), 115.73 (C-7), 111.10 (C-Ph), 101.54 (C-Ph), 99.53 (C-3), 40.93 (CH_2_).

#### 3.4.4. 5-Cyano-1-((1-(4-cyanophenyl)-1*H*-1,2,3-triazol-4-yl)methylamino)-1*H*-indole (**3d**) 

Compound **3d** was prepared according to general procedure from 4-azidobenzonitrile (250 mg; 1.74 mmol) and compound **1d** (313 mg; 1.74 mmol) in methanol (20 mL). Compound **3d** was isolated as white powder (513 mg; 91%, m.p. = 215–217 °C). ^1^H NMR (300 MHz, DMSO-*d*_6_) (*δ*/ppm): 8.96 (s, 1H, H-triazole), 8.22–8.04 (m, 5H, Ph, H-4), 7.83 (d, *J* = 8.6 Hz, 1H, H-7), 7.70 (d, *J* = 3.2 Hz, 1H, H-2), 7.51 (dd, *J* = 8.6, 1.5 Hz, 1H, H-6), 6.65 (d, *J* = 3.2 Hz, 1H, H-3), 5.67 (s, 2H, CH_2_). ^13^C NMR (75 MHz, DMSO-*d*_6_) (δ/ppm): 144.58 (C-Ph), 139.36 (Cq-triazole), 137.20 (C-7a), 134.23 (C-Ph), 131.44 (C-2), 128.03 (C-3a), 126.05 (C-4), 124.00 (C-6), 122.05 (CH-triazole), 120.54 (C-Ph), 118.03 (CN), 111.51 (C-7), 111.11 (C-Ph), 102.37 (C-3), 101.43 (C-5), 40.83 (CH_2_).

#### 3.4.5. 8-((1-(4-Cyanophenyl)-1*H*-1,2,3-triazol-4-yl)methylamino)quinoline (**3e**)

Compound **3e** was prepared according to general procedure from 4-azidobenzonitrile (209 mg; 1.45 mmol) and compound **1e** (316 mg; 1.74 mmol) in methanol (20 mL). After purification by column chromatography with dichloromethane:methanol = 100:1 as eluens, compound **3e** was isolated as yellow crystalline powder (269 mg; 60%, m.p. = 196–198 °C). ^1^H NMR (300 MHz, DMSO-*d*_6_) (*δ*/ppm): 8.90 (s, 1H, H-triazole), 8.76 (dd, *J* = 4.2, 1.6 Hz, 1H, H-2), 8.22 (dd, *J* = 8.3, 1.6 Hz, 1H, H-4), 8.10 (dd, *J* = 24.2, 8.9 Hz, 4H), 7.51 (dd, *J* = 8.3, 4.2 Hz, 1H, H-3), 7.36 (t, *J* = 7.9 Hz, 1H, H-6), 7.10 (d, *J* = 8.2 Hz, 1H, H-7), 7.05 (t, *J* = 6.0 Hz, 1H, NH), 6.80 (d, *J* = 7.6 Hz, 1H, H-5), 4.68 (d, *J* = 6.0 Hz, 2H, CH_2_). ^13^C NMR (151 MHz, DMSO-*d_6_*) (δ/ppm): 147.05 (C-2), 143.89 (C-8), 139.55 (Cq-triazole), 137.58 (C-4a), 135.95 (C-4), 134.22 (C-Ph), 128.23 (C-8a), 127.67 (C-6), 121.75 (C-3), 121.30 (CH-triazole), 120.37 (C-Ph), 118.09 (CN), 113.91 (C-5), 110.87 (C-Ph), 104.89 (C-7), 38.28 (CH_2_).

#### 3.4.6. 8-((1-(4-Cyanophenyl)-1*H*-1,2,3-triazol-4-yl)methyloxy)quinoline (**3f**) 

Compound **3f** was prepared according to general procedure from 4-azidobenzonitrile (353 mg; 2.45 mmol) and compound **1f** (538 mg; 2.94 mmol) in methanol (15 mL). Compound **3f** was isolated as white powder (655 mg; 82%, m.p. = 158–162 °C). ^1^H NMR (300 MHz, DMSO-*d*_6_) (*δ*/ppm): 9.19 (s, 1H, H-triazole), 8.84 (dd, *J* = 4.1, 1.7 Hz, 1H, H-2), 8.33 (dd, *J* = 8.3, 1.7 Hz, 1H, H-7), 8.16 (dd, *J* = 23.9, 8.9 Hz, 4H, Ph), 7.65–7.39 (m, 4H, H-3, H-5, H-6, H-7), 5,47 (s, 2H, CH_2_). ^13^C NMR (75 MHz, DMSO-*d_6_*) (δ/ppm): 153.71 (C-8), 149.04 (C-2), 144.26 (C-Ph), 139.73 (C-4a), 139.47 (Cq-triazole), 135.82 (C-4), 134.31 (C-Ph), 129.09 (C-8a), 126.73 (C-6), 123.47 (CH-triazole), 121.90 (C-3), 120.59 (C-Ph), 120.30 (C-5), 118.08 (CN), 111.16 (C-Ph), 110.31 (C-7), 61.67 (CH_2_).

#### 3.4.7. 2-Cyano-8-((1-(4-cyanophenyl)-1*H*-1,2,3-triazol-4-yl)methyloxy)quinoline (**3g**) 

Compound **3g** was prepared according to general procedure from 4-azidobenzonitrile (187 mg; 1.30 mmol) and compound **1g** (270 mg; 1.30 mmol) in methanol (20 mL). Compound **3g** was isolated as white powder (260 mg; 57%, m.p. = 263–265 °C). ^1^H NMR (300 MHz, DMSO-*d*_6_) (*δ*/ppm): 9,21 (s, 1H, H-triazole), 8.62 (d, *J* = 8.5 Hz, 1H, H-4), 8.15 (dd, *J* = 21.8, 8.9 Hz, 4H, Ph), 8.05 (d, *J* = 8.5 Hz, 1H, H-3), 7.60–7.80 (m, 3H, H-5, H-5, H-7), 5,54 (s, 2H, CH_2_). ^13^C NMR (75 MHz, DMSO-*d*_6_) (δ/ppm): 153.47 (C-8), 143.74 (C-Ph), 139.56 (C-4a), 139.45 (Cq-triazole), 138.07 (C-4), 134.27 (C-Ph), 131.43 (C-8a), 130.21 (C-6), 129.84 (C-2), 124.33 (C-3), 123.73 (CH-triazole), 120.66 (C-Ph), 120.07 (C-5), 118.06 (CN), 117.74 (CN), 111.49 (C-7), 111.18 (Ph), 61.66 (CH_2_).

#### 3.4.8. 4-((1-(4-Cyanophenyl)-1*H*-1,2,3-triazol-4-yl)methyloxy)coumarin (**3h**) 

Compound **3h** was prepared according to general procedure from 4-azidobenzonitrile (158 mg; 1.10 mmol) and compound **1h** (220 mg; 1.10 mmol) in methanol (20 mL). Compound **3h** was isolated as white powder (245 mg; 71%, m.p. = 274–276 °C). ^1^H NMR (600 MHz, DMSO-*d*_6_) (*δ*/ppm): 9.23 (s, 1H, H-triazole), 8.17 (dd, *J* = 43.7, 8.4 Hz, 4H, Ph), 7.84 (d, *J* = 7.8 Hz, 1H, H-5), 7.67 (t, J = 7.7 Hz, 1H, H-7), 7.42 (d, *J* = 8.3 Hz, 1H, H-8), 7.36 (t, *J* = 7.6 Hz, 1H, H-6), 6,21 (s, 1H, H-3), 5.55 (s, 2H, CH_2_). ^13^C NMR (75 MHz, DMSO-*d*_6_) (δ/ppm): 164.28 (C-4), 161.50 (C-2), 152.77 (C-8a), 142.80 (C-Ph), 139.40 (Cq-triazole), 134.32 (C-Ph), 132.88 (C-7), 124.22 (C-6), 123.58 (CH-triazole), 123.02 (C-5), 120.70 (C-Ph), 118.07 (CN), 116.47 (C-8), 115.01 (C-4a), 111.29 (C-Ph), 91.53 (C-3), 62.71 (CH_2_).

#### 3.4.9. 7-((1-(4-Cyanophenyl)-1*H*-1,2,3-triazol-4-yl)methyloxy)coumarin (**3i**) 

Compound **3i** was prepared according to general procedure from 4-azidobenzonitrile (187 mg; 1.30 mmol) and compound **1i** (260 mg; 1.30 mmol) in methanol (20 mL). Compound **3i** was isolated as white powder (187 mg; 41.8%, m.p. = 228–229 °C). ^1^H NMR (300 MHz, DMSO-*d*_6_) (*δ*/ppm): 9.16 (s, 1H, H-triazole), 8.14 (td, *J* = 8.9, 2.0 Hz, 4H, Ph), 8.01 (d, *J* = 9.5 Hz, 1H, H-4), 7.66 (d, *J* = 8.7 Hz, 1H, H-5), 7.20 (d, *J* = 2.3 Hz, 1H, H-8), 7.06 (dd, *J* = 8.6, 2.4 Hz, 1H, H-6), 6.32 (d, *J* = 9.5 Hz, 1H, H-3), 5.39 (s, 2H, CH_2_). ^13^C NMR (151 MHz, DMSO-*d*_6_) (δ/ppm): 160.97 (C-2), 160.26 (C-7), 155.31 (C-8a), 144.30 (C-4), 143.67 (C-Ph), 139.43 (Cq-triazole), 134.34 (C-Ph), 129.60 (C-5), 123.47 (CH-triazole), 120.63 (C-Ph), 118.10 (CN), 112.92 (C-3), 112.83 (C-6), 112.76 (C-Ph), 111.22 (C-4a), 101.64 (C-8), 61.52 (CH_2_).

### 3.5. General Procedure for Synthesis of Imidazolinyl-Substituted 1,2,3-Triazole Derivatives ***4***–***11***

Suspension of corresponding cyano derivative **3a**–**3i** in absolute EtOH was cooled in an ice-salt bath and was saturated with HCl gas. The flask was sealed, and the mixture was stirred at room temperature until the nitrile band disappeared (monitored by the IR analysis at 2200 cm^−1^). The reaction mixture was diluted with dry diethylether, and the crude imidate was filtered off and suspended in absolute ethanol. Ethylenediamine was added and the mixture was stirred at reflux for 24 h. The reaction mixture was diluted with dry diethylether, and the crude base was filtered off. Suspension of base in absolute EtOH was cooled in an ice-salt bath and saturated with HCl gas, sealed and stirred for 24 h. Mixture was diluted with dry diethyl ether and the crude product was then filtered off, washed with diethylether to give powdered product as hydrochloride salt.

#### 3.5.1. 1-(4-(4,5-Dihydro-1*H*-imidazol-2-yl)-phenyl)-4-((4-(4,5-dihydro-1*H*-imidazol-2-yl)phenyl)aminomethyl)-1*H*-1,2,3-triazole dihydrochloride (**4**)

Compound **4** was prepared according to general procedure using **3a** (50 mg; 0.17 mmol) in absolute ethanol (15 mL). After 4 days, iminoester was filtered and suspended in absolute ethanol (10 mL) followed by the addition of EDA (0.07 mL, 1.02 mmol). Compound **4** was isolated as yellow powder (37 mg; 47%; m.p. > 250 °C). ^1^H NMR (300 MHz, DMSO-*d*_6_) (*δ*/ppm): 10.98 (s, 2H, NH), 10.12 (s, 2H, NH), 8.99 (s, 1H, H-triazole), 8.18–8.32 (m, 4H, Ph), 7.81 (d, *J =* 8.7 Hz, 2H, Ph), 7.57 (bs, 1H, NH), 6.83 (d, *J =* 8.8 Hz, 2H, Ph), 4.54 (d, *J =* 3,4 Hz, 2H, CH_2_), 4.02 (s, 4H, CH_2_), 3.89 (s, 4H, CH_2_). ^13^C NMR (75 MHz, DMSO-*d*_6_) (*δ*/ppm): 164.16 (C-imidazolyl), 163.86 (C-imidazolyl), 153.29 (C-1′), 153.46 (C-Ph), 146.13 (C-4), 140.33 (Cq-triazole), 130.71 (C-2,6), 130.57 (C-3′,5′), 121.81 (C-1), 121.61 (CH-triazole), 119.94 (C-3′,5′), 112.87 (C-2′,6′), 107.80 (C-4′), 44.38 (2 × CH_2_), 43.74 (2 × CH_2_), 37.60 (CH_2_). Anal. calcd. for C21H22N8 × 2 HCl × 1.5 H_2_O (Mr = 486.40): C 51.86, H 5.60, N 23.04; found: C 51.79, H 5.68, N 23.09%. 

#### 3.5.2. 1-(4-(4,5-Dihydro-1*H*-imidazol-2-yl)-phenyl]-4-((4-(4,5-dihydro-1*H*-imidazol-2-yl) phenyloxy)methyl]-1*H*-1,2,3-triazole dihydrochloride (**5**) 

Compound **5** was prepared according to general procedure using **3b** (100 mg; 0.33 mmol) in absolute ethanol (20 mL). After 10 days, iminoester was filtered and suspended in absolute ethanol (20 mL), followed by the addition of EDA (0.13 mL, 1.98 mmol). Compound **5** was isolated as white powder (34 mg; 22%); m.p. > 250 °C). ^1^H NMR (600 MHz, DMSO-*d*_6_) (*δ*/ppm): 11.06 (bs, 2H, NH-imidazolyl), 10.68 (bs, 2H, NH-imidazolyl), 9.23 (s, 1H, H-triazole), 8.24–8.36 (m, 4H, Ph), 8.11 (d, *J =* 9.0 Hz, 2H, Ph), 7.35 (d, *J =* 9.0 Hz, 2H, Ph), 5.43 (s, 2H, CH_2_), 4.03 (s, 4H, CH_2_), 3.96 (s, 4H, CH_2_). ^13^C NMR (151 MHz, DMSO-*d*_6_) (*δ*/ppm): 164.01 (C-imidazolyl), 163.69 (C-imidazolyl), 162.56 (C-1′), 143.58 (C-4), 140.26 (Cq-triazole), 130.93 (C-2,6), 130.72 (C-3′,6′), 123.51 (CH-triazole), 122.07 (C-1), 120.25 (C-3,5), 115.42 (C-2′,6′), 114.54 (C-4′), 61.36 (CH_2_), 44.41 (2 × CH_2_), 44.11 (2 × CH_2_). Anal. calcd. for C21H21N7O × 2 HCl × 3 H_2_O (Mr = 514.41): C 49.03, H 5.68, N 19.06; found: C 49.10, H 5.66, N 18.98%.

#### 3.5.3. 1-((1-(4-(Dihydro-1*H*-imidazol-2-yl)phenyl)-1*H*-1,2,3-triazol-4-yl)methyl)-4-(dihydro-1*H*-imidazol-2-yl)-1*H*-indole dihydrochloride (**6**)

Compound **6** was prepared according to general procedure using **3c** (150 mg; 0.46 mmol) in absolute ethanol (30 mL). After 4 days, iminoester was filtered and suspended in absolute ethanol (15 mL), followed by the addition of EDA (0.19 mL, 2.76 mmol). Compound **6** was isolated as brown powder (57 mg; 26%; m.p. > 250 °C). ^1^H NMR (300 MHz, DMSO-*d*_6_) (*δ*/ppm): 10.92 (s, 2H, NH-imidazolyl), 10.30 (s, 2H, NH-imidazolyl), 9.06 (s, 1H, H-triazole), 8.23 (m, 4H, Ph), 8.10 (d, *J =* 8.3 Hz, 1H, H-5), 7.84 (d, *J =* 3.3 Hz, 1H, H-2), 7.59 (d, *J =* 6.8 Hz, 1H, H-7), 7.36–7.43 (m, 1H, H-6), 6.79 (d, *J =* 2.6 Hz, 1H, H-3), 5.73 (s, 2H, CH_2_), 4.04 (s, 4H, 2 × CH_2_), 4.02 (s, 4H, 2 × CH_2_). ^13^C NMR (75 MHz, DMSO-*d*_6_) (*δ*/ppm): 165.66 (C-imidazolyl), 163.63 (C-imidazolyl), 144.51 (C-1′), 140.20 (Cq-triazole), 135.98 (C-7a), 131.87 (C-2), 130.71 (C-3′,5′), 125.68 (C-3a), 122.15 (CH-triazole), 121.97 (C-4), 121.60 (C-5), 120.89 (C-6), 120.13 (C-2′,6′), 116.39 (C-7), 113.76 (C-4′), 100.54 (C-3), 44.36 (CH_2_), 40.81 (CH_2_). Anal. calcd. for C23H22N8 × 2 HCl × 0.5 H_2_O (Mr = 492.41): C 56.10, H 5.12, N 22.76; found: C 56.98, H 5.14, N 22.81%.

#### 3.5.4. 1-((1-(4-(Dihydro-1*H*-imidazol-2-yl)phenyl)-1*H*-1,2,3-triazol-4-yl)methyl)-5-(dihydro-1*H*-imidazol-2-yl)-1*H*-indole dihydrochloride (**7**)

Compound **7** was prepared according to general procedure using **3d** (150 mg; 0.46 mmol) in absolute ethanol (30 mL). After 4 days, iminoester was filtered and suspended in absolute ethanol (15 mL), followed by the addition of EDA (0.19 mL, 2.76 mmol). Compound **7** was isolated as red powder (160 mg; 72%; m.p. = 228–231 °C). ^1^H NMR (300 MHz, DMSO-*d*_6_) (*δ*/ppm): 11.08 (s, 2H, NH-imidazolyl), 10.60 (s, 2H, NH-imidazolyl), 9.09 (s, 1H, H-triazole), 8.40 (s, 1H, H-4), 8.14–8.34 (m, 4H, Ph), 7.81–7.94 (m, 2H, H-2, H-7), 7.74 (d, *J =* 3.2 Hz, 1H, H-6), 6.71 (d, *J =* 3.1 Hz, 1H, H-3), 5.70 (s, 2H, CH_2_), 3.87–4.16 (m, 8H, 4xCH_2_). ^13^C NMR (151 MHz, DMSO-*d*_6_) (*δ*/ppm): 165.60 (C-imidazolyl), 163.62 (C-imidazolyl), 144.52 (C-1′), 140.20 (Cq-triazole), 138.37 (C-7a), 131.59 (C-2), 130.67 (C-3′,5′), 127.84 (C-3a), 122.63 (C-4), 122.14 (CH-triazole), 121.95 (C-4), 121.17 (C-6), 120.11 (C-2′,6′), 112.88 (C-5), 111.01 (C-7), 102.94 (C-3), 44.35 (2 × CH_2_), 44.06 (2 × CH_2_), 40.84 (CH_2_). Anal. calcd. for C23H22N8 × 2 HCl × 2.2 H_2_O (Mr = 523.03): C 52.82, H 5.47, N 21.46; found: C 52.62, H 5.45, N 21.53%.

#### 3.5.5. 8-(((1-(4-(4,5-Dihydro-1*H*-imidazol-2-yl)phenyl)-1*H*-1,2,3-triazol-4-yl)methyl)amino)-quinoline hydrochloride (**8**)

Compound **8** was prepared according to general procedure using **3e** (190 mg; 0.58 mmol) in absolute ethanol (30 mL). After 10 days iminoester was filtered and suspended in absolute ethanol (15 mL), followed by the addition of EDA (0.12 mL, 1.74 mmol). Compound **8** was isolated as white powder (48 mg; 21%; m.p. = 192–196 °C). ^1^H NMR (300 MHz, DMSO-*d*_6_) (*δ*/ppm): 10.93 (s, 2H, NH-imidazolyl), 8.96 (s, 1H, H-triazole), 8.82 (dd, *J =* 4.3, 1.5 Hz, 1H, H-2), 8.37 (d, *J =* 8.3 Hz, 1H, H-4), 8.18–8.30 (m, 4H, Ph), 7.60 (dd, *J =* 8.3, 4.3 Hz, 1H, H-3), 7.42 (t, *J =* 7.9 Hz, 1H, H-6), 7.19 (d, *J =* 8.0 Hz, 1H, H-7), 6.89 (d, *J =* 7.6 Hz, 1H, H-5), 4.71 (s, 2H, CH_2_), 4.01 (s, 4H, CH_2_). ^13^C NMR (75 MHz, DMSO-*d*_6_) (*δ*/ppm): 163.77 (C-imidazolyl), 146.90 (C-8), 146.42 (C-2), 143.14 (C-4a), 140.44 (Cq-triazole), 137.59 (C-4), 130.59 (C-3′,5′), 128.52 (C-8a), 128.12 (C-6), 121.79 (C-3), 121.72 (C-4′), 121.37 (CH-triazole), 120.00 (C-2′,6′), 114.25 (C-7), 106.00 (C-5), 44.41 (2 × CH_2_), 36.49 (CH_2_). Anal. calcd. for C21H19N7 × HCl × 2.5 H_2_O (Mr = 450.93): C 55.94, H 5.59, N 21.74; found: C 55.75, H 5.60, N 21.78%.

#### 3.5.6. 8-((1-(4-(4,5-Dihydro-1*H*-imidazol-2-yl)phenyl)-1*H*-1,2,3-triazol-4-yl)methyloxy)-quinoline hydrochloride (**9**)

Compound **9** was prepared according to general procedure using **3f** (175 mg; 0.53 mmol) in absolute ethanol (30 mL). After 6 days iminoester was filtered and suspended in absolute ethanol (15 mL), followed by the addition of EDA (0.11 mL, 1.59 mmol). Compound **9** was isolated as white powder (50 mg; 23%; m.p. = 229–232 °C). ^1^H NMR (300 MHz, DMSO-*d*_6_) (*δ*/ppm): 10.93 (s, 2H, NH-imidazolyl), 9.56 (s, 1H, H-triazole), 9.08 (d, *J =* 4.3 Hz, 1H, H-2), 8.91 (d, *J =* 7.3 Hz, 1H, H-4), 8.24–8.37 (m, 4H, Ph), 7.93 (bs, 1H, H-3), 7.76–7.86 (m, 3H, H-5, H-6, H-7), 5.64 (s, 2H, CH_2_), 4.04 (s, 4H, CH_2_). ^13^C NMR (151 MHz, DMSO-*d*_6_) (*δ*/ppm): 163.98 (C-imidazolyl), 148.54 (C-2), 144.02 (C-1′), 140.40 (Cq-triazole), 134.45 (C-4), 130.53 (C-3′,5′), 129.19 (C-8a), 127.20 (C-6), 123.48 (CH-triazole), 122.07 (C-3), 122.01 (C-4′), 120.40 (C-5), 120.30 (C-2′,6′), 110.84 (C-7), 61.87 (CH_2_), 44.50 (2 × CH_2_). Anal. calcd. for C21H18N6O × HCl × 3.8 H_2_O (Mr = 475.33): C 53.06, H 5.64, N 17.68; found: C 52.95, H 5.66, N 17.65%.

#### 3.5.7. 4-((1-(4-(4,5-Dihydro-1*H*-imidazol-2-yl)phenyl)-1*H*-1,2,3-triazol-4-yl)methyloxy)-coumarin hydrochloride (**10**)

Compound **10** was prepared according to general procedure using **3h** (100 mg; 0.29 mmol) in absolute ethanol (20 mL). After 10 days iminoester was filtered and suspended in absolute ethanol (10 mL), followed by the addition of EDA (0.06 mL, 0.87 mmol). Compound **10** was isolated as white powder (29 mg; 24%; m.p.> 250 °C). ^1^H NMR (300 MHz, DMSO-*d*_6_) (*δ*/ppm): 10.89 (s, 2H, NH-imidazolyl), 9.27 (s, 1H, H-triazole), 8.19–8.35 (m, 4H, Ph), 7.85 (d, *J =* 7.9 Hz, 1H, H-5), 7.68 (t, *J =* 7.1 Hz, 1H, H-7), 7.43 (d, *J =* 8.3 Hz, 1H, H-8), 7.36 (t, *J =* 7.6 Hz, 1H, H-6), 6.22 (s, 1H, H-3), 5.57 (s, 2H, CH_2_), 4.04 (s, 4H, 2xCH_2_). ^13^C NMR (75 MHz, DMSO-*d*_6_) (*δ*/ppm): 164.27 (C-4), 163.84 (C-imidazolyl), 161.49 (C-2), 152.78 (C-8a), 142.84 (C-1′), 140.29 (Cq-triazole), 132.89 (C-7), 130.57 (C-3′,5′), 124.23 (C-6), 123.52 (CH-triazole), 123.00 (C-5), 122.10 (C-4′), 120.39 (C-2′,6′), 116.48 (C-8), 115.01 (C-4a), 91.55 (C-3), 62.72 (CH_2_), 44.46 (2 × CH_2_). Anal. calcd. for C21H17N5O3 × HCl × 3.5 H_2_O (Mr = 486.91): C 51.80, H 5.18, N 14.38; found: C 51.71, H 5.16, N 14.40%.

#### 3.5.8. 7-((1-(4-(4,5-Dihydro-1*H*-imidazol-2-yl)phenyl)-1*H*-1,2,3-triazol-4-yl)methyloxy)-coumarin hydrochloride (**11**)

Compound **11** was prepared according to general procedure using **3i** (200 mg; 0.58 mmol) in absolute ethanol (30 mL). After 6 days iminoester was filtered and suspended in absolute ethanol (15 mL), followed by the addition of EDA (0.12 mL, 1.74 mmol). Compound **11** was isolated as white powder (72 mg; 29%; m.p. > 250 °C). ^1^H NMR (300 MHz, DMSO-*d*_6_) (*δ*/ppm): 10.98 (s, 2H, NH-imidazolyl), 9.19 (s, 1H, H-triazole), 8.27 (s, 4H, Ph), 8.02 (d, *J =* 9.5 Hz, 1H, H-4), 7.67 (d, *J =* 8.7 Hz, 1H, H-5), 7.20 (d, *J =* 2.2 Hz, 1H, H-8), 7.07 (dd, *J =* 8.6, 2.4 Hz, 1H, H-6), 6.32 (d, *J =* 9.5 Hz, 1H, H-3), 5.41 (s, 2H, CH_2_), 4.03 (s, 4H, CH_2_). ^13^C NMR (75 MHz, DMSO-*d*_6_) (*δ*/ppm): 163.91 (C-imidazolyl), 160.97 (C-2), 160.26 (C-7), 155.31 (C-8a), 144.30 (C-4), 143.74 (C-1′), 140.32 (Cq-triazole), 130.54 (C-3′,5′), 129.63 (C-5), 123.40 (CH-triazole), 122.12 (C-4′), 120.36 (C-2′,6′), 112.92 (C-3), 112.84 (C-6), 112.78 (C-4a), 101.66 (C-8), 61.55 (CH_2_), 44.53 (2 × CH_2_). Anal. calcd. for C21H17N5O3 × HCl × 1.3 H_2_O (Mr = 447.28): C 56.39, H 4.64, N 15.66; found: C 56.58, H 4.63, N 15.69%.

### 3.6. General Procedure for Synthesis of Amidoxime-Substituted 1,2,3-Triazole Derivatives ***12***–***22***

Corresponding cyano derivative **3a**–**3i** was suspended in solvent mixture methanol:dimethylformamide = 2:1, and suspension was heated at 100 °C. Triethylamine (Et_3_N) (3 equiv. for bis cyano derivatives **3a**–**3d** and **3g**, and 6 equiv. for cyano derivatives **3e**, **3f**, **3h** and **3i**) and NH_2_OH⸱HCl (3 equiv. for bis cyano derivatives **3a**–**3d** and **3g**, and 6 equiv. for cyano derivatives **3e**, **3f**, **3h** and **3i**) were added and reaction mixture was stirred for 6 h. After cooling, mixture was diluted with water and precipitate was filtered off. If necessary, precipitate was purified by column chromatography with dichloromethane:methanol = 10:1 as eluent.

#### 3.6.1. 4-(((1-(4-((*Z*)-*N*′-Hydroxycarbamimidoyl)phenyl)-1*H*-1,2,3-triazol-4-yl)methyl)amino)benzonitrile (**12**)

Compound **12** was prepared according to general procedure using **3a** (50 mg; 0.17 mmol), Et_3_N (0.14 mL) and NH_2_OH⸱HCl (71 mg) in solvent mixture methanol:DMF = 2:1 (6 mL). After purification by column chromatography compound **12** was isolated as white powder (19 mg; 35%; m.p. = 222–225 °C). ^1^H NMR (300 MHz, DMSO-*d*_6_) (*δ*/ppm): 9.79 (s, 1H, OH), 8.77 (s, 1H, H-triazole), 7.76–7.95 (m, 4H, Ph), 7.47 (d, *J =* 8.7 Hz, 2H, Ph), 7.23 (t, *J =* 5.6 Hz, 1H, NH), 6.76 (d, *J =* 8.8 Hz, 2H, Ph), 5.93 (s, 2H, NH_2_), 4.46 (d, *J =* 5.6 Hz, 2H, CH_2_). ^13^C NMR (75 MHz, DMSO-*d*_6_) (*δ*/ppm): 151.73 (C-1′), 149.83 (C(NH_2_)=NOH), 145.74 (C-4), 136.68 (Cq-triazole), 133.35 (C-3′,5′), 129.21 (C-1), 126.71 (C-2,6), 121.21 (CH-triazole), 120.49 (CN), 119.47 (C-3,5), 112.18 (C-2′,6′), 96.27 (C-4′), 37.71 (CH_2_). Anal. calcd. for C17H15N7O (Mr = 333.36): C 61.25, H 4.54, N 29.41; found: C 61.42, H 4.52, N 29.47%.

#### 3.6.2. (*Z*)-*N*′-Hydroxy-4-(4-((4-((*Z*)-*N*′-hydroxycarbamimidoyl)phenoxy)methyl)-1*H*-1,2,3-triazol-1-yl)benzimidamide (**13**)

Compound **13** was prepared according to general procedure using **3b** (80 mg; 0.27 mmol), Et_3_N (0.22 mL) and NH_2_OH⸱HCl (113 mg) in solvent mixture methanol:DMF = 2:1 (6 mL). After purification by column chromatography compound **13** was isolated as white powder (80 mg; 82%; m.p. = 218–221 °C). ^1^H NMR (300 MHz, DMSO-*d*_6_) (*δ*/ppm): 9.75 (s, 1H, OH), 9.43 (s, 1H, OH), 8.95 (s, 1H, H-triazole), 7.80–8.03 (m, 4H, Ph), 7.63 (d, *J =* 8.8 Hz, 2H, Ph), 7.08 (d, *J =* 8.8 Hz, 2H, Ph), 5.89 (s, 2H, NH_2_), 5.70 (s, 2H, NH_2_), 5.28 (s, 2H, CH_2_). ^13^C NMR (75 MHz, DMSO-*d*_6_) (*δ*/ppm): 158.51 (C-Ph), 150.55 (C(NH_2_)=NOH), 149.84 (C(NH_2_)=NOH), 143.83 (C-Ph), 136.62 (Cq-triazole), 133.52 (C-Ph), 126.76 (C-Ph), 126.73 (C-Ph), 126.15 (C-Ph), 122.82 (CH-triazole), 119.69 (C-Ph), 114.27 (C-Ph), 61.02 (CH_2_). Anal. calcd. for C17H17N7O3 (Mr = 367.37): C 55.58, H 4.66, N 26.69; found: C 55.41, H 4.66, N 26.63%.

#### 3.6.3. 1-((1-(4-((*Z*)-*N*′-Hydroxycarbamimidoyl)phenyl)-1*H*-1,2,3-triazol-4-yl)methyl)-4-cyanoindole (**14**) and (*Z*)-*N′*-hydroxy-1-((1-(4-((*Z*)-*N*′-hydroxycarbamimidoyl)phenyl)-1*H*-1,2,3-triazol-4-yl)methyl)-1*H*-indole-4-carboximidamide (**15**)

Compounds **14** and **15** were prepared according to general procedure using **3c** (150 mg; 0.46 mmol), Et_3_N (0.38 mL) and NH_2_OH⸱HCl (192 mg) in solvent mixture methanol:DMF = 2:1 (12 mL). Purification by column chromatography yielded compound **14** as white powder (36 mg; 22%; m.p. = 229–232 °C) and compound **15** as white powder (74 mg; 41%; m.p. = 120–123 °C). 

**14**: ^1^H NMR (300 MHz, DMSO-*d*_6_) (*δ*/ppm): 9.79 (s, 1H, OH), 8.83 (s, 1H, H-triazole), 8.04 (d, *J =* 8.3 Hz, 1H, H-5), 7.86 (s, 4H, Ph), 7.81 (d, *J =* 3.2 Hz, 1H, H-2), 7.57 (d, *J =* 7.3 Hz, 1H, H-7), 7.25–7.37 (m, 1H, H-6), 6.64 (d, *J =* 3.1 Hz, 1H, H-3), 5.93 (s, 2H, NH_2_), 5.68 (s, 2H, CH_2_). ^13^C NMR (151 MHz, DMSO-*d*_6_) (*δ*/ppm): 149.93 (C(NH_2_)=NOH), 144.26 (C-Ph), 136.61 (Cq-triazole), 135.39 (C-7a), 133.56 (C-3a), 132.20 (C-2), 129.15 (C-Ph), 126.77 (C-Ph), 124.97 (C-5), 121.76 (CH-triazole), 121.36 (C-6), 119.75 (C-Ph), 118.56 (CN), 115.81 (C-7), 101.59 (C-4), 99.56 (C-3), 41.04 (CH_2_). Anal. calcd. for C19H15N7O (Mr = 357.38): C 63.86, H 4.23, N 27.44; found: C 63.89, H 4.24, N 27.42%.

**15**: ^1^H NMR (600 MHz, DMSO-*d*_6_) (*δ*/ppm): 9.78 (s, 1H, OH), 9.57 (s, 1H, OH), 8.82 (s, 1H, H-triazole), 7.86 (s, 4H, Ph), 7.67 (d, *J =* 8.2 Hz, 1H, H-5), 7.49 (d, *J =* 3,2 Hz, 1H, H-2), 7.30 (dd, *J =* 7.3, 0.5 Hz, 1H, H-7), 7.09–7.21 (m, 1H, H-6), 6.88 (dd, *J =* 3.0, 0.5 Hz, 1H, H-3), 5.92 (s, 2H, NH_2_), 5.68 (s, 2H, NH_2_), 5.56 (s, 2H, CH_2_). ^13^C NMR (151 MHz, DMSO-*d*_6_) (*δ*/ppm): 152.02 (C(NH_2_)=NOH), 149.80 (C(NH_2_)=NOH), 144.59 (C-Ph), 136.55 (Cq-triazole), 135.89 (C-7a), 133.42 (C-3a), 128.70 (C-2), 126.65 (C-Ph), 125.61 (C-Ph), 125.55 (C-4), 121.53 (CH-triazole), 120.69 (C-6), 119.59 (C-Ph), 117.65 (C-7), 110.78 (C-5), 102.63 (C-3), 40.71 (CH_2_). Anal. calcd. for C19H18N8O2 (Mr = 390.41): C 58.45, H 4.65, N 28.70; found: C 58.40, H 4.65, N 28.72%.

#### 3.6.4. 1-((1-(4-((*Z*)-*N*′-Hydroxycarbamimidoyl)phenyl)-1*H*-1,2,3-triazol-4-yl)methyl)-5-cyanoindole (**16**) and (*Z*)-*N*′-hydroxy-1-((1-(4-((*Z*)-*N*′-hydroxycarbamimidoyl)phenyl)-1*H*-1,2,3-triazol-4-yl)methyl)-indole-5-carboximidamide (**17**)

Compounds **16** and **17** were prepared according to general procedure using **3d** (125 mg; 0.39 mmol), Et_3_N (0.32 mL) and NH_2_OH⸱HCl (163 mg) in solvent mixture methanol:DMF = 2:1 (12 mL). Purification by column chromatography yielded compound **16** as white powder (26 mg; 18%; m.p. = 232–234 °C) and compound **17** as white powder (67 mg; 44%; m.p. = 152–155 °C). 

**16**: ^1^H NMR (300 MHz, DMSO-*d*_6_) (*δ*/ppm): 9.79 (s, 1H, OH), 8.84 (s, 1H, H-triazole), 8.11 (d, *J =* 1.0 Hz, 1H, H-4), 7.80–7.88 (m, 5H, Ph, H-7), 7.71 (d, *J =* 3.2 Hz, 1H, H-2), 7.51 (dd, *J =* 8.6, 1.5 Hz, 1H, H-6), 6,65 (d, *J =* 3.2 Hz, 1H, H-3), 5,92 (s, 2H, NH_2_), 5,65 (s, 2H, CH_2_). ^13^C NMR (75 MHz, DMSO-*d*_6_) (*δ*/ppm): 150.03 (C(NH_2_)=NOH), 144.28 (C-Ph), 137.32 (Cq-triazole), 136.67 (C-7a), 133.61 (C-Ph), 131.57 (C-2), 128.18 (C-3a), 126.86 (C-Ph), 126.21 (C-4), 124.14 (C-6), 121.85 (CH-triazole), 120.72 (CN), 119.83 (C-Ph), 111.65 (C-7), 102.51 (C-3), 101.50 (C-5), 40.98 (CH_2_). Anal. calcd. for C19H15N7O (Mr = 357.38): C 63.86, H 4.23, N 27.44; found: C 63.79, H 4.24, N 27.51%.

**17**: ^1^H NMR (600 MHz, DMSO-*d*_6_) (*δ*/ppm): 9.78 (s, 1H, OH), 9.39 (s, 1H, OH), 8.83 (s, 1H, H-triazole), 7.71–7.95 (m, 5H), 7.60 (d, *J =* 8.7 Hz, 1H, 2H), 7.43–7.54 (m, 2H, H-4, H-6), 6.38–6.55 (m, 1H, H-3), 5.91 (s, 2H, NH_2_), 5.72 (s, 2H, NH_2_), 5.56 (s, 2H, CH_2_). ^13^C NMR (75 MHz, DMSO-*d*_6_) (*δ*/ppm): 151.91 (C(NH_2_)=NOH), 149.81 (C(NH_2_)=NOH), 144.59 (C-Ph), 136.57 (Cq-triazole), 135.90 (C-7a), 133.45 (C-Ph), 129.31 (C-2), 127.77 (C-3a), 126.68 (C-Ph), 124.78 (C-5), 121.58 (CH-triazole), 119.61 (C-Ph), 119.26 (C-4), 117.77 (C-6), 109.68 (C-7), 101.71 (C-3), 40.78 (CH_2_). Anal. calcd. for C19H18N8O2 (Mr = 390.41): C 58.45, H 4.65, N 28.70; found: C 58.51, H 4.66, N 28.61%.

#### 3.6.5. 8-(((1-(4-((*Z*)-*N*′-Hydroxycarbamimidoyl)phenyl)-1*H*-1,2,3-triazol-4-yl)methyl)amino)quinoline (**18**)

Compound **18** was prepared according to general procedure using **3e** (80 mg; 0.25 mmol), Et_3_N (0.10 mL) and NH_2_OH⸱HCl (51 mg) in solvent mixture methanol:DMF = 2:1 (9 mL). After purification by column chromatography, compound **18** was isolated as white powder (35 mg; 40%; m.p. = 181–183 °C). ^1^H NMR (300 MHz, DMSO-*d*_6_) (*δ*/ppm): 9.78 (s, 1H, OH), 8.77 (s, 1H, H-triazole), 8.22 (dd, *J =* 8.3, 1.6 Hz, 1H, H-2), 7.80–7.97 (m, 5H, Ph, H-4), 7.51 (dd, *J =* 8.3, 4.2 Hz, 1H, H-3), 7.37 (t, *J =* 7.9 Hz, 1H, H-6), 7.11 (d, *J =* 8.1 Hz, 1H, H-5), 7.02 (t, *J =* 5.8 Hz, 1H, NH), 6.82 (d, *J =* 7.0 Hz, 1H, H-7), 5.92 (s, 2H, NH_2_), 4.67 (d, *J =* 6.0 Hz, 2H, CH_2_). ^13^C NMR (75 MHz, DMSO-*d*_6_) (*δ*/ppm): 150.00 (C(NH_2_)=NOH), 147.19 (C-2), 146.62 (C-8), 144.01 (C-1′), 137.66 (Cq-triazole), 136.84 (C-4a), 136.07 (C-4), 133.36 (C-1′), 128.33 (C-8a), 127.80 (C-6), 126.80 (C-3′,5′), 121.88 (C-3), 121.09 (CH-triazole), 119.63 (C-2′,6′), 114.00 (C-5), 105.02 (C-7), 35.90 (CH_2_). Anal. calcd. for C19H17N7O (Mr = 359.39): C 63.50, H 4.77, N 27.28; found: C 63.45, H 4.76, N 27.27%.

#### 3.6.6. 8-((1-(4-((*Z*)-*N*′-Hydroxycarbamimidoyl)phenyl)-1*H*-1,2,3-triazol-4-yl)methoxy)quinoline (**19**) 

Compound **19** was prepared according to general procedure using **3f** (90 mg; 0.28 mmol), Et_3_N (0.12 mL) and NH_2_OH⸱HCl (58 mg) in solvent mixture methanol: DMF = 2:1 (9 mL). Compound **19** was isolated as white powder (78 mg; 79%; m.p. = 230–232 °C). ^1^H NMR (600 MHz, DMSO-*d*_6_) (*δ*/ppm): 9.81 (s, 1H, OH), 9.06 (s, 1H, H-triazole), 8.84 (dd, *J* = 4.1, 1.7 Hz, 1H, H-2), 8.33 (dd, *J =* 8.3, 1.6 Hz, 1H, H-4), 7.88–7.99 (m, 4H, Ph), 7.50–7.59 (m, 3H, H-3, H-5, H-6), 7.46 (dd, *J =* 6.3, 2.7 Hz, 1H, H-7), 5.95 (s, 2H, NH_2_), 5.46 (s, 2H, CH_2_). ^13^C NMR (151 MHz, DMSO-*d*_6_) (*δ*/ppm): 153.78 (C-8), 149.84 (C(NH_2_)=NOH), 149.02 (C-2), 143.80 (C-Ph), 139.74 (Cq-triazole), 136.65 (C-4a), 135.81 (C-4), 133.54 (C-Ph), 129.21 (C-6), 129.08 (C-8a), 126.74 (C-Ph), 123.13 (CH-triazole), 121.88 (C-3), 120.21 (C-5), 119.71 (C-Ph), 110.24 (C-7), 61.74 (CH_2_). Anal. calcd. for C19H16N6O2 (Mr = 360.38): C 63.33, H 4.48, N 23.32; found: C 63.21, H 4.49, N 23.33%.

#### 3.6.7. (*Z*)-*N*′-Hydroxy-8-((1-(4-((*Z*)-*N*′-hydroxycarbamimidoyl)phenyl)-1*H*-1,2,3-triazol-4-yl)methoxy)quinoline-2-carboximidamide (**20**)

Compound **20** was prepared according to general procedure using **3g** (80 mg; 0.23 mmol), Et_3_N (0.19 mL) and NH_2_OH⸱HCl (95 mg) in solvent mixture methanol: DMF = 2:1 (9 mL). Compound **20** was isolated as white powder (84 mg; 87%; m.p. = 238–241 °C). ^1^H NMR (600 MHz, DMSO-*d*_6_) (*δ*/ppm): 10.17 (s, 1H, OH), 9.81 (s, 1H, OH), 9.07 (s, 1H, H-triazol), 8.31 (d, *J* = 8.7 Hz, 1H), 7.99 (d, *J* = 8.6 Hz, 1H), 7.89–7.98 (m, 4H, Ph), 7.48–7.60 (m, 3H), 5.80–6.23 (m, 4H, 2 × NH_2_), 5,53 (s, 2H, CH_2_). ^13^C NMR (75 MHz, DMSO-*d*_6_) (*δ*/ppm): 153.58 (C-8), 149.85 (C(NH_2_)=NOH), 149.57 (C(NH_2_)=NOH), 148.58 (C-2), 144.07 (C-Ph), 138.30 (C-triazol), 136.65 (C-4a), 136.27 (C-4), 133.49 (C-Ph), 129.16 (C-8a), 127.18 (C-6), 126.74 (C-Ph), 122.90 (CH-triazol), 120.30 (C-3), 119.67 (C-Ph), 117.71 (C-5), 111.82 (C-7), 62.39 (CH_2_). Anal. calcd. for C20H18N8O3 (Mr = 418.42): C 57.41, H 4.34, N 26.78; found: C 57.50, H 4.34, N 26.74%.

#### 3.6.8. 4-((1-(4-((*Z*)-*N*′-Hydroxycarbamimidoyl)phenyl)-1*H*-1,2,3-triazol-4-yl)methoxy)coumarin (**21**)

Compound **21** was prepared according to general procedure using **3h** (100 mg; 0.29 mmol), Et_3_N (0.12 mL) and NH_2_OH⸱HCl (60 mg) in solvent mixture methanol:DMF = 2:1 (9 mL). Compound **21** was isolated as white powder (75 mg; 69%; m.p. = 235–237 °C). ^1^H NMR (300 MHz, DMSO-*d*_6_) (*δ*/ppm): 9.81 (s, 1H, OH), 9.11 (s, 1H, H-triazole), 7.94 (q, *J =* 8.9 Hz, 4H, Ph), 7,85 (d, *J =* 7,8 Hz, 1H, H-5), 7,61–7,72 (m, 1H, H-7), 7,42 (d, *J =* 8,2 Hz, 1H, H-8), 7.35 (t, *J =* 7.6 Hz, 1H, H-6), 6.21 (s, 1H, H-3), 5.96 (s, 2H, NH_2_), 5.53 (s, 2H, CH_2_). ^13^C NMR (151 MHz, DMSO-*d*_6_) (*δ*/ppm): 164.32 (C-4), 161.50 (C-2), 152.76 (C-8a), 149.79 (C(NH_2_)=NOH), 142.33 (C-triazole), 136.54 (C-Ph), 133.63 (C-Ph), 132.84 (C-7), 126.71 (C-Ph), 124.210 (C-6), 123.26 (CH-triazole), 123.04 (C-5), 119.78 (C-Ph), 116.43 (C-8), 115.02 (C-4a), 91.46 (C-3), 62.81 (CH_2_). Anal. calcd. for C19H15N5O4 (Mr = 377.36): C 60.48, H 4.01, N 18.56; found: C 60.39, H 4.01, N 18.59%.

#### 3.6.9. 7-((1-(4-((*Z*)-*N*′-Hydroxycarbamimidoyl)phenyl)-1*H*-1,2,3-triazol-4-yl)methoxy)coumarin (**22**)

Compound **22** was prepared according to general procedure using **3i** (100 mg; 0.29 mmol), Et_3_N (0.12 mL) and NH_2_OH⸱HCl (60 mg) in solvent mixture methanol: DMF = 2:1 (9 mL). Compound **22** was isolated as white powder (68 mg; 62%; m.p. = 232–235 °C). ^1^H NMR (300 MHz, DMSO-*d*_6_) (*δ*/ppm): 9.81 (s, 1H, OH), 9.03 (s, 1H, H-triazole), 8.01 (d, *J =* 9.5 Hz, 1H, H-4), 7.91 (q, *J =* 9.0 Hz, 4H, Ph), 7.67 (d, *J =* 8.7 Hz, 1H, H-5), 7.21 (d, *J =* 2.4 Hz, 1H, H-8), 7.07 (dd, *J =* 8.6, 2.4 Hz, 1H, H-6), 6.32 (d, *J =* 9.5 Hz, 1H, H-3), 5.95 (s, 2H, NH_2_), 5.37 (s, 3H, CH_2_). ^13^C NMR (151 MHz, DMSO-*d*_6_) (*δ*/ppm): 161.04 (C-2), 160.25 (C-7), 155.31 (C-8a), 149.86 (C(NH_2_)=NOH), 144.29 (C-4), 143.24 (C-Ph), 136.60 (Cq-triazole), 133.59 (C-Ph), 129.58 (C-5), 126.76 (C-Ph), 123.09 (CH-triazole), 119.77 (C-Ph), 112.92 (C-3), 112.78 (C-6), 112.72 (C-4a), 101.66 (C-8), 61.62 (CH_2_). Anal. calcd. for C19H15N5O4 (Mr = 377.36): C 60.48, H 4.01, N 18.56; found: C 60.54, H 4.01, N 18.50%.

### 3.7. Cell Culturing

The cell lines HeLa (cervical carcinoma), SW620 (colorectal adenocarcinoma, metastatic), A549 (lung adenocarcinoma), HepG2 (hepatocellular carcinoma) and HFF (human foreskin fibroblasts) were cultured as monolayers and maintained in Dulbecco’s modified Eagle medium (DMEM), supplemented with 10% fetal bovine serum (FBS), 2mM L-glutamine, 100 U/mL penicillin and 100 μg/mL streptomycin in a humidified atmosphere with 5% CO_2_ at 37 °C.

### 3.8. Proliferation Assays

Cells were seeded onto 96-well microtiter plates at a seeding density of 3000 cells/well for carcinoma cell lines, and 5000 cells/well for normal human fibroblasts. The next day, cells were treated with test agents at five different concentrations (0.01–100 µM) and further incubated for 72 h. DMSO (solvent) was tested for potential cytotoxic effect but it did not exceed 0.1%. Following 72 h incubation, the MTT assay was performed and measured absorbances were transformed into percentage of cell growth as described previously [48]. Results were obtained from three independent experiments. IC_50_ values were calculated using linear regression analysis and results were statistically analyzed by ANOVA, Tukey post hoc test (*p* < 0.05).

### 3.9. DNA Binding Study

Compound solutions were used for measurements in aqueous buffer (pH = 7, sodium cacodylate buffer, *I* = 0.05 mol dm^−3^). Polynucleotides were purchased as noted: poly A-poly U, poly (dAdT)_2_ and calf thymus (ct)DNA (Sigma-Aldrich). Polynucleotides were dissolved in Na-cacodylate buffer, *I* = 0.05 mol dm^−3^, pH = 7. The calf thymus ctDNA was additionally sonicated and filtered through a 0.45 mm filter [49]. Polynucleotide concentration was determined spectroscopically [50,51] as the concentration of phosphates. 

#### 3.9.1. UV–Vis Measurements

UV–Vis spectra were recorded on a Varian Cary 100 Bio spectrophotometer using 1cm path quartz cuvettes. Calibration experiments were performed at 25 °C and pH = 7 (*I* = 0.05 mol dm^−3^, sodium cacodylate buffer). Thermal melting curves for DNA and their complexes with studied compounds were determined as previously described by following the absorption change at 260 nm as a function of temperature. The absorbance of the ligands was subtracted from every curve and the absorbance scale was normalized. *T*_m_ values are the midpoints of the transition curves determined from the maximum of the first derivative and checked graphically by the tangent method. The Δ*T*_m_ values were calculated by subtracting the *T*_m_ of the free nucleic acid from the *T*_m_ of the complex. Every Δ*T*_m_ value reported here was the average of at least two measurements. The error in Δ*T*_m_ is ±0.5 °C. 

#### 3.9.2. Fluorometric Measurements

Fluorescence spectra were recorded on a Varian Cary Eclipse spectrophotometer at 25 °C using appropriate 1cm path quartz cuvettes. Fluorometric experiments were performed at pH = 7 (*I* = 0.05 mol dm^−3^, sodium cacodylate buffer) by adding portions of polynucleotide solution into the solution of the studied compound. In fluorometric experiments, an excitation wavelength of λ_exc_ ≥ 300 nm was used to avoid the inner filter effect caused due to increasing absorbance of the polynucleotide. Emissions were determined in the range λ_em_ = 300–600 nm. Values for *K*_s_ obtained by processing titration data using the Scatchard equation [40], in most titrations have satisfactory correlation coefficients (≥0.98).

#### 3.9.3. CD Measurements

CD spectra were recorded on a JASCO J815 spectrophotometer in 1cm path quartz cuvettes. CD parameters: range = 500–220 nm, data pitch = 2, standard sensitivity, scanning speed = 200 nm/min, accumulation = 3–5. CD experiments were performed at 25 °C and pH = 7 (*I* = 0.05 mol dm^−3^, sodium cacodylate buffer) by adding portions of compound stock solution into the polynucleotide solution. 

## 4. Conclusions

The novel 1,2,3-triazolyl-appended indole, quinoline and coumarin heterocycles containing aryl imidazoline **4**–**11** and amidoxime **12**–**22** moiety substituted at the various position of heterocycle or connected via nitrogen- and oxygen-containing linkers were synthesized with the aim to assess the influence of performed structural modification on antiproliferative activity.

Antiproliferative evaluations on hepatocellular carcinoma (HepG2), showed that asymmetrical aromatic diamidine **5** and coumarin amidine **11** were the most potent compounds, with IC_50_ values in the nM range. However, **5** was also highly cytotoxic to normal HFF fibroblasts, while **11** exhibited less toxicity to non-tumor cells with a selectivity index (SI) of 11. Quinoline amidine **8** with -NCH_2_- spacer exhibited marked and selective effects against HepG2. Its analogue **9** with -OCH_2_- unit significantly inhibited HFF fibroblasts. From the amidoxime series, only quinoline amidoximes **18** and **20** showed a strong antiproliferative effect on A549, HeLa and SW620. Bis-amidoxime moiety in **20** contributed to its better activity compared to mono-amidoxime analogue **19**. Based on the results of CD titrations and thermal melting experiments, it can be concluded that **5** and **10** most likely bind inside the minor groove of AT-DNA and intercalate into AU-RNA. **6**, **9** and **11** bind to AT-DNA with mixed binding mode, most probably minor groove binding accompanied with aggregate binding along the DNA backbone.

## Figures and Tables

**Table 1:** Antiproliferative activity of amidino- **4**–**11** and amidoxime-substituted **12**–**22** 1,2,3-triazole derivatives.

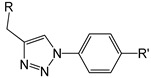 4–22							
Compd	R	R′	IC_50_^a^ (µM)				
			A549	HeLa	HepG2	SW620	HFF
**4**	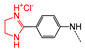	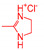	46.68	41.23	48.87	61.87	NA
**5**	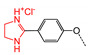	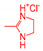	15.67	0.80	0.64	0.22	<0.01
**6**	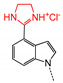	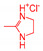	49.87	13.54	2.37	18.02	<0.01
**7**	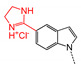	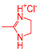	>100	>100	69.07	49.77	NA
**8**	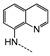	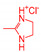	29.13	23.75	4.84	35.55	11.66
**9**	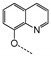	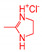	25.22	10.27	9.07	2.69	<0.01
**10**	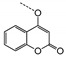	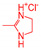	7.86	1.90	3.55	1.75	<0.01
**11**	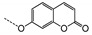	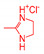	4.49	2.13	0.28	4.77	3.14
**12**	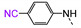	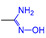	>100	>100	>100	65.14	NA
**13**	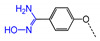	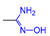	>100	>100	>100	>100	NA
**14**	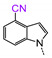	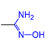	>100	65.75	>100	>100	NA
**15**	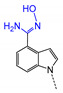	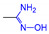	>100	>100	>100	>100	NA
**16**	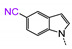	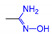	>100	>100	>100	>100	NA
**17**	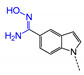	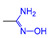	>100	60.66	>100	>100	NA
**18**	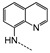	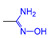	6.52	25.55	>100	NA	9.81
**19**	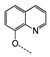	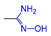	76.68	64.51	>100	72.71	NA
**20**	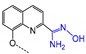	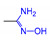	31.35	7.15	51.31	7.24	NA
**21**	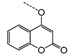	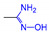	>100	99.72	>100	>100	NA
**22**	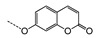	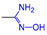	>100	87.38	91.40	>100	NA
**5-FU**			2.80	8.81	8.90	0.08	/

^a^ IC_50_; 50% inhibitory concentration, or compound concentration required to inhibit tumor cell proliferation by 50%.

**Table 2:** Binding constants (log*Ks*) ^a^ and ratios *n* ^b^ (bound compound/polynucleotide phosphate) calculated from the fluorescence titrations of **5**, **6**, **8**–**11** and **20** with ds-polynucleotides at pH = 7.0 (buffer sodium cacodylate, *I* = 0.05 mol dm^−3^).

	ctDNA			p(dAdT)_2_			poly A-poly U		
	log*K_s_*	*n*	I/I_0_^c^	log*K_s_*	*n*	I/I_0_^c^	log*K_s_*	*n*	I/I_0_^c^
**5**	5.7	0.1	2.0	6.5	<0.1	2.2	7.0	0.7	2.0
**6**	5.0	0.7	0.8	6.6	0.1	0.9	4.2	0.6	0.1
**8**	- ^d^	- ^d^	- ^d^	5.5	0.5	2.3	- ^d^	- ^d^	- ^d^
**9**	5.3	0.3	0.5	5.8	0.1	0.2	5.1	<0.1	<0.1
**10**	- ^d^	- ^d^	- ^d^	6.7	0.8	1.3	- ^d^	- ^d^	- ^d^
**11**	4.5 ^d^	0.2	0.5	4.9	0.2	0.6	4.9	0.2	0.7
**20**	4.3 ^d^	0.2	-	4.7 ^d^	0.1	-	4.6 ^d^	0.1	-

^a,b^ Processing of titration data using Scatchard equation [40] gave values of ratio *n* (bound compound/polynucleotide); correlation coefficients were >0.98 for most of calculated *K*_a_. ^c^ *I*_0_—starting fluorescence intensity of compound; *I*—fluorescence intensity of compound/polynucleotide complex calculated by Scatchard equation. ^d^ small fluorescence change disabled calculation of stability constant.

## Data Availability

Data is contained within the article and Supplementary Materials.

## Supplementary Materials

The following are available online. UV–VIS spectra, fluorescence spectra, ^1^H and ^13^C NMR spectra.
