# Supplementary material for: Amidine- and Amidoxime-Substituted Heterocycles: Synthesis, Antiproliferative Evaluations and DNA Binding
Source: Molecules. 2021 Nov 22;26(22):7060. doi: 10.3390/molecules26227060 (PMC8625065; doi:10.3390/molecules26227060)

Amidine- and amidoxime-substituted heterocycles: synthesis, antiproliferative evaluations and DNA binding

Silvija Maračić,[1] Petra Grbčić,[2] Suresh Shammugam,[3] Marijana Radić Stojković,[3*] Krešimir Pavelić,[4] Mirela Sedić,[5] Sandra Kraljević Pavelić[6] and Silvana Raić-Malić[1*]

[1]University of Zagreb, Faculty of Chemical Engineering and Technology, Department of Organic Chemistry, Marulićev trg 19, HR-10000 Zagreb, Croatia; skorunda@fkit.hr (SM); sraic@fkit.hr (SRM)

[2]University of Rijeka, Department of Biotechnology, Ulica Radmile Matejčić 2, HR-51000 Rijeka, Croatia; petra.grbcic@biotech.uniri.hr (PG)

[3] Division of Organic Chemistry and Biochemistry, Laboratory for Biomolecular Interactions and Spectroscopy, Ruđer Bošković Institute, Bijenička 54, HR-10000, Zagreb, Croatia; mradic@irb.hr (MRS); sshanmug@irb.hr (SS)

[4] Juraj Dobrila University of Pula, Pula, Croatia, e-mail: pavelic@unipu.hr (KP)

[5] Centre for Applied Bioanthropolog, Institute for Anthropological Research, Ljudevita Gaja 32, HR-10000, Zagreb, Croatia; mirela.sedic@inantro.hr (MS)

[6] University of Rijeka, Faculty of Health Studies, Ulica Viktora Cara Emina 5, HR-51000 Rijeka, Croatia, sandrakp@uniri.hr (SKP)

*Corresponding authors

Marijana Radić Stojković, Division of Organic Chemistry and Biochemistry, Laboratory for Biomolecular Interactions and Spectroscopy, Ruđer Bošković Institute, Bijenička 54, HR-10000, Zagreb, Croatia, email: mradic@irb.hr

Silvana Raić-Malić, University of Zagreb, Department of Organic Chemistry, Faculty of Chemical Engineering and Technology, Marulićev trg 19, HR-10000 Zagreb, Croatia, email: sraic@fkit.hr

Contents

1. Spectroscopic characterization of studied compounds in aqueous solutions S1
2. Interactions of studied compounds with ds-polynucleotides in neutral medium (pH=7.0) S14
   2.1. Fluorimetric titrations S14
   2.2. Thermal melting experiments S24
   2.3. Circular dichroism (CD) titrations S28
3. ^{1}H and ^{13}C spectra of compounds **4**–**22** S30

1. Spectroscopic characterization of studied compounds in aqueous solutions

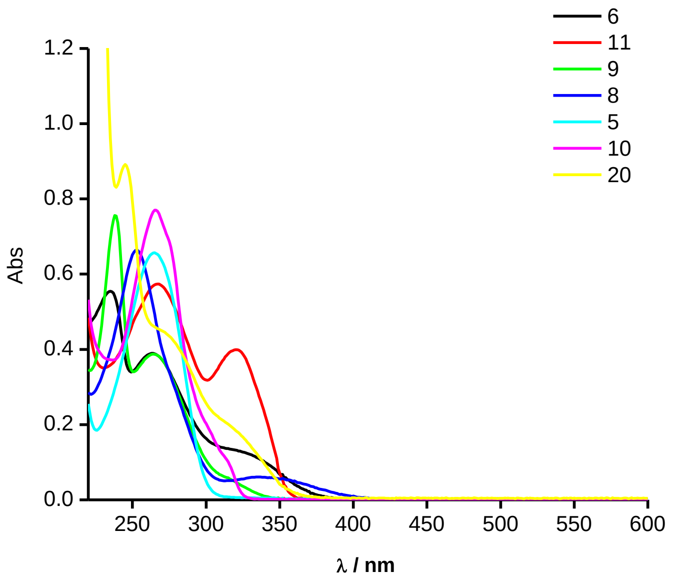

Figure S1. UV/Vis spectra of **5, 6, 8-11** and **20** at c = 2 × 10^{-5} mol dm^{-3}; pH=7, sodium cacodylate/HCl buffer, I = 0.05 mol dm^{-3}.

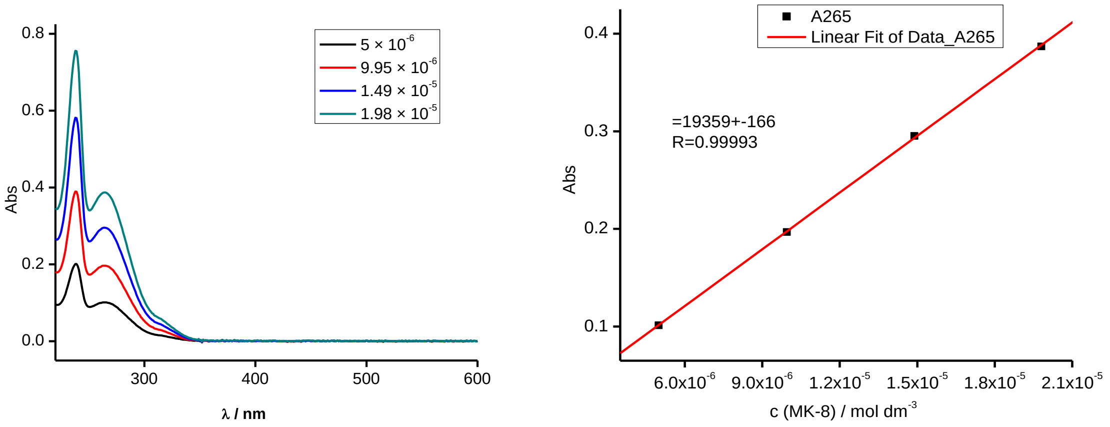

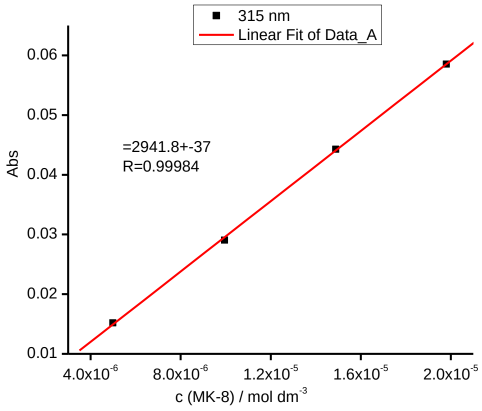

Figure S2. UV/Vis spectra changes of **9** at different concentrations (concentration range from 5×10^{-6} to 1.98×10^{-5} mol dm^{-3}) at pH=7, sodium cacodylate buffer, *I*=0.05 M.

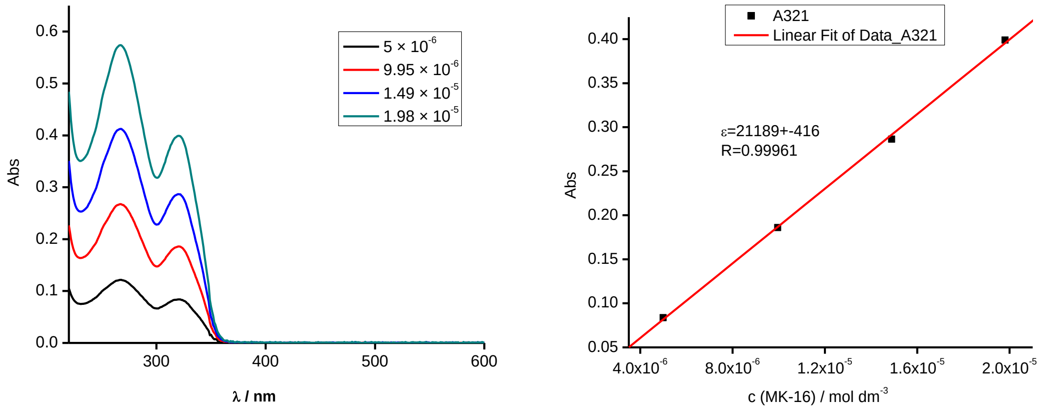

Figure S3. UV/Vis spectra changes of **11** at different concentrations (concentration range from 5×10^{-6} to 1.98×10^{-5} mol dm^{-3}) at pH=7, sodium cacodylate buffer, *I*=0.05 M.

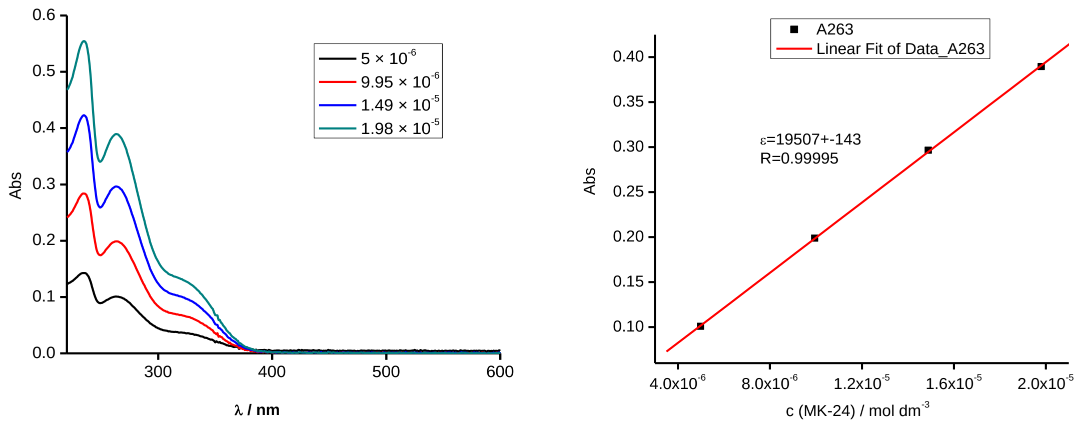

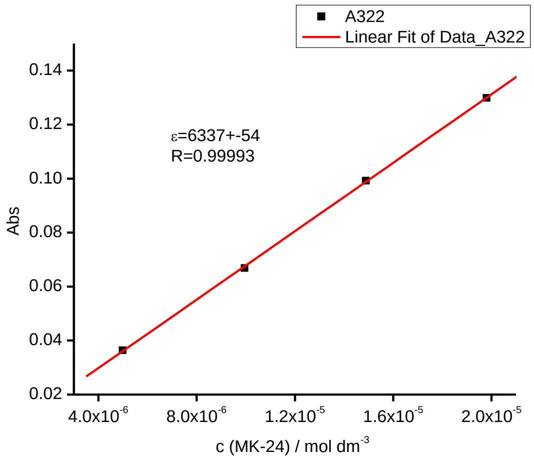

Figure S4. UV/Vis spectra changes of **6** at different concentrations (concentration range from 5×10^{-6} to 1.98×10^{-5} mol dm^{-3}) at pH=7, sodium cacodylate buffer, *I*=0.05 M

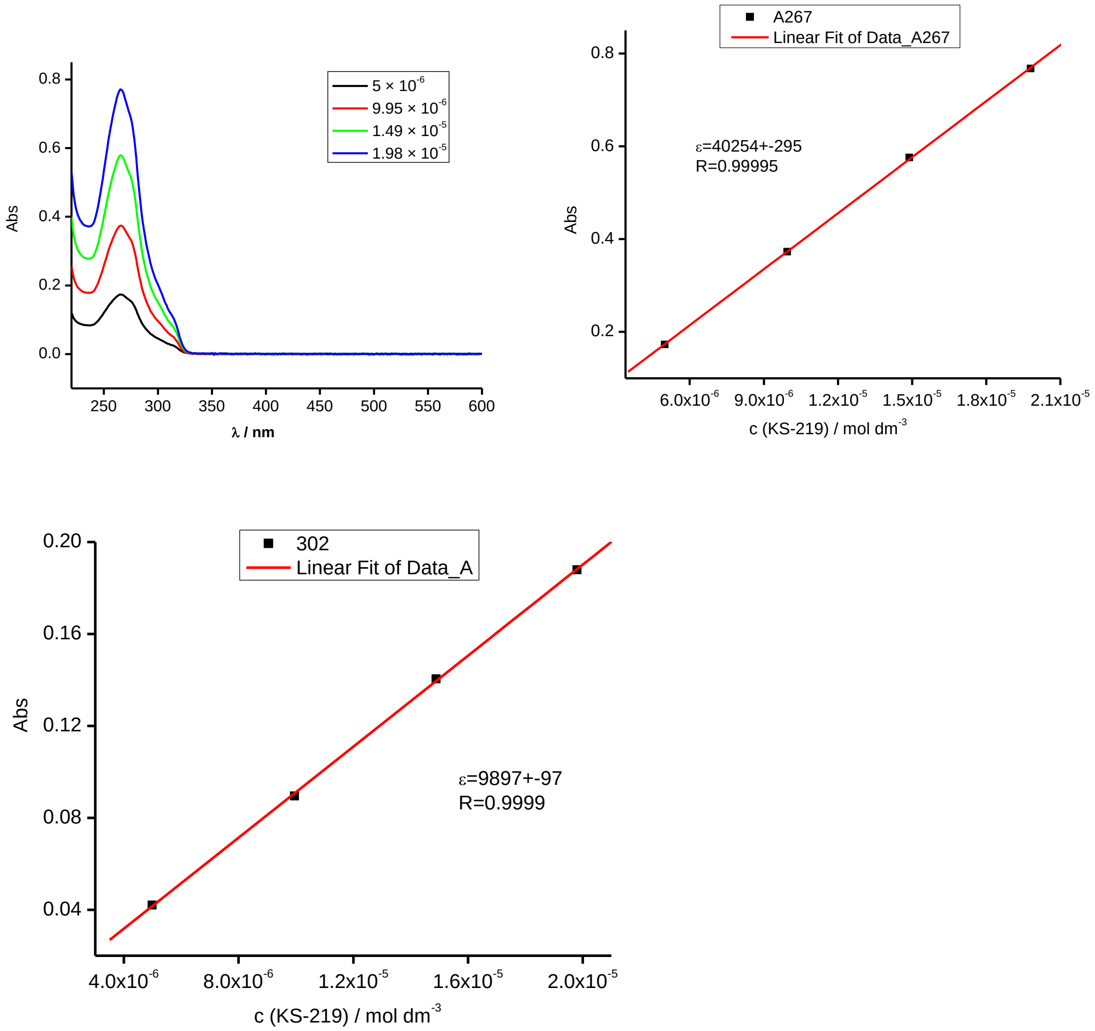

Figure S5. UV/Vis spectra changes of **10** at different concentrations (concentration range from 5×10^{-6} to 1.98×10^{-5} mol dm^{-3}) at pH=7, sodium cacodylate buffer, *I*=0.05 M

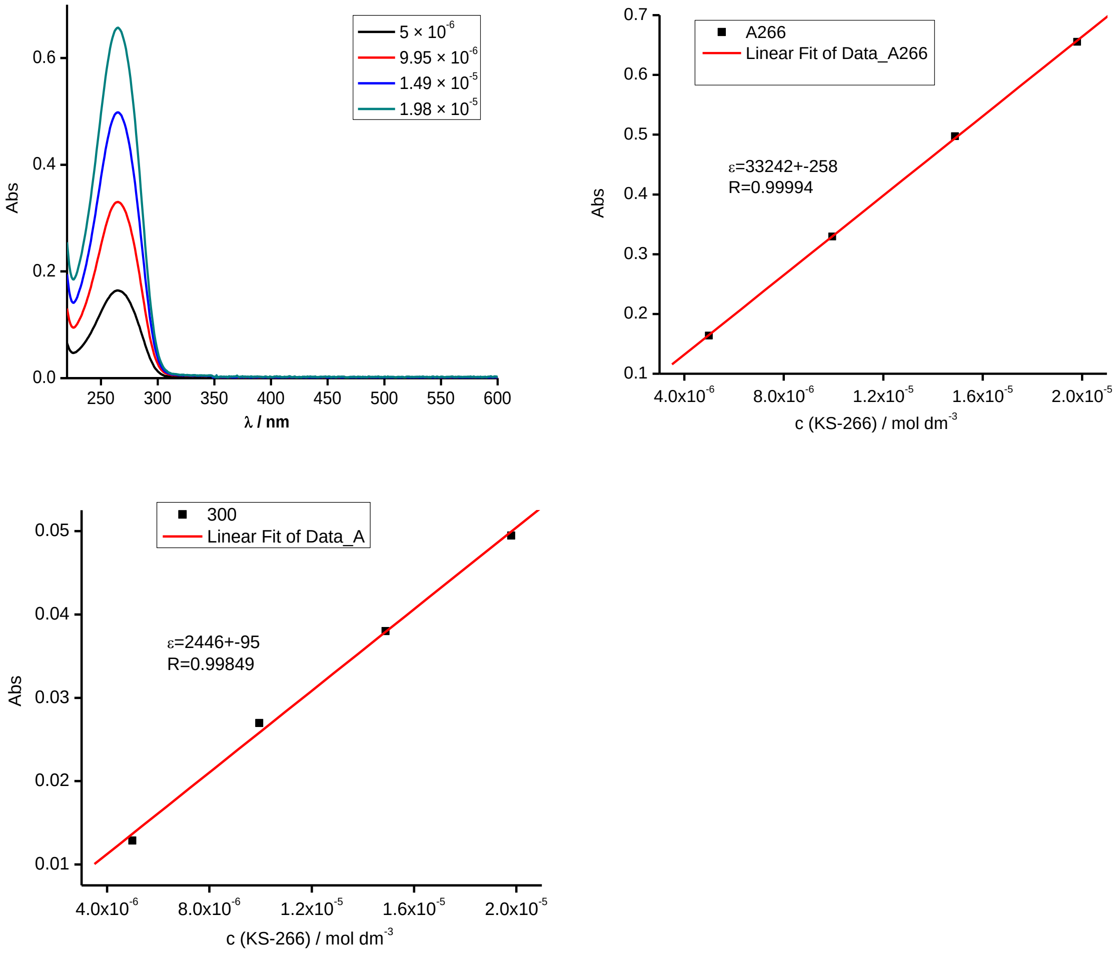

Figure S6. UV/Vis spectra changes of **5** at different concentrations (concentration range from 5×10^{-6} to 1.98×10^{-5} mol dm^{-3}) at pH=7, sodium cacodylate buffer, I=0.05 M

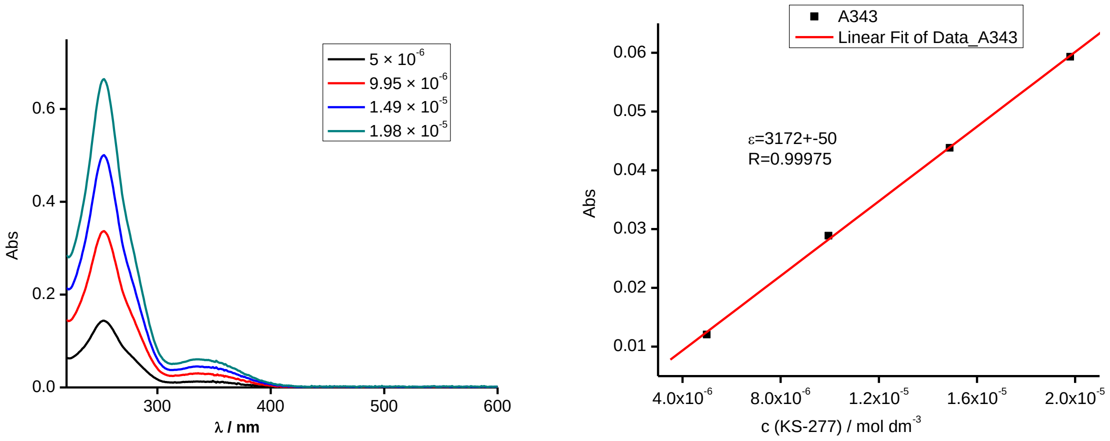

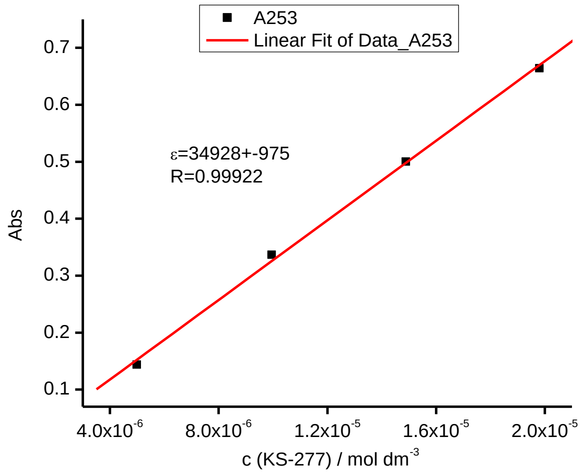

Figure S7. UV/Vis spectra changes of **8** at different concentrations (concentration range from 5×10^{-6} to 1.98×10^{-5} mol dm^{-3}) at pH=7, sodium cacodylate buffer, *I*=0.05 M

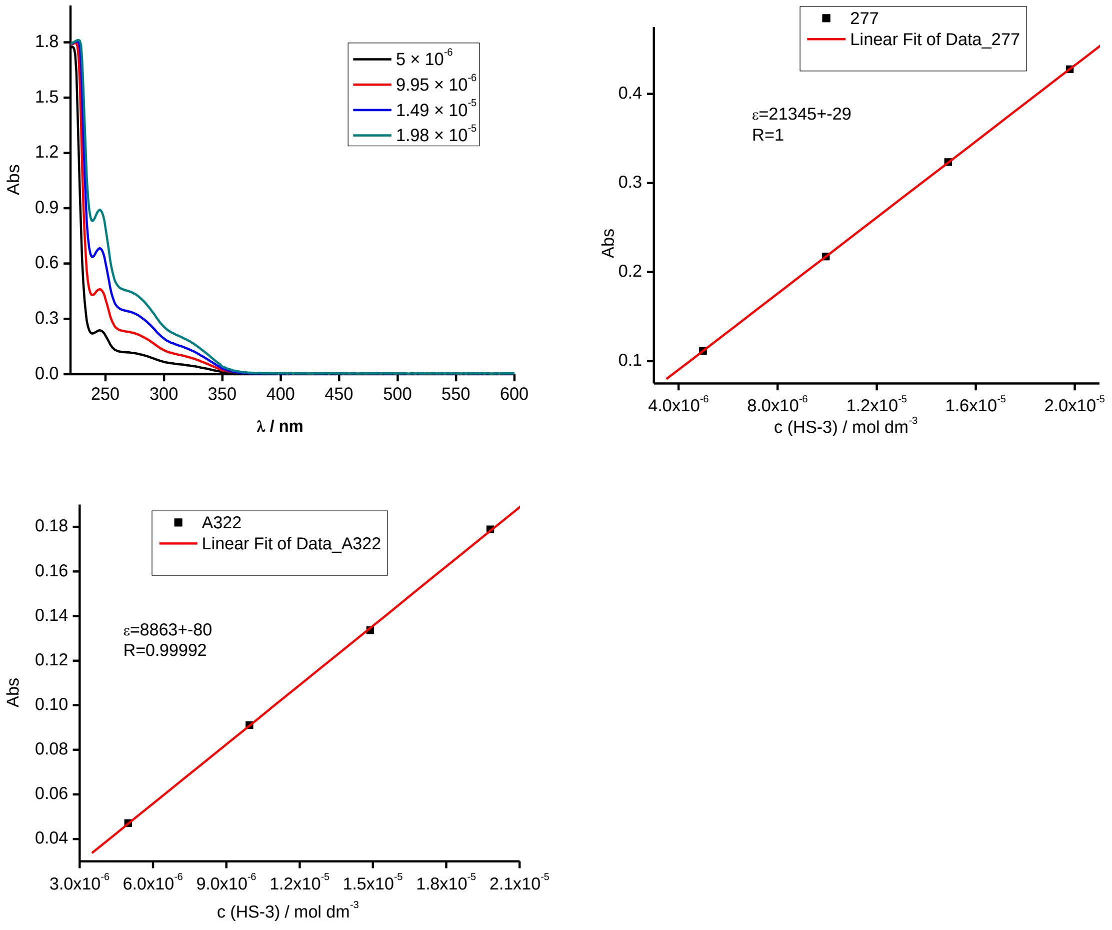

Figure S8. UV/Vis spectra changes of **20** at different concentrations (concentration range from 5×10^{-6} to 1.98×10^{-5} mol dm^{-3}) at pH=7, sodium cacodylate buffer, *I*=0.05 M

Table S1. Electronic absorption data of **5, 6, 8-11** and **20.**

	pH = 7,0[a]	
	λ_{max} / nm	$\varepsilon \times 10^3$ / $mmol^{-1}$ cm^2
**9**	265	19.3
	315	24.9
**11**	321	21.3
**6**	263	19.5
	322	63.3
**10**	267	40.2
	302	98.9
**5**	266	33.2
	300	24.4
**8**	253	34.9
	343	31.7
**20**	277	21.3
	322	88.6

[a] Sodium cacodylate buffer, I = 0,05 mol dm^{-3}, pH = 7,0.

The emission intensities of buffered aqueous solutions (sodium cacodylate buffer, I = 0.05 mol dm^{-3}, pH = 7) of studied compounds are proportional to their concentrations up to $c = 2 \times 10^{-6}$ mol dm^{-3} (Figures S9-16).

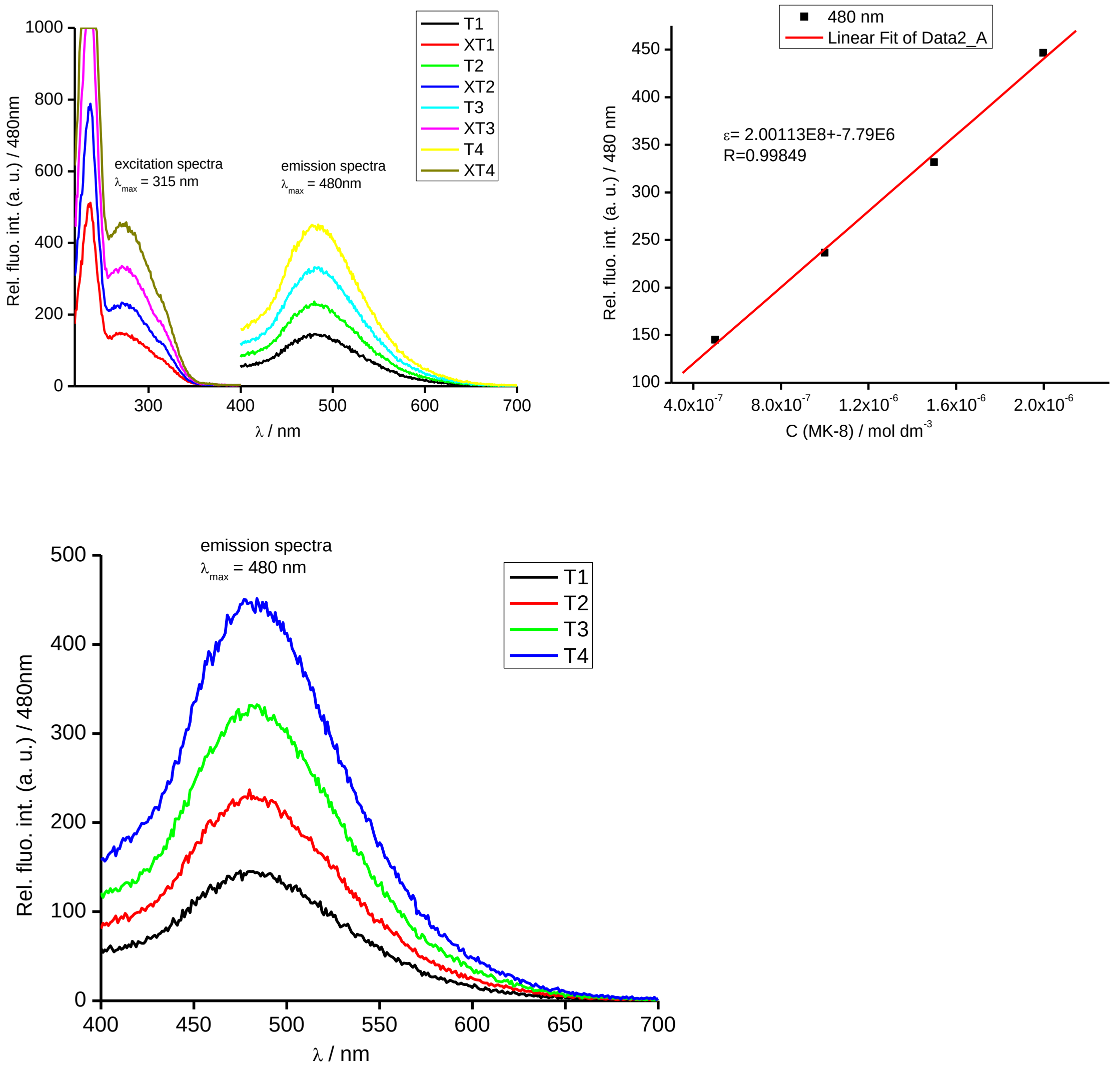

Figure S9. Emission and excitation spectra changes **9** at different concentrations at λ_{exc}=315 nm and λ_{em}= 480 nm (concentration range from 4.99 × 10^{-7} to 1.99 × 10^{-6} mol dm^{-3}) at pH=7, Na cacodylate buffer, I=0.05 mol dm^{-3}.

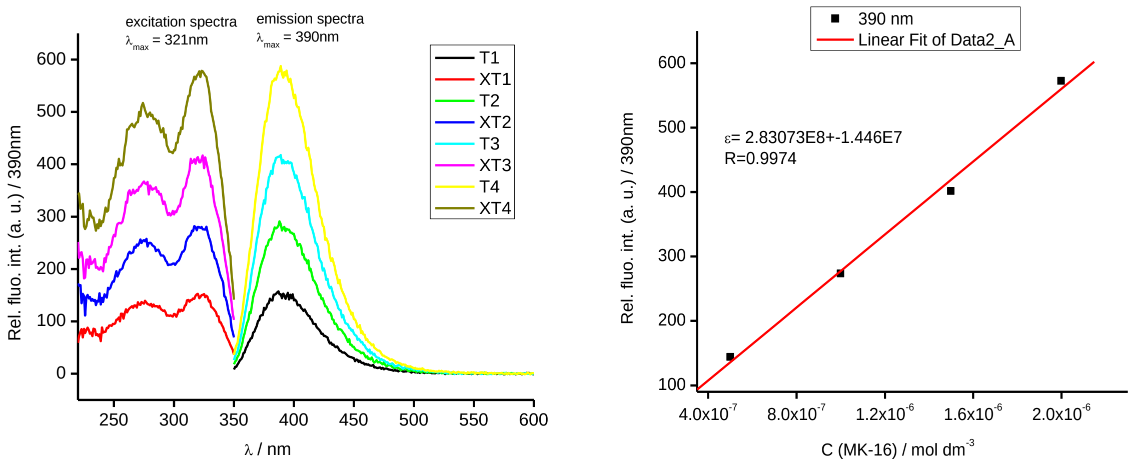

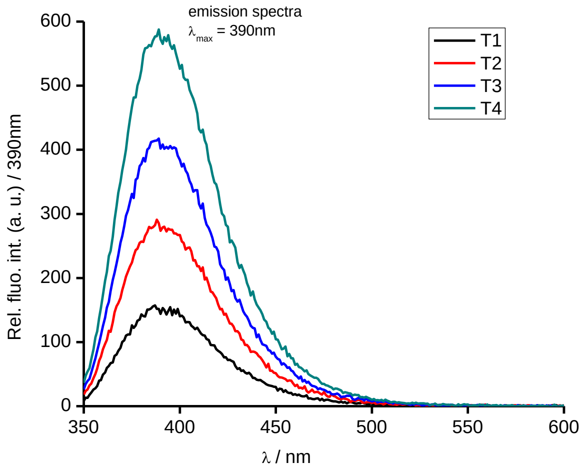

Figure S10. Emission and excitation spectra changes of **11** at different concentrations at λ_{exc}=321nm and λ_{em}=390nm (concentration range from 4.99 × 10^{-7} to 1.99 × 10^{-6} mol dm^{-3}) at pH=7, Na cacodylate buffer, *I*=0.05 mol dm^{-3}.

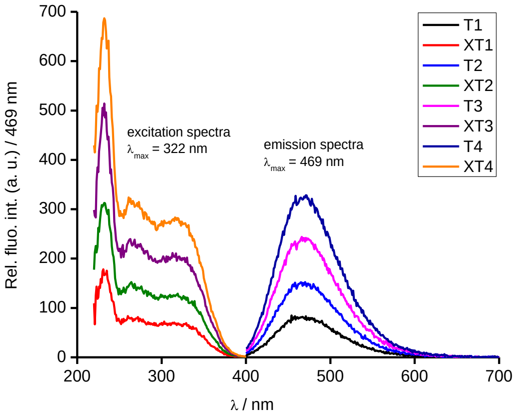

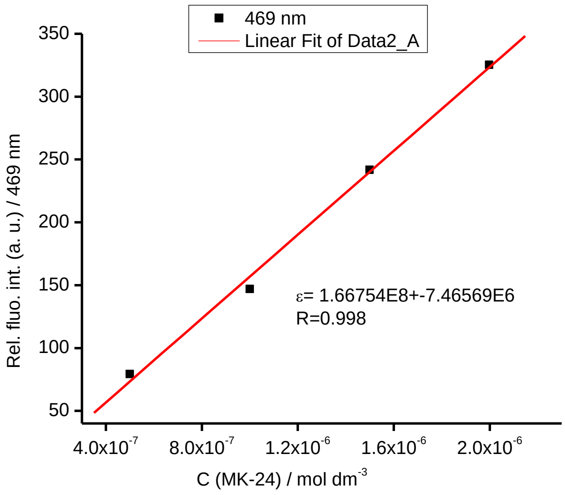

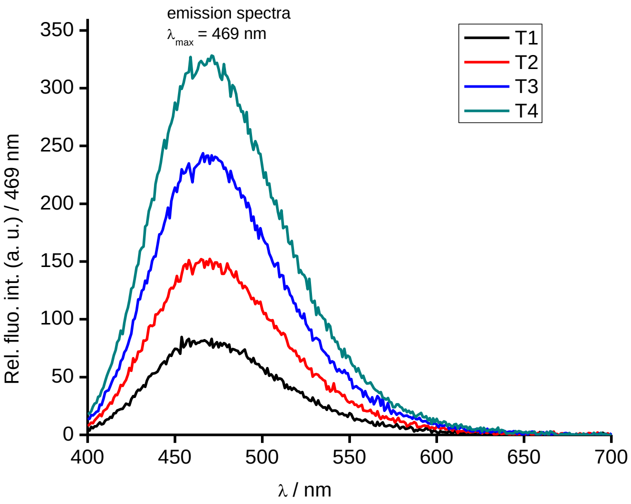

Figure S11. Emission and excitation spectra changes of **6** at different concentrations at λ_{exc}=322nm and λ_{em}=469 nm (concentration range from 4.99 × 10^{-7} to 1.99 × 10^{-6} mol dm^{-3}) at pH=7, Na cacodylate buffer, I=0.05 mol dm^{-3}.

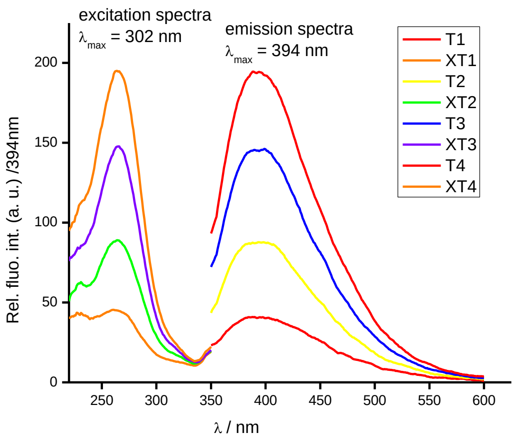

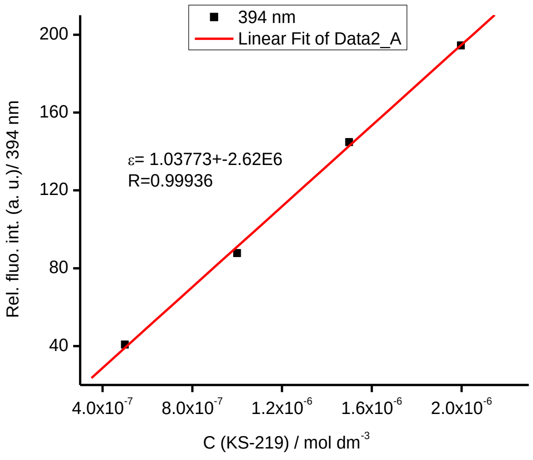

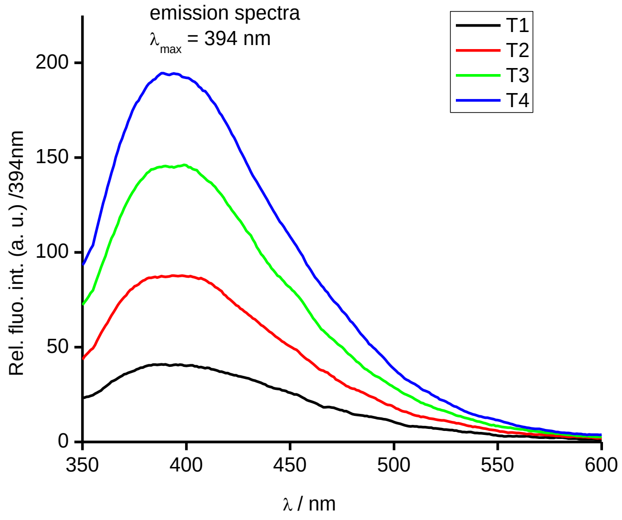

Figure S12. Emission and excitation spectra changes of **10** at different concentrations at λ_{exc}=302 and λ_{em}=394 nm nm (concentration range from 4.99 × 10^{-7} to 1.99 × 10^{-6} mol dm^{-3}) at pH=7, Na cacodylate buffer, *I*=0.05 mol dm^{-3}.

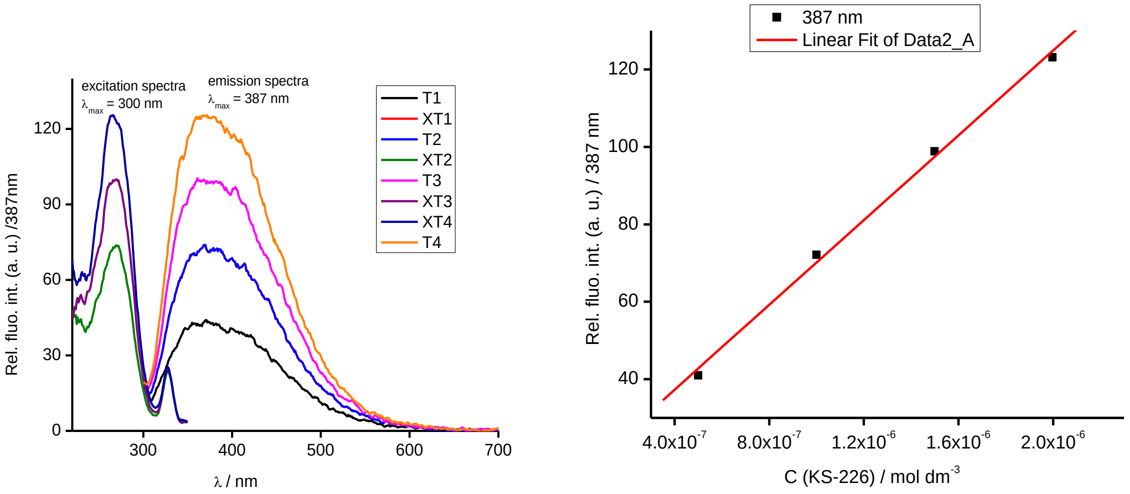

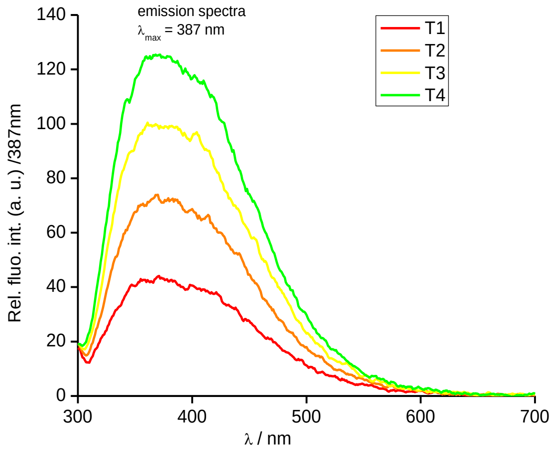

Figure S13. Emission and excitation spectra changes of **5** at different concentrations at λ_{exc}=300 nm and λ_{exc}=387 nm (concentration range from 4.99 × 10^{-7} to 1.99 × 10^{-6} mol dm^{-3}) at pH=7, Na cacodylate buffer, I=0.05 mol dm^{-3}.

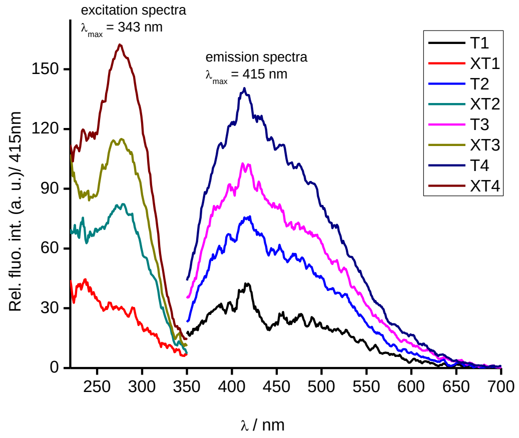

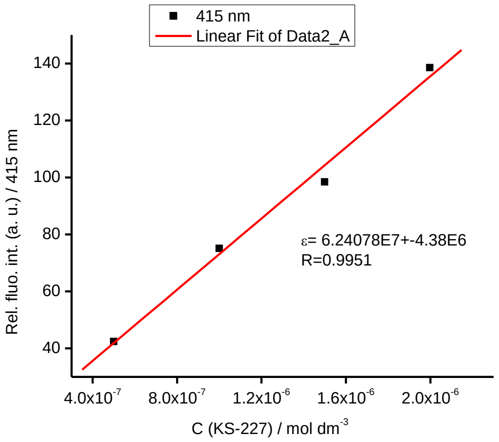

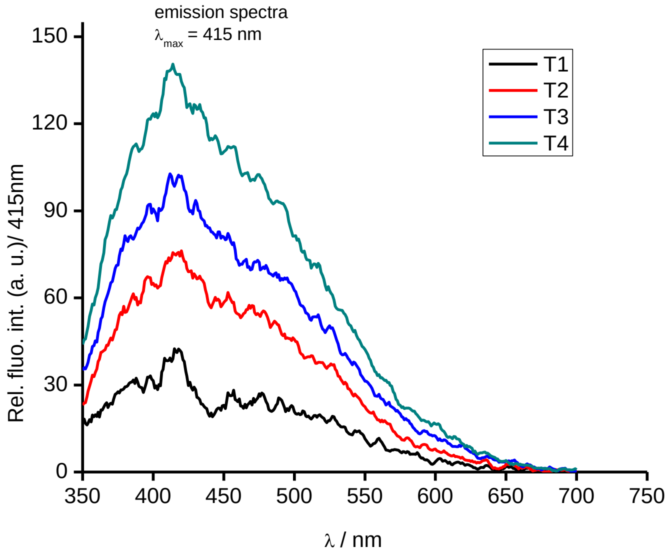

Figure S14. Emission and excitation spectra changes of **8** at different concentrations at λ_{exc}=343 nm and λ_{exc}=415 nm (concentration range from 4.99 × 10^{-7} to 1.99 × 10^{-6} mol dm^{-3}) at pH=7, Na cacodylate buffer, I=0.05 mol dm^{-3}.

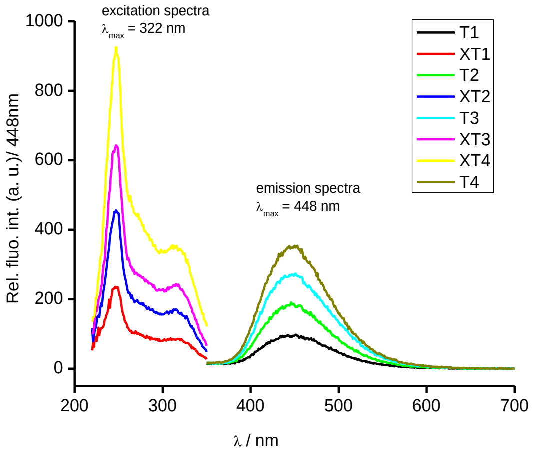

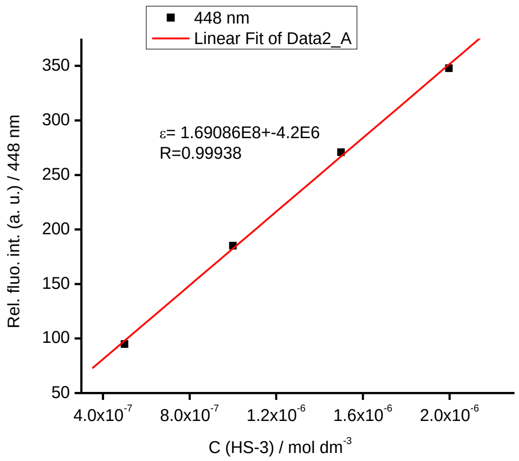

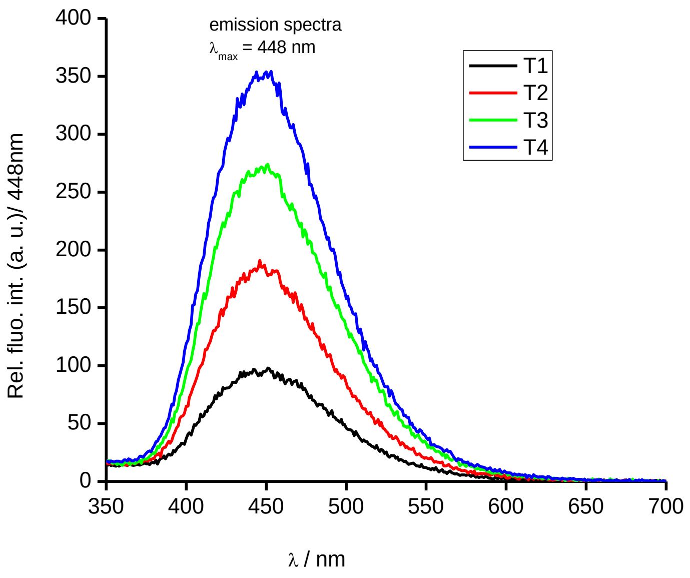

Figure S15. Emission and excitation spectra changes of **20** at different concentrations at λ_{exc}=322nm and λ_{exc}=448nm (concentration range from 4.99 × 10^{-7} to 1.99 × 10^{-6} mol dm^{-3}) at pH=7, Na cacodylate buffer, *I*=0.05 mol dm^{-3}.

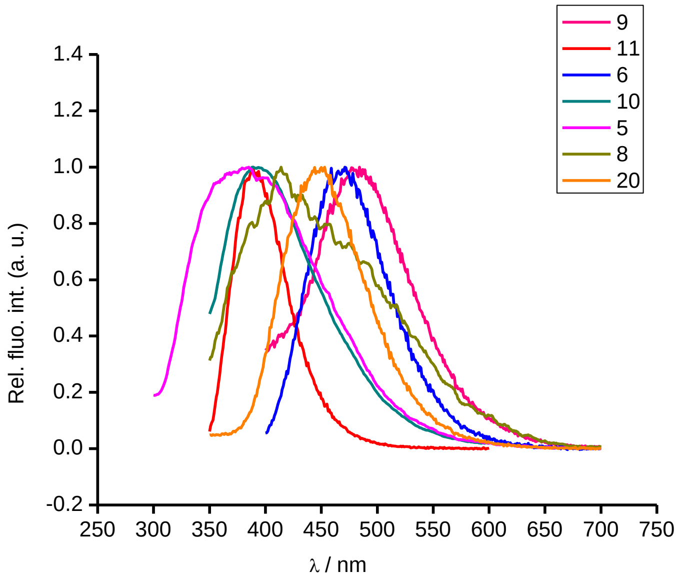

Figure S16. Normalized emission spectra of **9** (λ_{exc}=315nm), **11** (λ_{exc}=321nm), **6** (λ_{exc}=322nm), **10** (λ_{exc}=302nm), **5** (λ_{exc}=300nm), **8** (λ_{exc}=343nm) and **20** (λ_{exc}=322nm) at concentration, c=2 × 10^{-6} mol dm^{-3} at pH=7, Na cacodylate buffer, *I*=0.05 mol dm^{-3}.

2. Interactions of studied compounds with ds-polynucleotides in neutral medium (pH=7.0)

2.1. Fluorimetric titrations

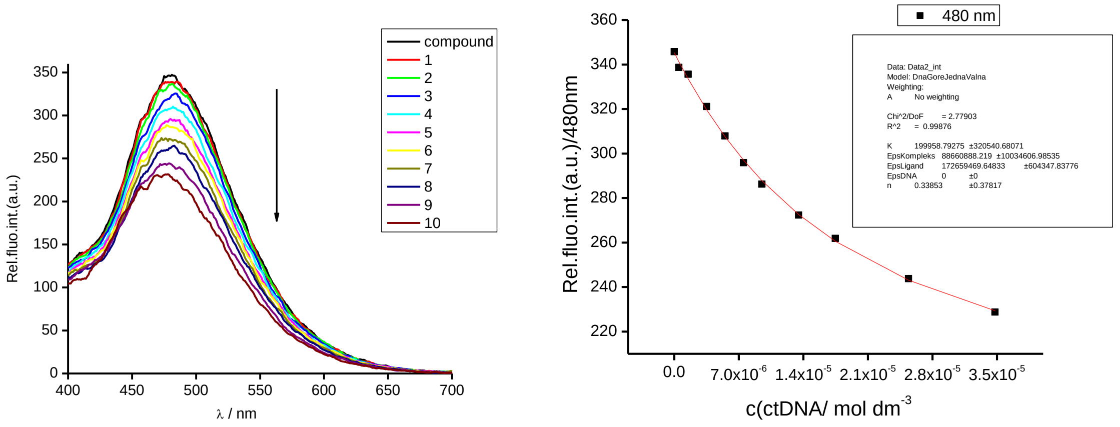

Figure S17. a) Changes in fluorescence spectrum of **9** (c= 2.0 × 10^{-6} mol dm^{-3}, λ_{exc}=315 nm) upon titration with ctDNA (c= 5.02 × 10^{-7}– 4.7 × 10^{-5} mol dm^{-3}); b) Dependence of **9** absorbance at λ_{max} = 480 nm on c(ctDNA), at pH=7, sodium cacodylate buffer, I = 0.05 mol dm^{-3}.

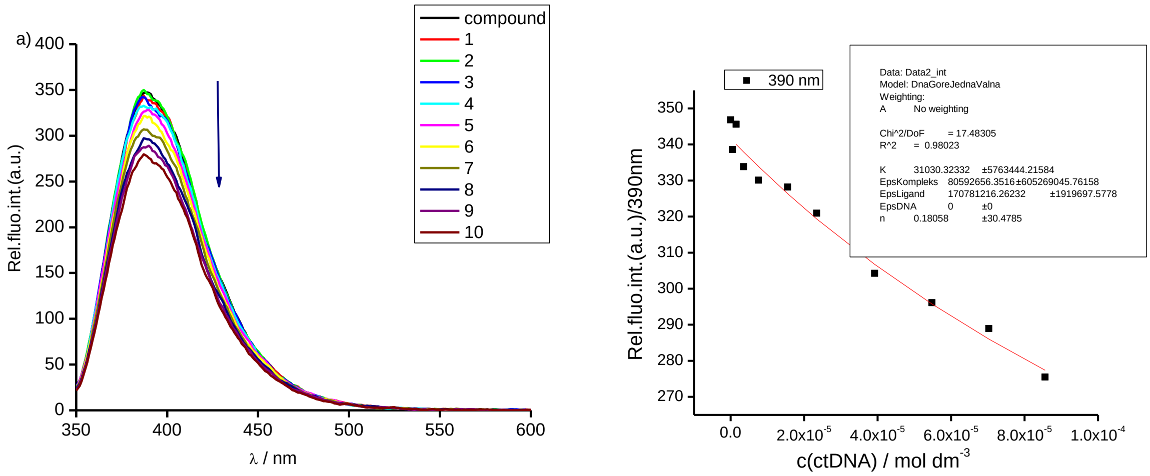

Figure S18. a) Changes in fluorescence spectrum of **11** (c= 2.0 × 10^{-6} mol dm^{-3}, λ_{exc}=321 nm) upon titration with ctDNA (c= 5.02 × 10^{-7}– 8.56× 10^{-5} mol dm^{-3}); b) Dependence of **11** absorbance at λ_{max} = 390 nm on c(ctDNA), at pH=7, sodium cacodylate buffer, I = 0.05 mol dm^{-3}.

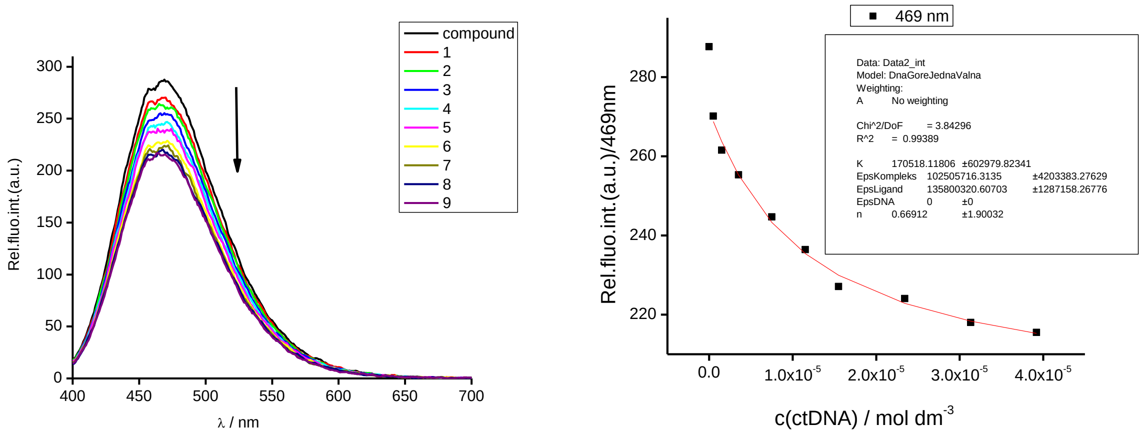

Figure S19. a) Changes in fluorescence spectrum of **6** (c= 2.0 × 10^{-6} mol dm^{-3}, λ_{exc}=322 nm) upon titration with ctDNA (c= 5.02 × 10^{-7}- 3.92× 10^{-5} mol dm^{-3}); b) Dependence of **6** absorbance at λ_{max} = 469 nm on c(ctDNA), at pH=7, sodium cacodylate buffer, I = 0.05 mol dm^{-3}.

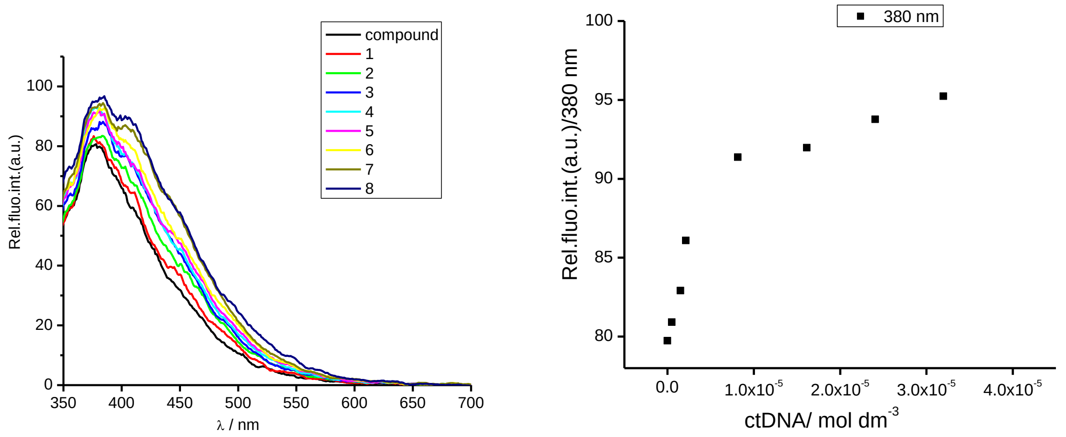

Figure S20. a) Changes in fluorescence spectrum of **10** (c= 2.0 × 10^{-6} mol dm^{-3}, λ_{exc}=302 nm) upon titration with ctDNA (c= 5.0 × 10^{-7} – 3.2× 10^{-5} mol dm^{-3}); b) Dependence of **10** absorbance at λ_{max}= 380nm on c(ctDNA), at pH=7, sodium cacodylate buffer, I = 0.05 mol dm^{-3}.

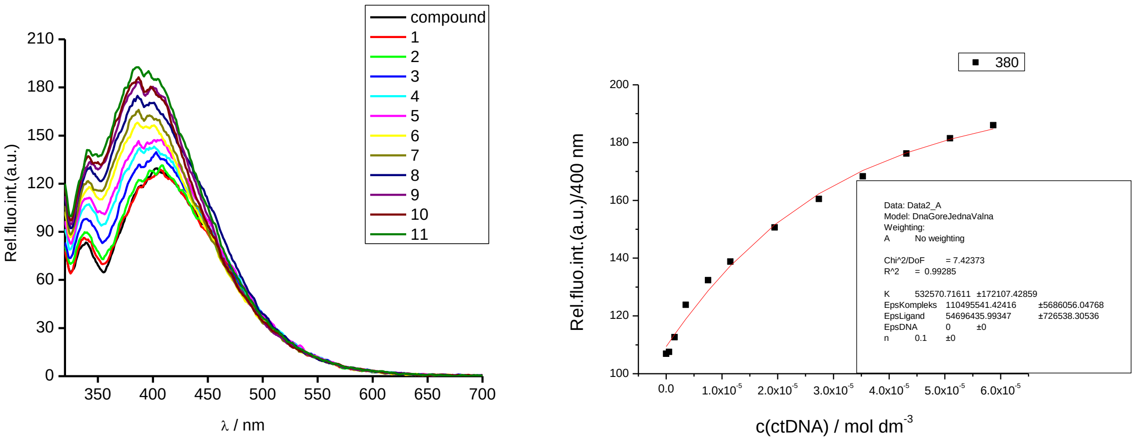

Figure S21. a) Changes in fluorescence spectrum of **5** (c= 2.0 × 10^{-6} mol dm^{-3}, λ_{exc}=300 nm) upon titration with ctDNA (c= 5.02 × 10^{-6} – 5.87× 10^{-5} mol dm^{-3}); b) Dependence of **5** absorbance at λ_{max} = 380 nm on c(ctDNA), at pH=7, sodium cacodylate buffer, I = 0.05 mol dm^{-3}.

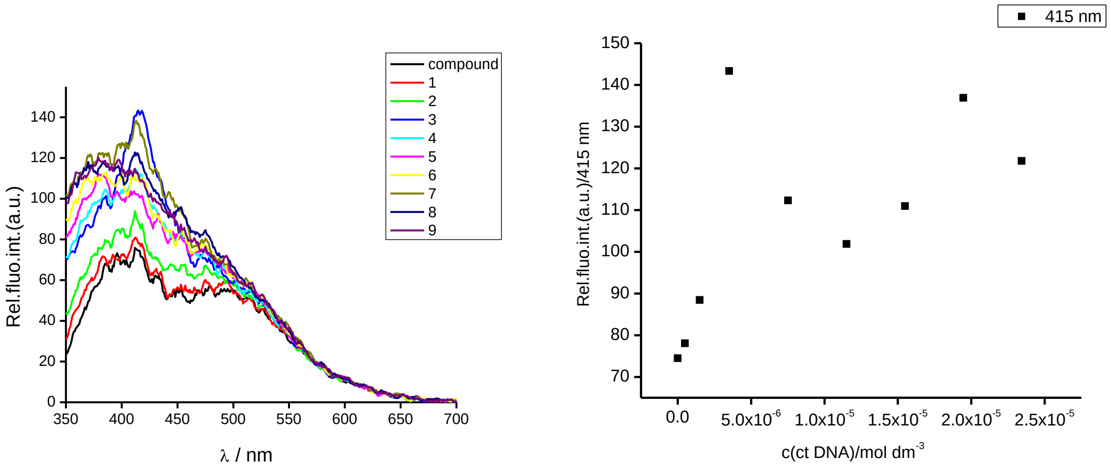

Figure S22. a) Changes in fluorescence spectrum of **8** (c= 2.0 × 10^{-6} mol dm^{-3}, λ_{exc}=343 nm) upon titration with ctDNA (c= 5.02 × 10^{-6} – 2.3× 10^{-5} mol dm^{-3}); b) Dependence of **8** absorbance at λ_{max} = 415 nm on c(ctDNA), at pH=7, sodium cacodylate buffer, I = 0.05 mol dm^{-3}.

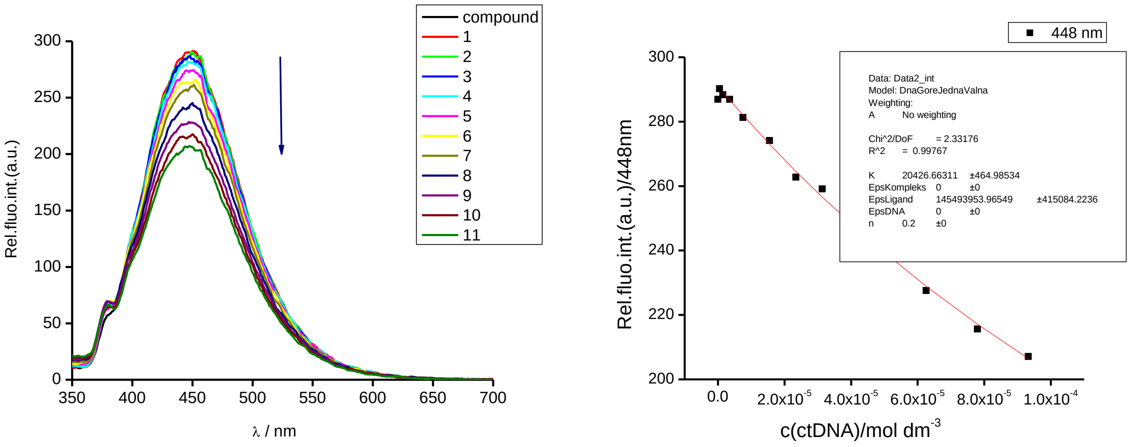

Figure S23. a) Changes in fluorescence spectrum of **20** (c= 2.0 × 10^{-6} mol dm^{-3}, λ_{exc}=322 nm) upon titration with ctDNA (c= 5.02 × 10^{-6}– 9.32× 10^{-5} mol dm^{-3}); b) Dependence of **20** absorbance at λ_{max} = 448 nm on c(ctDNA), at pH=7, sodium cacodylate buffer, I = 0.05 mol dm^{-3}.

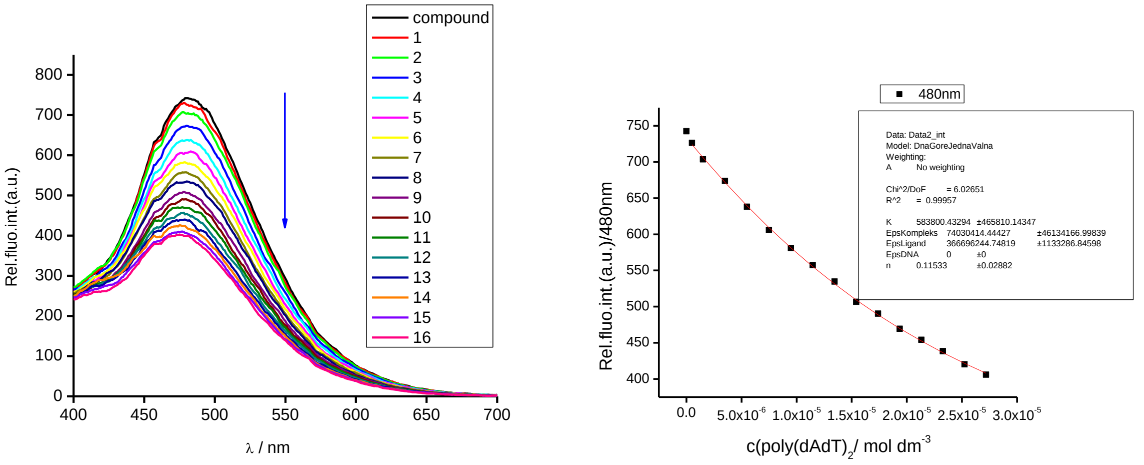

Figure S24. a) Changes in fluorescence spectrum of **9** (c= 2,0 × 10^{-6} mol dm^{-3}, λ_{exc}=315 nm) upon titration with poly(dAT)$_2$ (c= 5.01 × 10^{-7} – 2.72 × 10^{-5} mol dm^{-3}); b) Dependence of **9** absorbance at λ_{max}= 480 nm on c(poly(dA-dT)$_2$, at pH=7, sodium cacodylate buffer, I = 0.05 mol dm^{-3}.

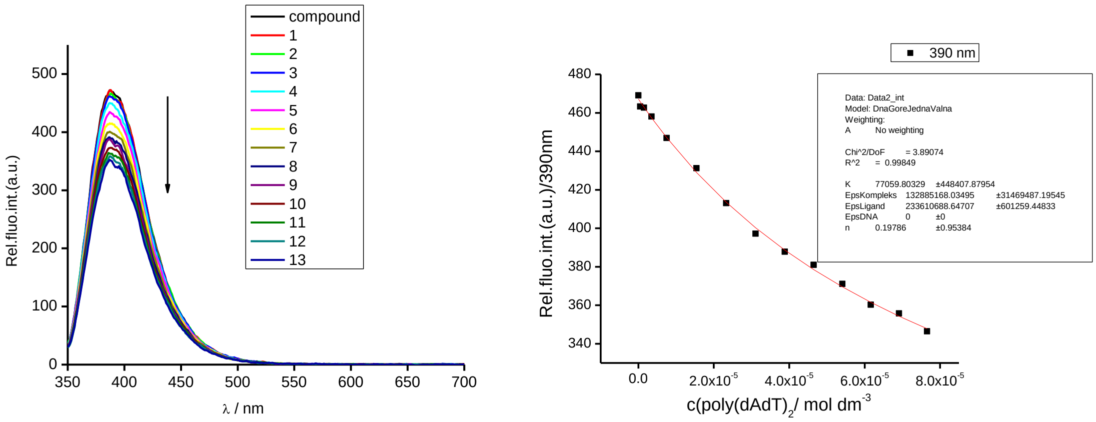

Figure S25. a) Changes in fluorescence spectrum of **11** (c= 2,0 × 10^{-6} mol dm^{-3}, λ_{exc}=321 nm) upon titration with poly $(dAdT)_2$ (c= 5.01 × 10^{-7} – 7.65× 10^{-5} mol dm^{-3}); b) Dependence of **11** absorbance at λ_{max}= 390 nm on c (poly $(dAdT)_2$), at pH=7, sodium cacodylate buffer, I = 0.05 mol dm^{-3}.

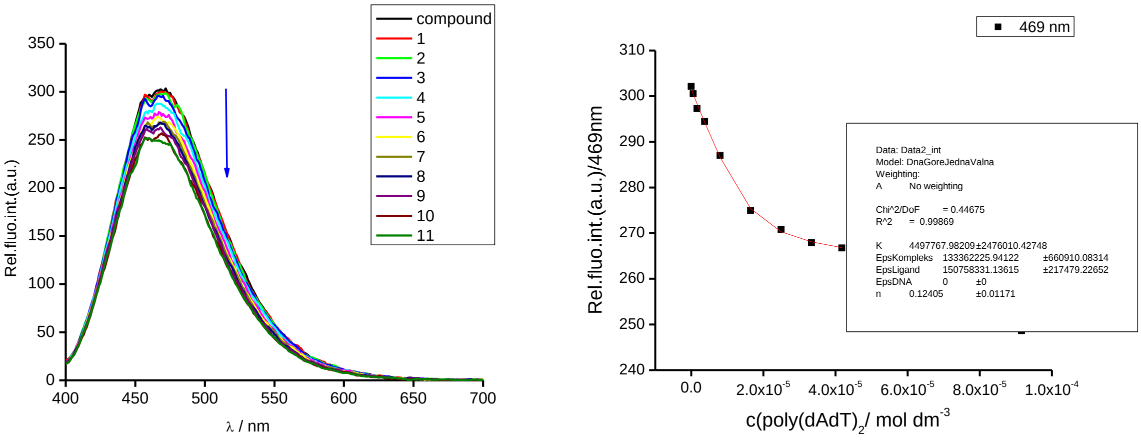

Figure S26. a) Changes in fluorescence spectrum of **6** (c= 2,0 × 10^{-6} mol dm^{-3}, λ_{exc}=322 nm) upon titration with poly $(dAdT)_2$ (c= 5.32 × 10^{-7} – 9.16× 10^{-5} mol dm^{-3}); b) Dependence of **6** absorbance at λ_{max}= 469 nm on c (poly $(dAdT)_2$), at pH=7, sodium cacodylate buffer, I = 0.05 mol dm^{-3}.

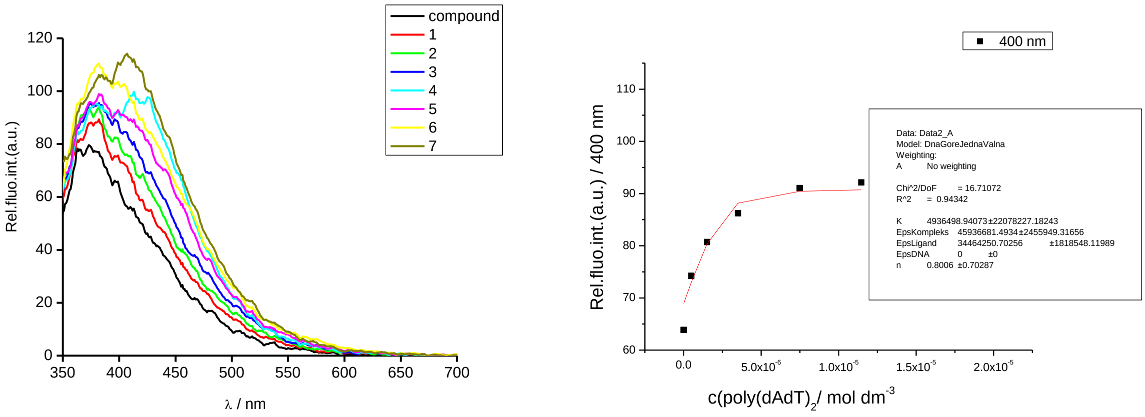

Figure S27. a) Changes in fluorescence spectrum of **10** (c= 2,0 × 10^{-6} mol dm^{-3}, λ_{exc}=302 nm) upon titration with poly $(dAdT)_2$ (c= 5.0 × 10^{-7} – 1.94× 10^{-5} mol dm^{-3}); b) Dependence of **10** absorbance at λ_{max}= 400 nm on c (poly $(dAdT)_2$), at pH=7, sodium cacodylate buffer, I = 0.05 mol dm^{-3}.

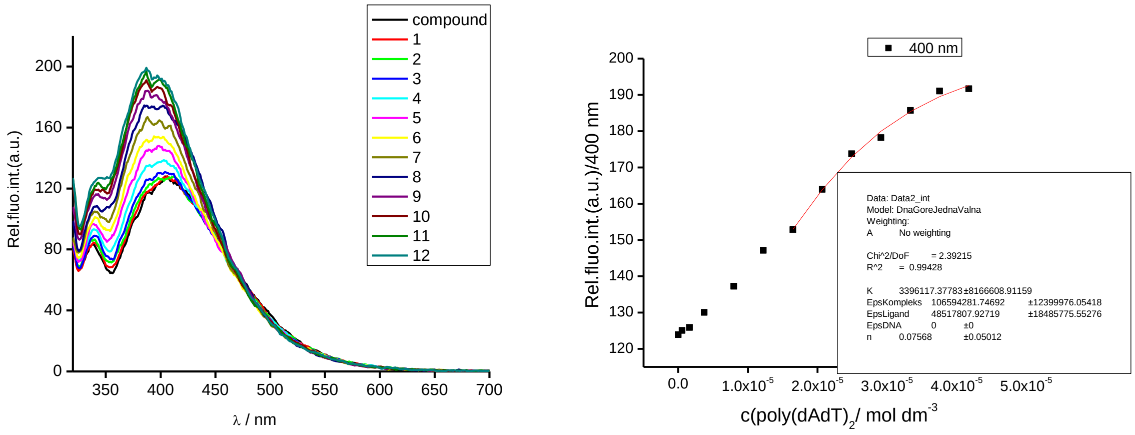

Figure S28. a) Changes in fluorescence spectrum of **5** (c= 2,0 × 10^{-6} mol dm^{-3}, λ_{exc}=302 nm) upon titration with poly $(dAdT)_2$ (c= 5.32 × 10^{-7} – 4.17× 10^{-5} mol dm^{-3}); b) Dependence of **5** absorbance at λ_{max}= 400 nm on c (poly $(dAdT)_2$), at pH=7, sodium cacodylate buffer, I = 0.05 mol dm^{-3}.

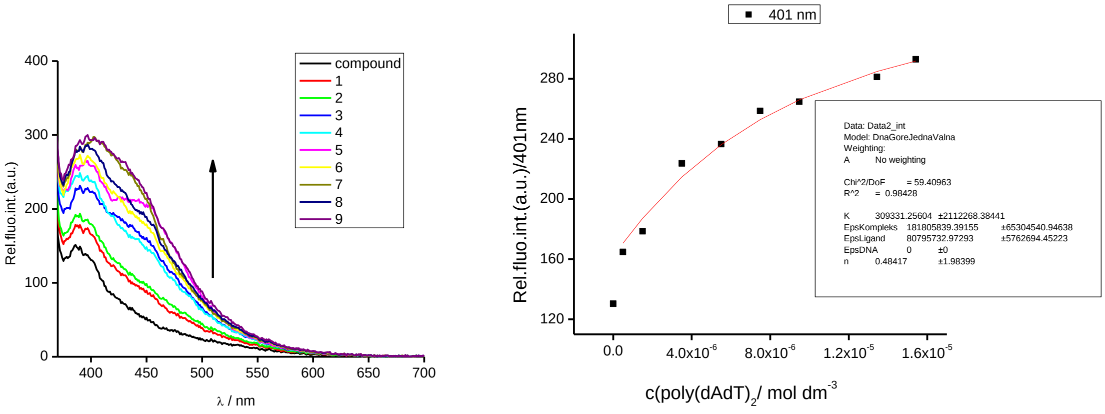

Figure S29. a) Changes in fluorescence spectrum of **8** (c= 2,0 × 10^{-6} mol dm^{-3}, λ_{exc}=343 nm) upon titration with poly $(dAdT)_2$ (c= 5.01 × 10^{-7} – 7.99× 10^{-6} mol dm^{-3}); b) Dependence of **8** absorbance at λ_{max}= 415 nm on c (poly $(dAdT)_2$), at pH=7, sodium cacodylate buffer, I = 0.05 mol dm^{-3}.

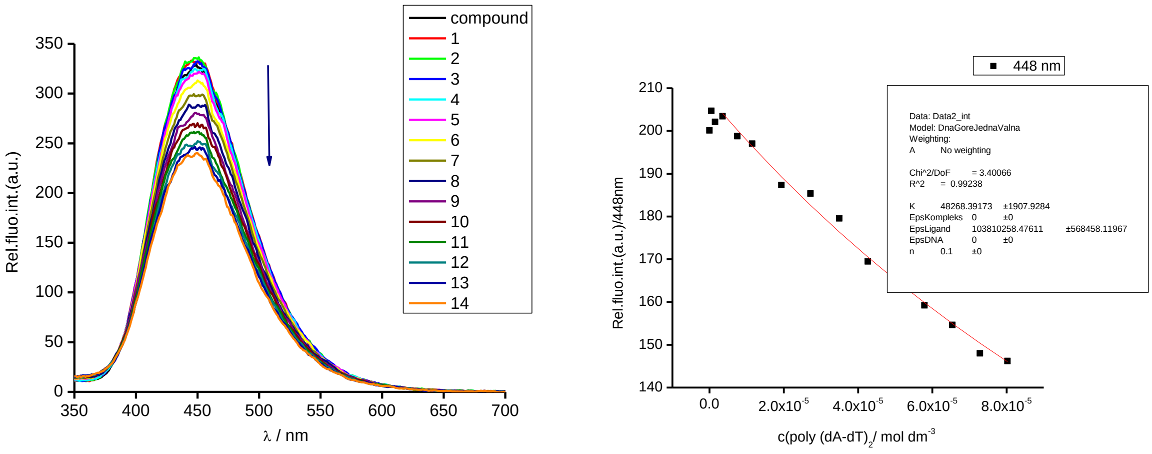

Figure S30. a) Changes in fluorescence spectrum of **20** (c= 2,0 × 10^{-6} mol dm^{-3}, λ_{exc}=343 nm) upon titration with poly $(dAdT)_2$ (c= 5.01 × 10^{-7}– 8.02× 10^{-5} mol dm^{-3}); b) Dependence of **20** absorbance at λ_{max}= 448 nm on c (poly $(dAdT)_2$), at pH=7, sodium cacodylate buffer, I = 0.05 mol dm^{-3}.

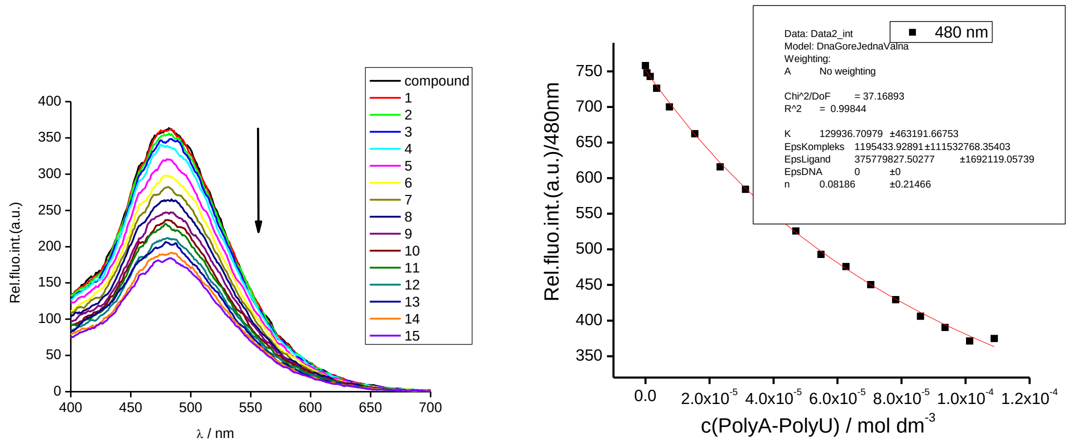

Figure S31. a) Changes in fluorescence spectrum of **9** (c= 2.0 × 10^{-6} mol dm^{-3}, λ_{exc}=315 nm) upon titration with poly A-poly U (c= 4.99 × 10^{-7}– 1.09× 10^{-4} mol dm^{-3}); b) Dependence of **9** absorbance at λ_{max} = 480 nm on c (poly A-poly U), at pH=7, sodium cacodylate buffer, I = 0.05 mol dm^{-3}.

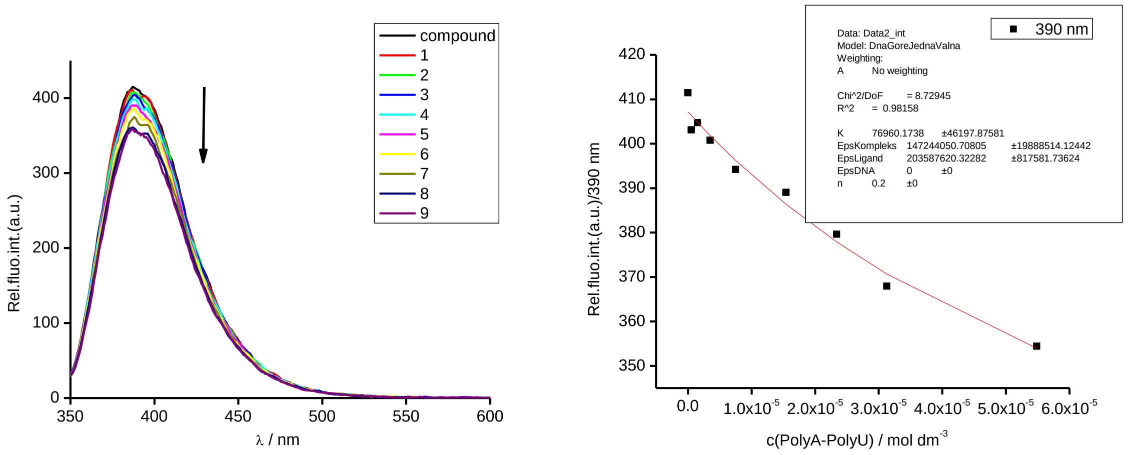

Figure S32. a) Changes in fluorescence spectrum of **11** (c= 2,0 × 10^{-6} mol dm^{-3}, λ_{exc}=321 nm) upon titration with poly A-poly U (c= 4.99 × 10^{-7} – 5.48× 10^{-5} mol dm^{-3}); b) Dependence of **11** absorbance at λ_{max}= 390 nm on c (poly A-ploy U), at pH=7, sodium cacodylate buffer, I = 0.05 mol dm^{-3}.

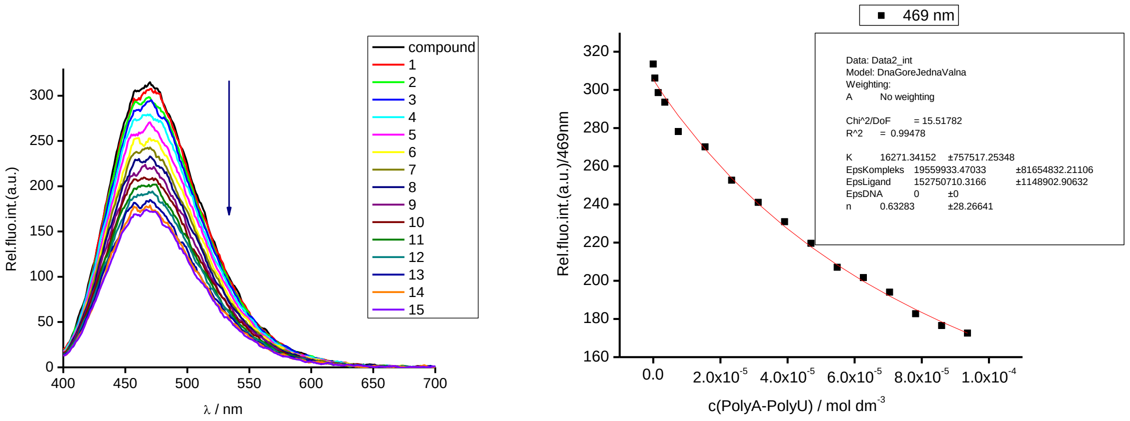

Figure S33. a) Changes in fluorescence spectrum of **6** (c= 2,0 × 10^{-6} mol dm^{-3}, λ_{exc}=322 nm) upon titration with poly A-poly U (c= 4.99× 10^{-7} – 9.36× 10^{-5} mol dm^{-3}); b) Dependence of **6** absorbance at λ_{max}= 469 nm on c (poly A-poly U), at pH=7, sodium cacodylate buffer, I = 0.05 mol dm^{-3}.

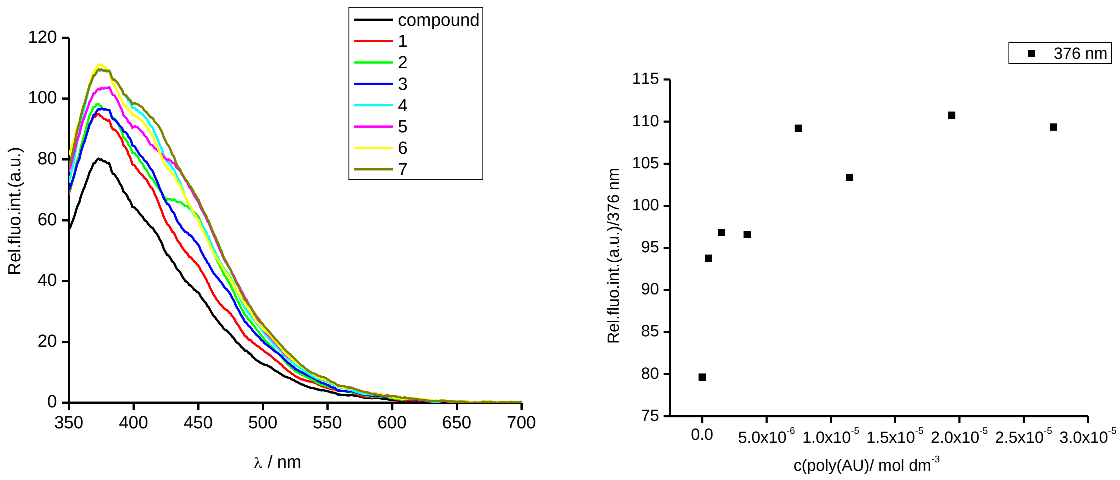

Figure S34. a) Changes in fluorescence spectrum of **10** (c= 2,0 × 10^{-6} mol dm^{-3}, λ_{exc}=302 nm) upon titration with poly A-poly U (c= 4.99 × 10^{-7} – 7.04× 10^{-5} mol dm^{-3}); b) Dependence of **10** absorbance at λ_{max}= 376 nm on c (poly A-poly U), at pH=7, sodium cacodylate buffer, I = 0.05 mol dm^{-3}.

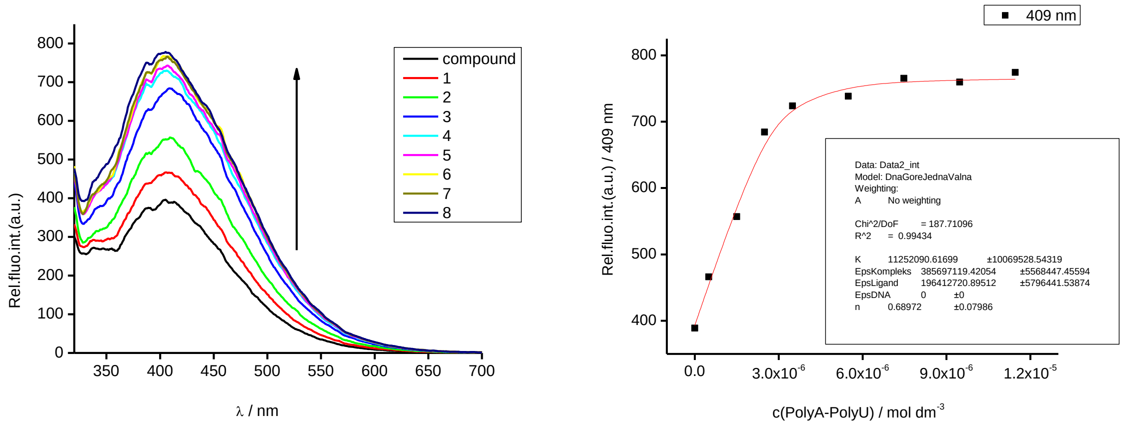

Figure S35. a) Changes in fluorescence spectrum of **5** (c= 2,0 × 10^{-6} mol dm^{-3}, λ_{exc}=302 nm) upon titration with poly A-poly U (c= 4.99 × 10^{-7} – 1.15× 10^{-5} mol dm^{-3}); b) Dependence of **5** absorbance at λ_{max}= 409 nm on c (poly A-poly U), at pH=7, sodium cacodylate buffer, I = 0.05 mol dm^{-3}.

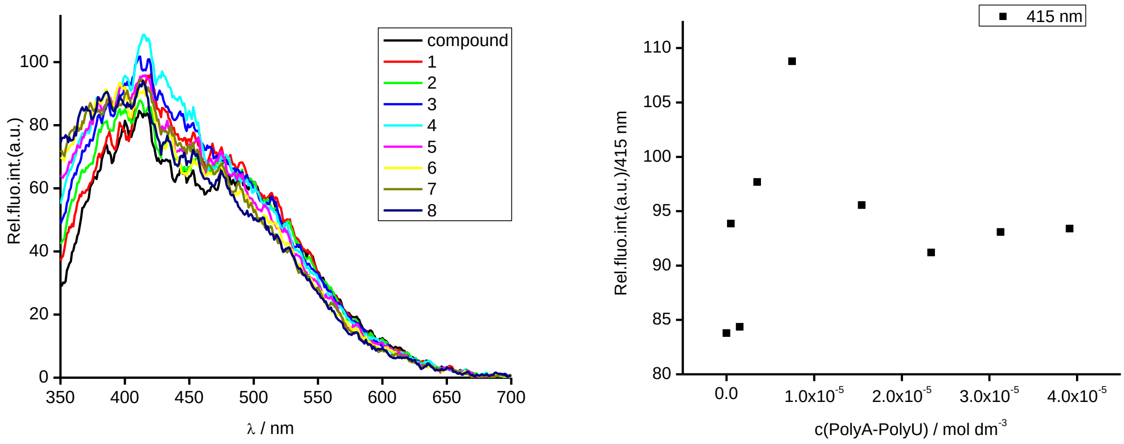

Figure S36. a) Changes in fluorescence spectrum of **8** (c= 2,0 × 10^{-6} mol dm^{-3}, λ_{exc}=343 nm) upon titration with poly A- poly U (c= 4.99× 10^{-7} – 3.92× 10^{-5} mol dm^{-3}); b) Dependence of **8** absorbance at λ_{max}= 415 nm on c (poly A- poly U, at pH=7, sodium cacodylate buffer, I = 0.05 mol dm^{-3}.

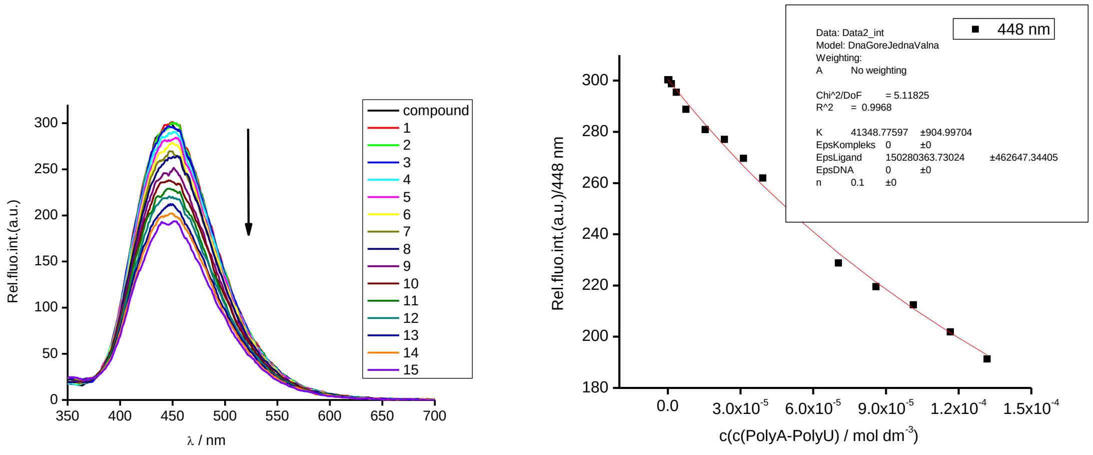

Figure S37. a) Changes in fluorescence spectrum of **20** (c= 2,0 × 10^{-6} mol dm^{-3}, λ_{exc}=322 nm) upon titration with poly A- poly U (c= 4.99 × 10^{-7} – 1.32× 10^{-4} mol dm^{-3}); b) Dependence of **20** absorbance at λ_{max}= 448 nm on c (poly A- poly U), at pH=7, sodium cacodylate buffer, I = 0.05 mol dm^{-3}.

2.2. Thermal melting experiments

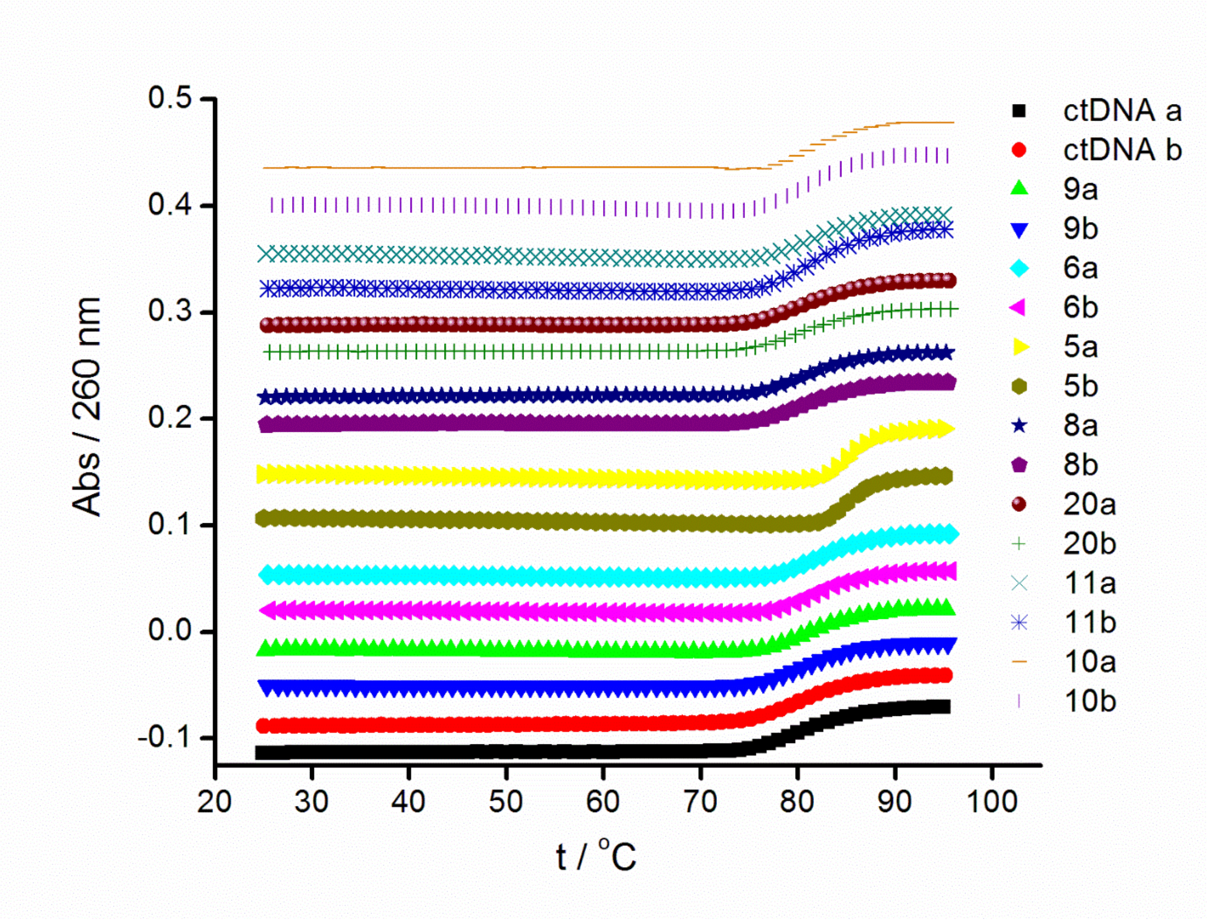

Figure S38. Melting curve of ctDNA upon addition of ratio, ***r*** ([compound]/ [polynucleotide])=0.3 of **5, 6, 8-11** and **20** at pH = 7.0 (buffer sodium cacodylate, *I* = 0.05 mol dm^{-3}).

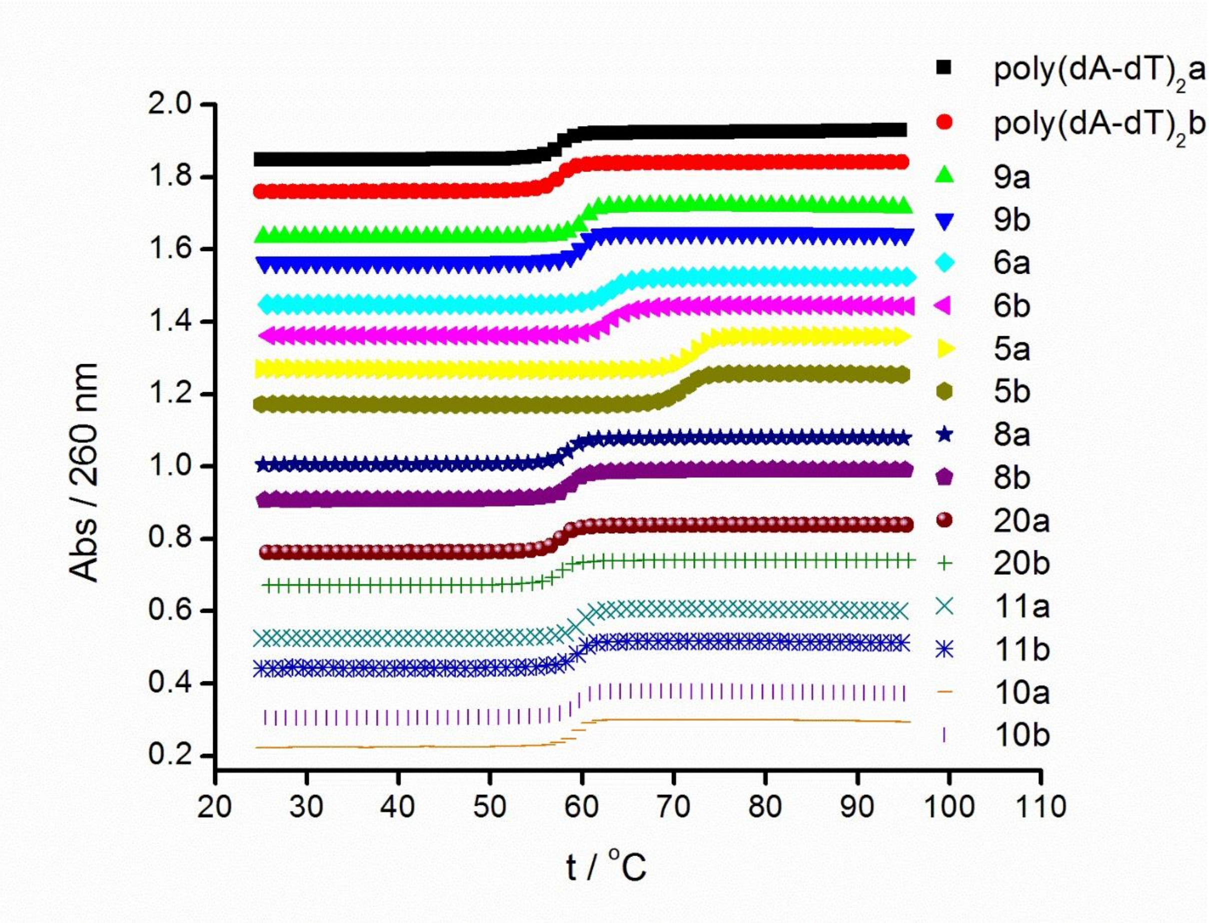

Figure S39. Melting curve of poly(dAdT)$_2$ upon addition of ratio, ***r*** ([compound/ [polynucleotide])=0.3 of **5, 6, 8-11** and **20** at pH = 7.0 (buffer sodium cacodylate, *I* = 0.05 mol dm^{-3}).

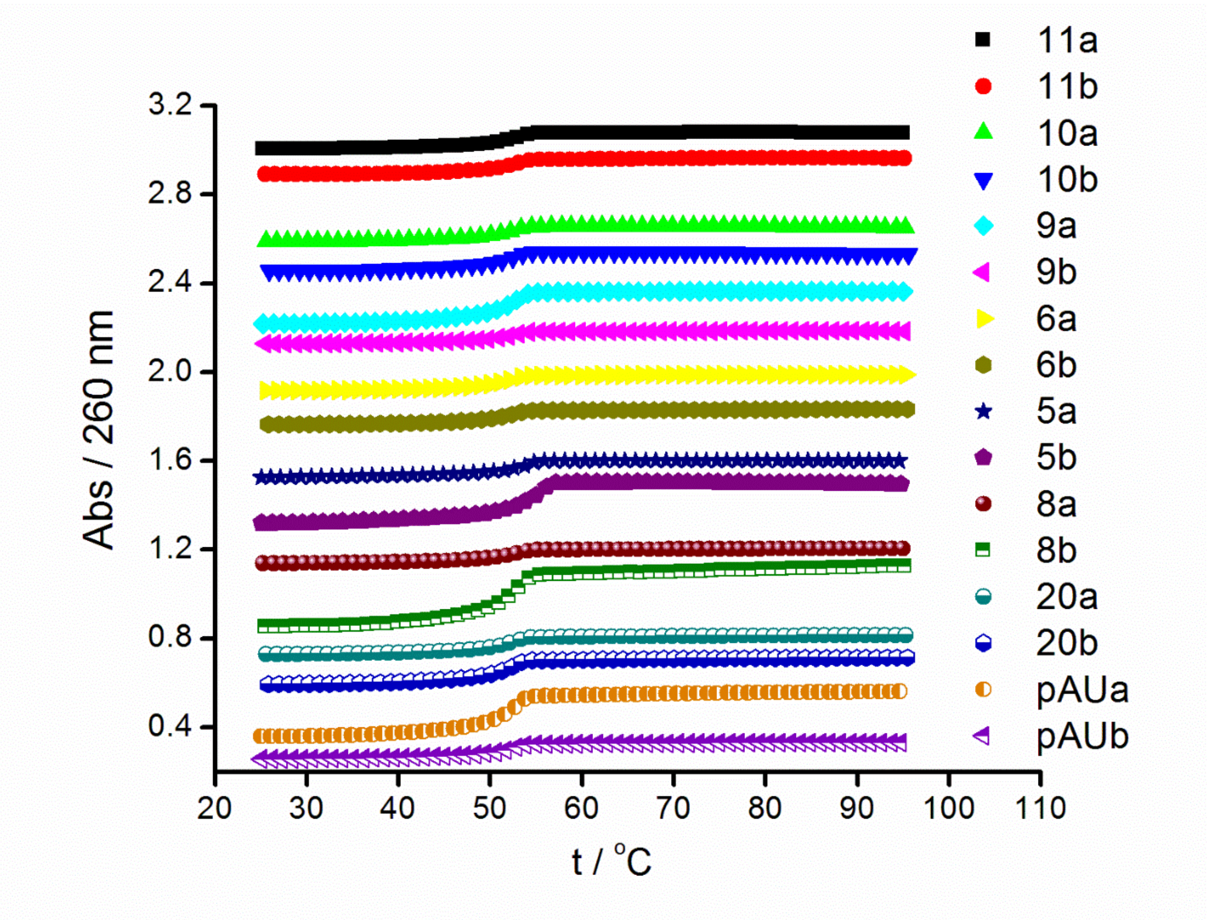

Figure S40. Melting curve of poly A – poly U upon addition of ratio, ***r*** ([compound/ [polynucleotide])=0.3 of **5, 6, 8-11** and **20** at pH = 7.0 (buffer sodium cacodylate, *I* = 0.05 mol dm^{-3}).

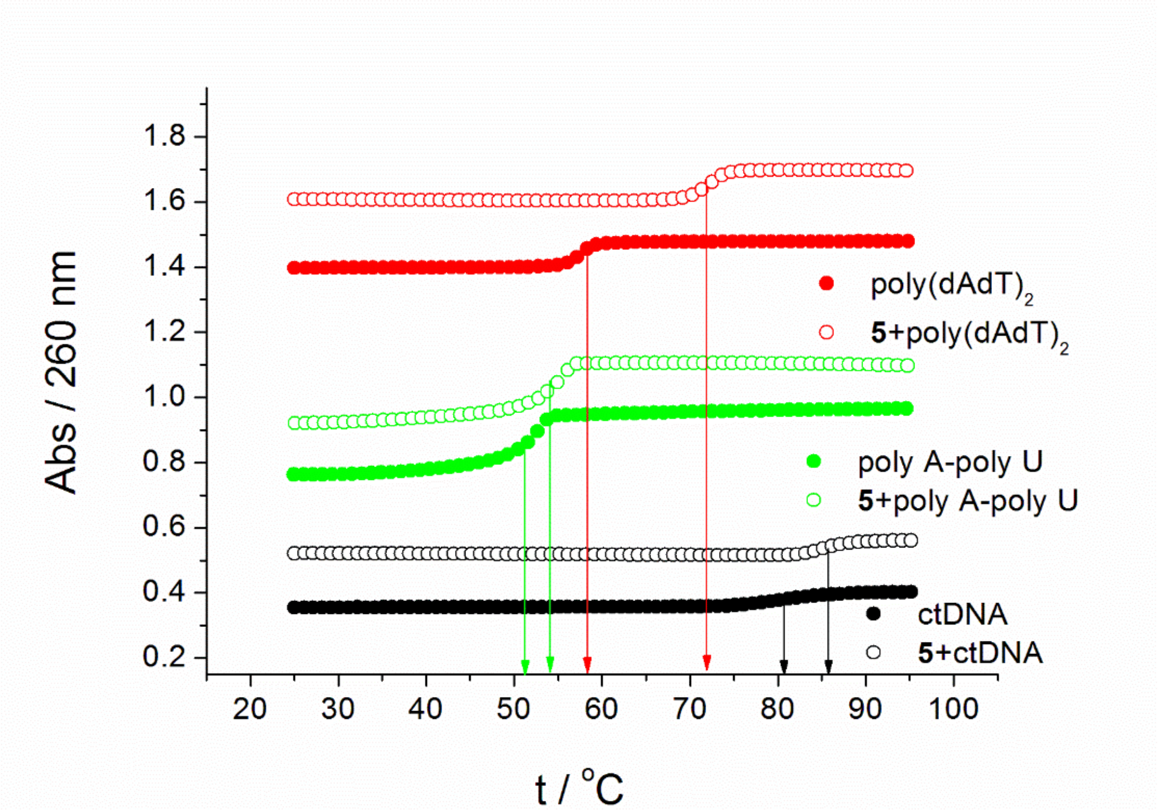

Figure S41. Melting curve of ctDNA, poly(dAdT)2 and poly A-poly U upon addition of ratio, r ([compound]/[polynucleotide]) = 0.3 of 5 at pH = 7.0 (buffer sodium cacodylate, I = 0.05 mol dm-3).

Table S2. The $^{a}\Delta T_m$ values (°C) of studied ds- polynucleotides upon addition of ratio br = 0.3 of **1**–**6** at pH = 7.0 (sodium cacodylate buffer, I = 0.05 mol dm-3).

$^{b}r = 0.3$	**5**	**6**	**8**	**9**	**10**	**11**	**20**
ctDNA	5	2	0	1	0	0	0
poly(dAdT)$_2$	14	6	1	3	2	2	0
poly A-poly U	2	0	0	0	0	0	0

[a] Error in ΔT_m : ± 0.5°C; [b] r = [compound] / [polynucleotide]

2.3. Circular dichroism (CD) titrations

	ctDNA	poly (dAdT)$_2$	poly A-poly U
9	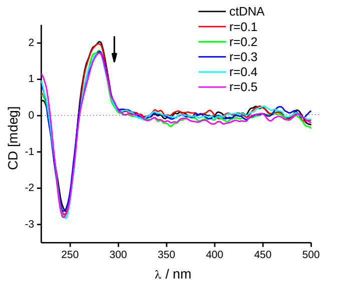	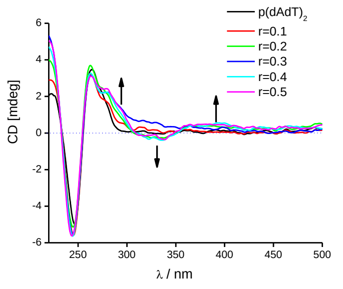	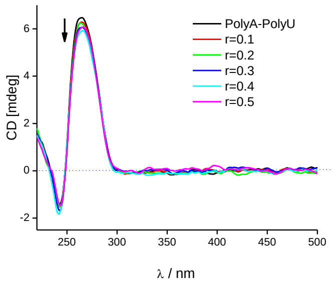
11	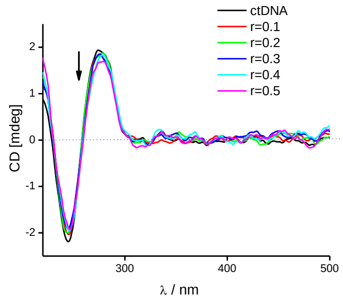	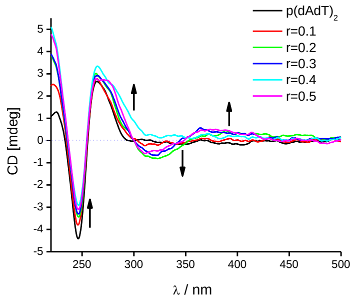	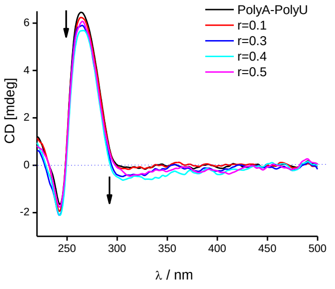
6	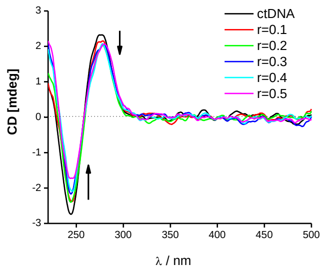	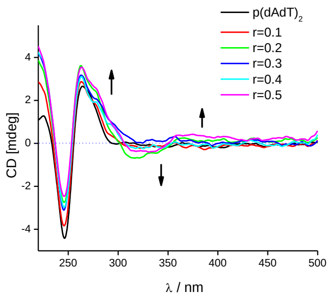	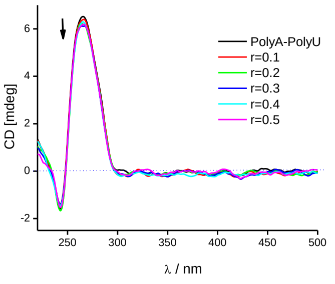
10	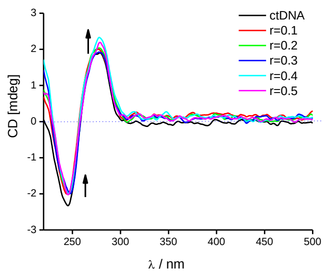	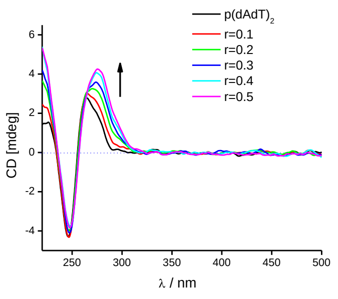	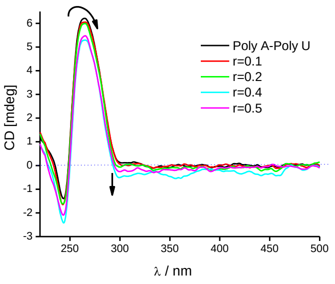
5	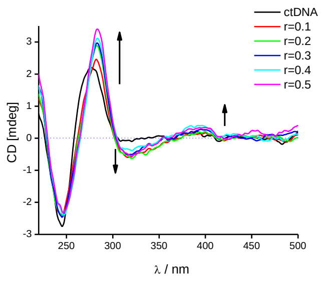	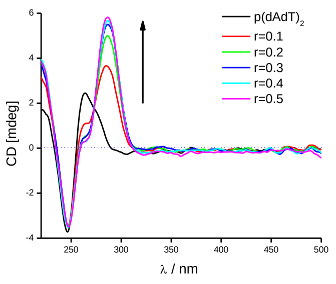	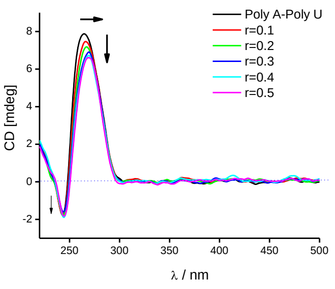

8	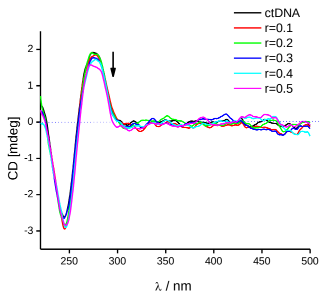	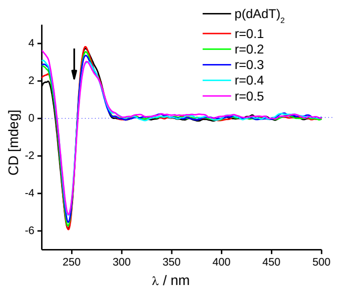	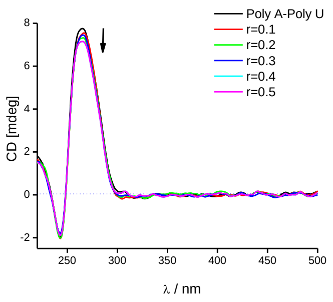
20	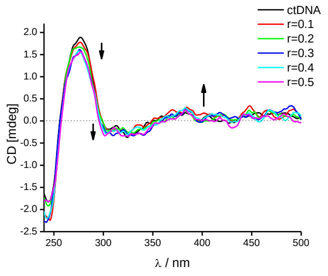	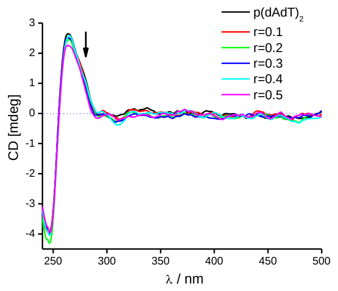	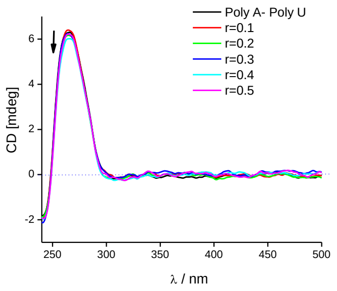

Figure S42. CD titration of ctDNA (c = 3.0 × 10^{-5} mol dm^{-3}), poly (dAdT)$_2$ (c = 3.0 × 10^{-5} mol dm^{-3}) and poly A-poly U (c = 3.0 × 10^{-5} mol dm^{-3}) with **5, 6, 8-11** and **20** at molar ratios ***r*** = [compound] / [polynucleotide] (pH = 7.0, buffer sodium cacodylate, I = 0.05 mol dm^{-3}).

3. ^{1}H and ^{13}C spectra of compounds 4–22

Figure S43: a) ^{1}H NMR and b) ^{13}C NMR of compd. **4**.

a)

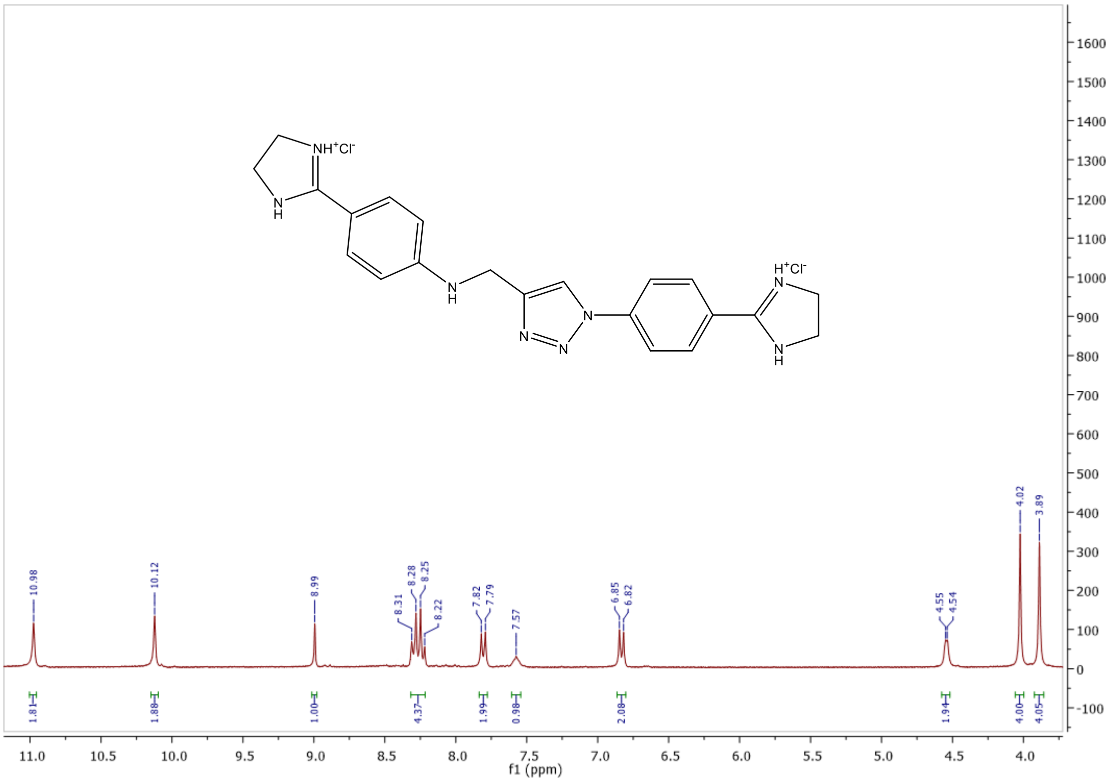

b)

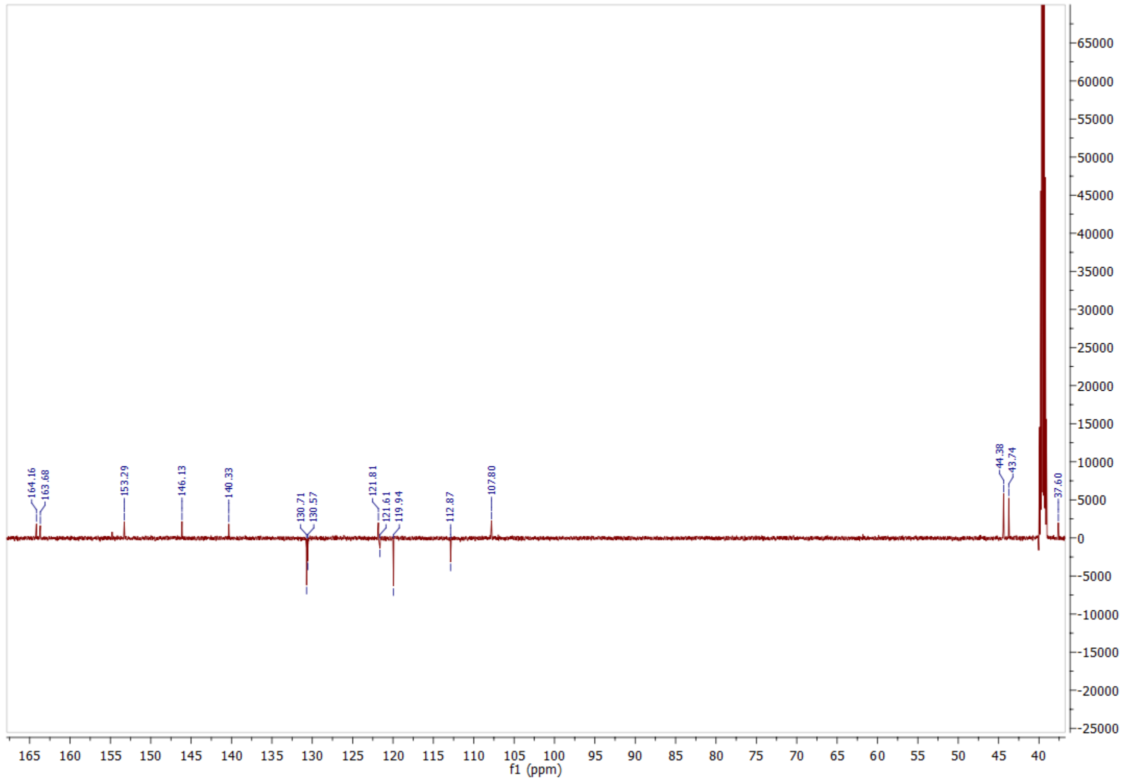

Figure S44: a) ^{1}H NMR and b) ^{13}C NMR of compd. **5**.

a)

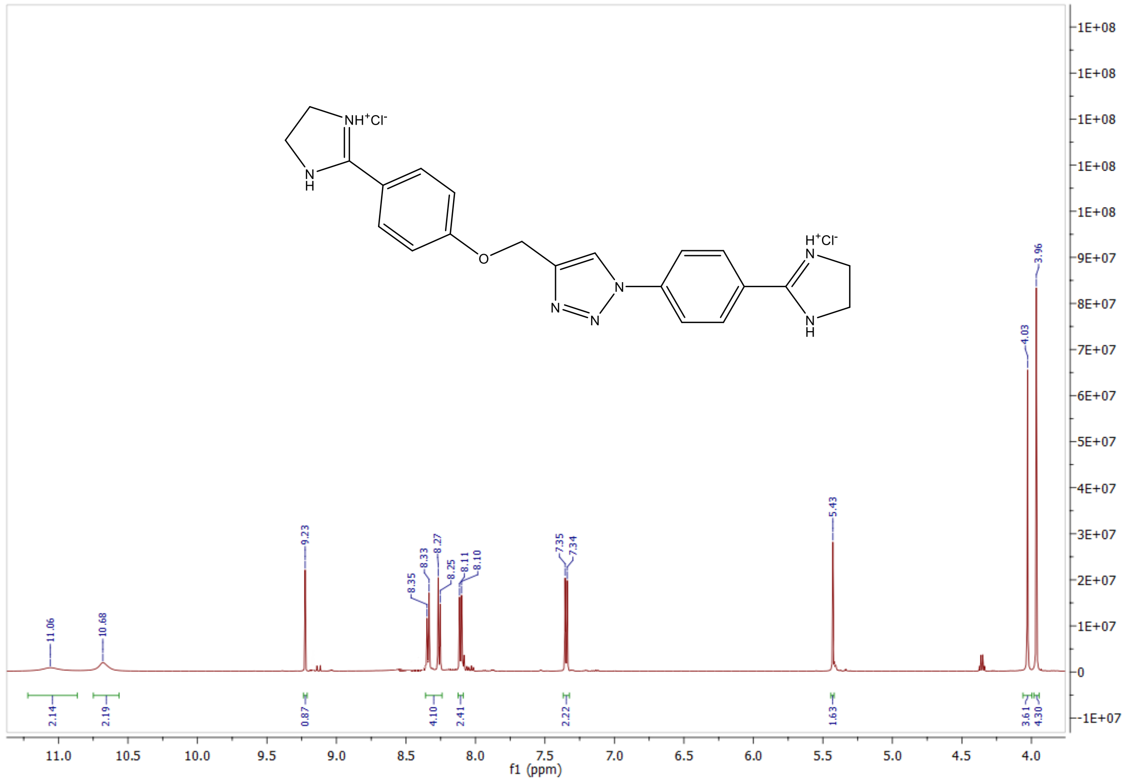

b)

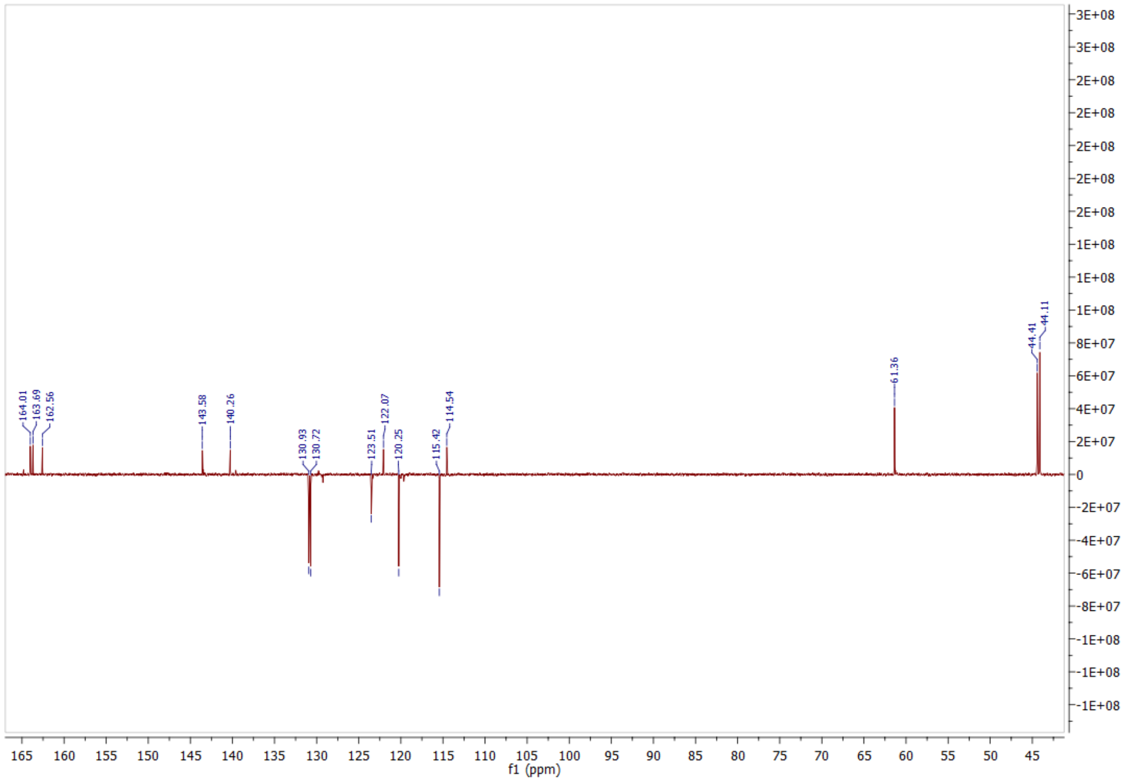

Figure S45: a) ^{1}H NMR and b) ^{13}C NMR of compd. **6**.

a)

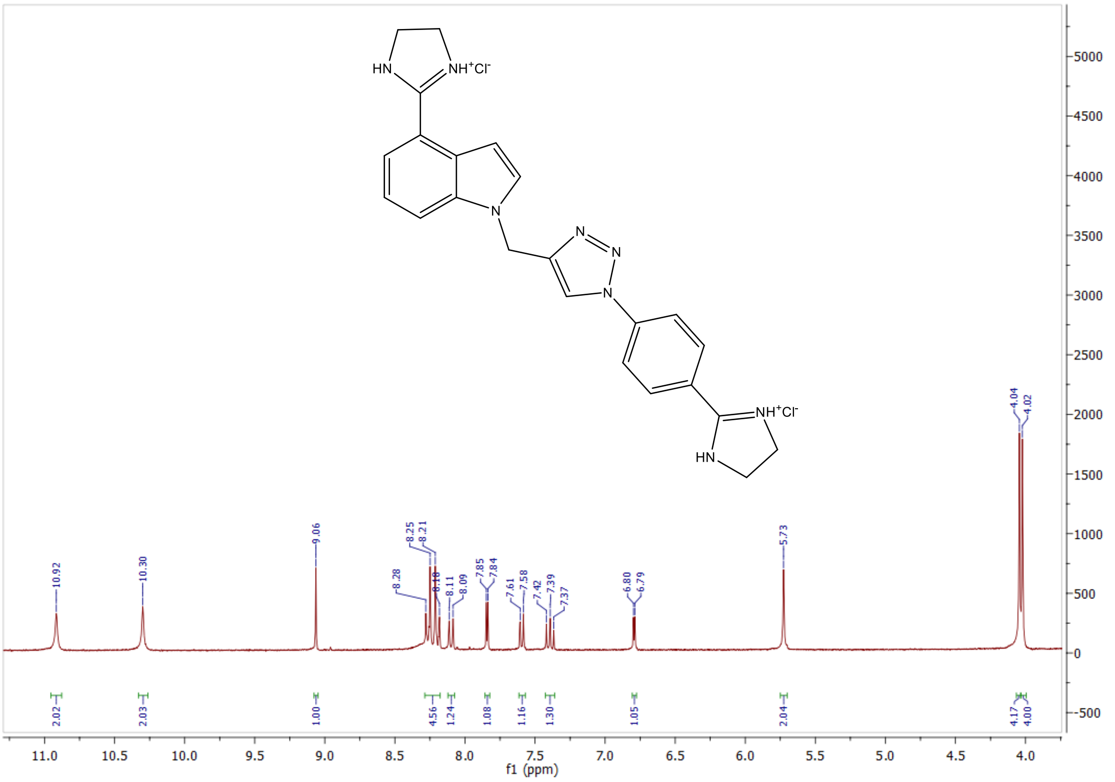

b)

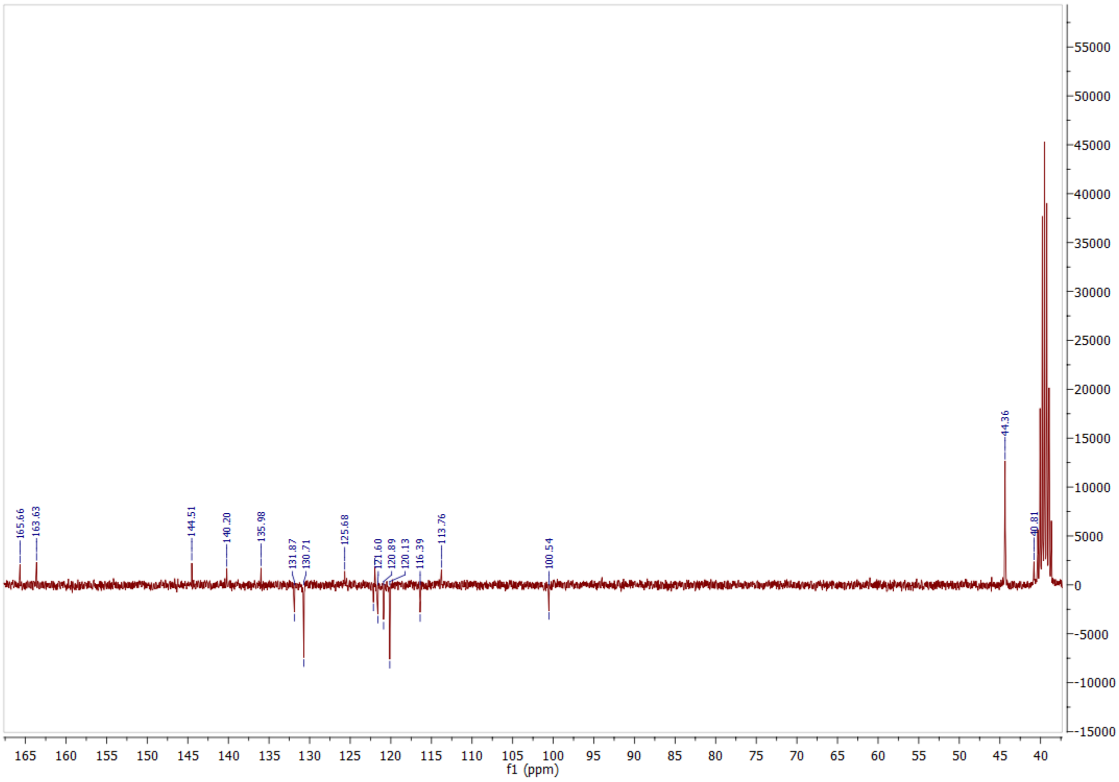

Figure S46: a) ^{1}H NMR and b) ^{13}C NMR of compd. **7**.

a)

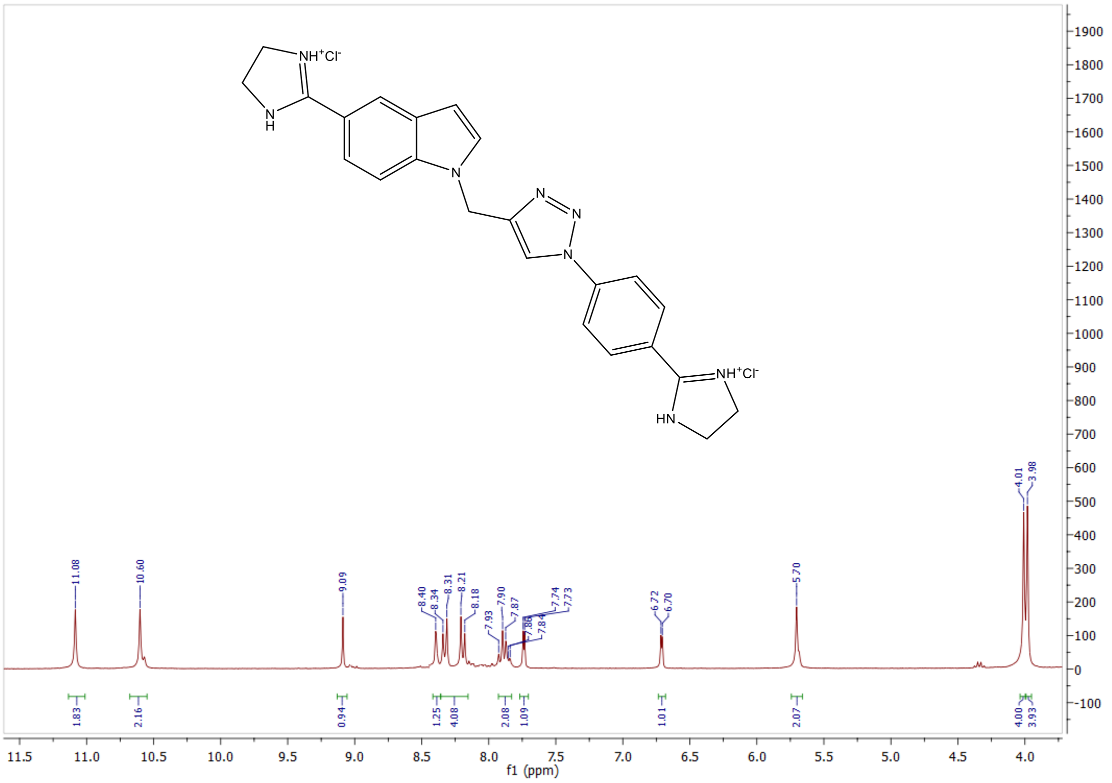

b)

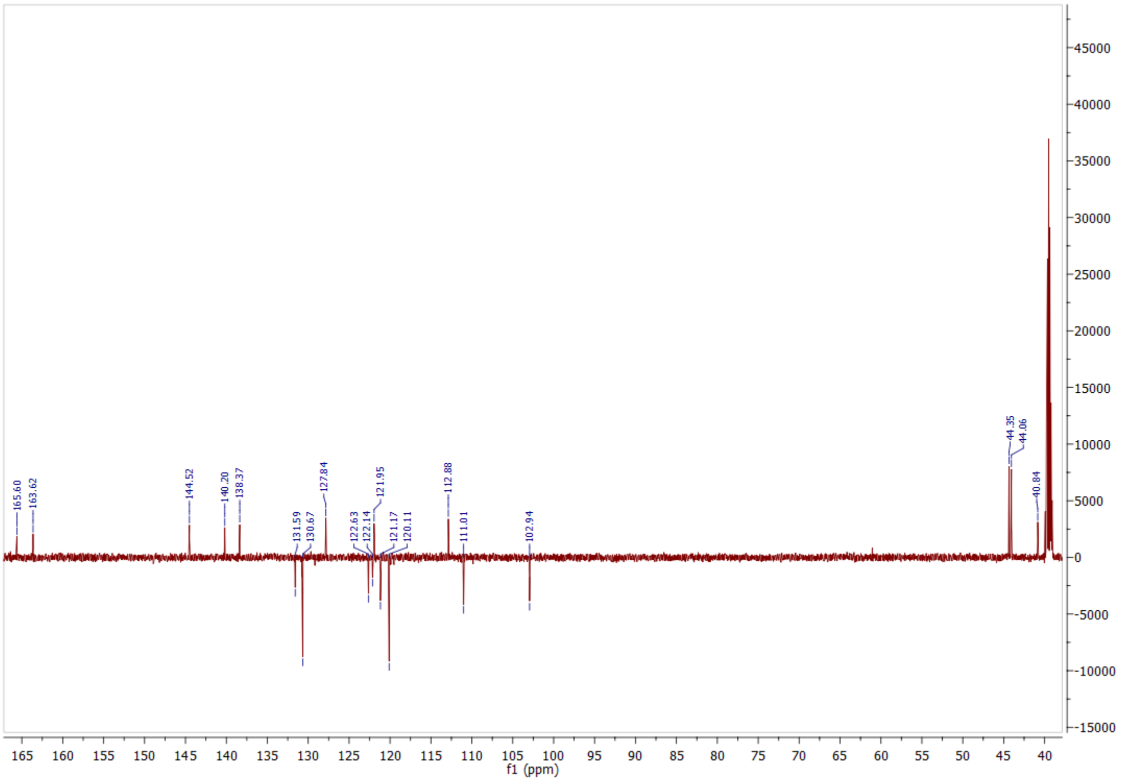

Figure S47: a) ^{1}H NMR and b) ^{13}C NMR of compd. **8**.

a)

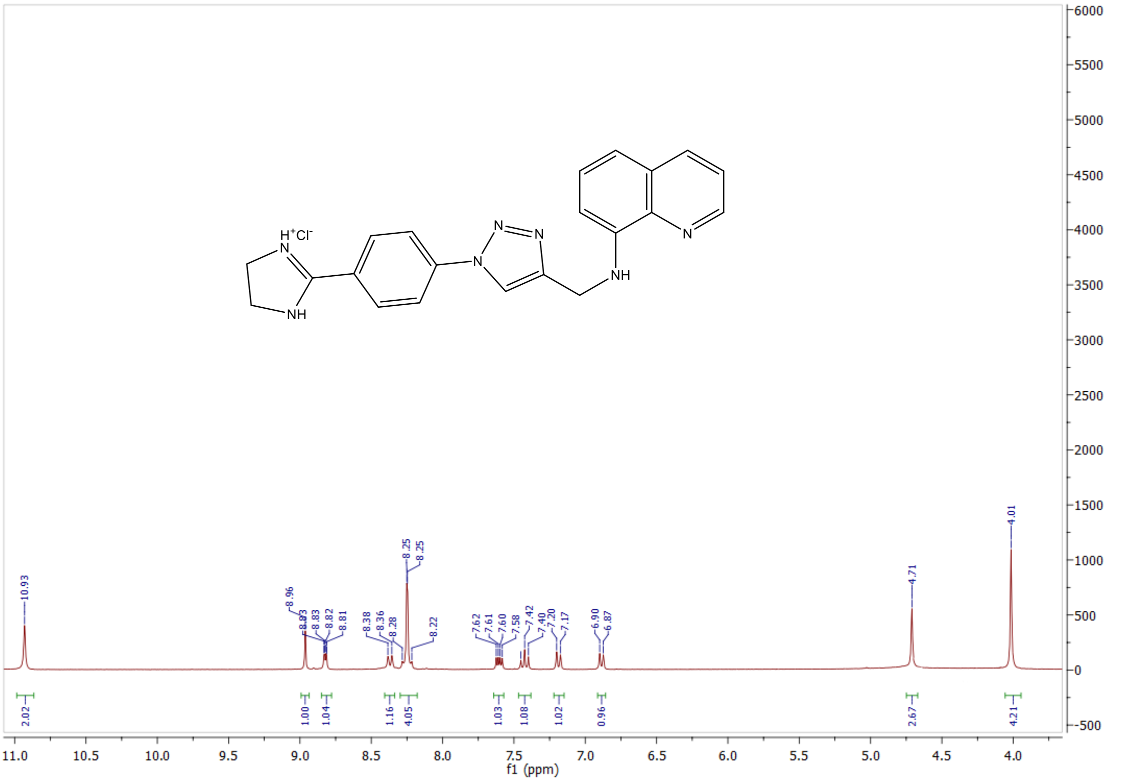

b)

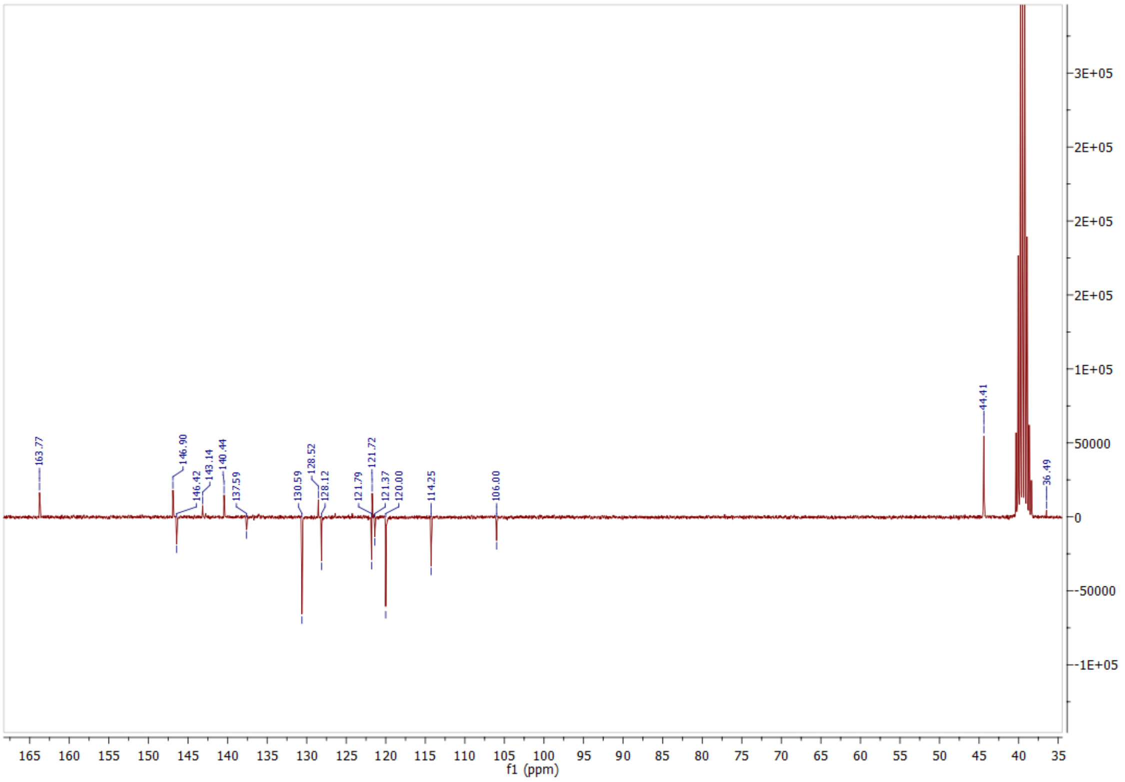

Figure S48: a) ^{1}H NMR and b) ^{13}C NMR of compd. **9**.

a)

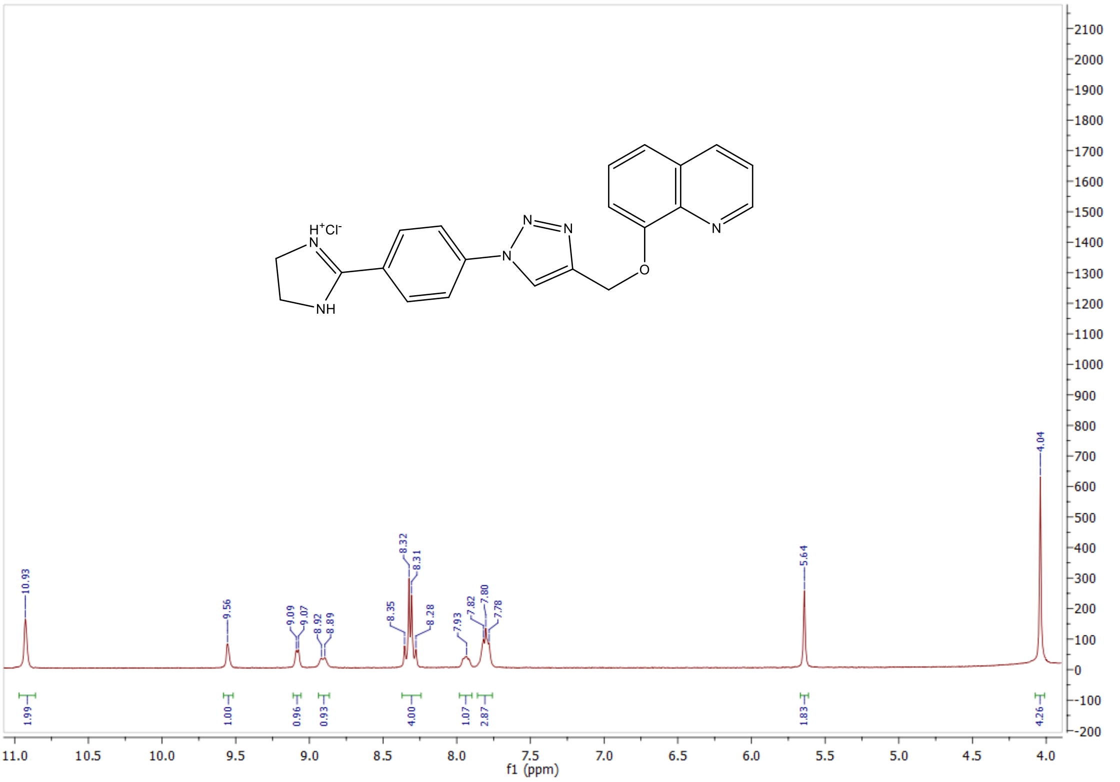

b)

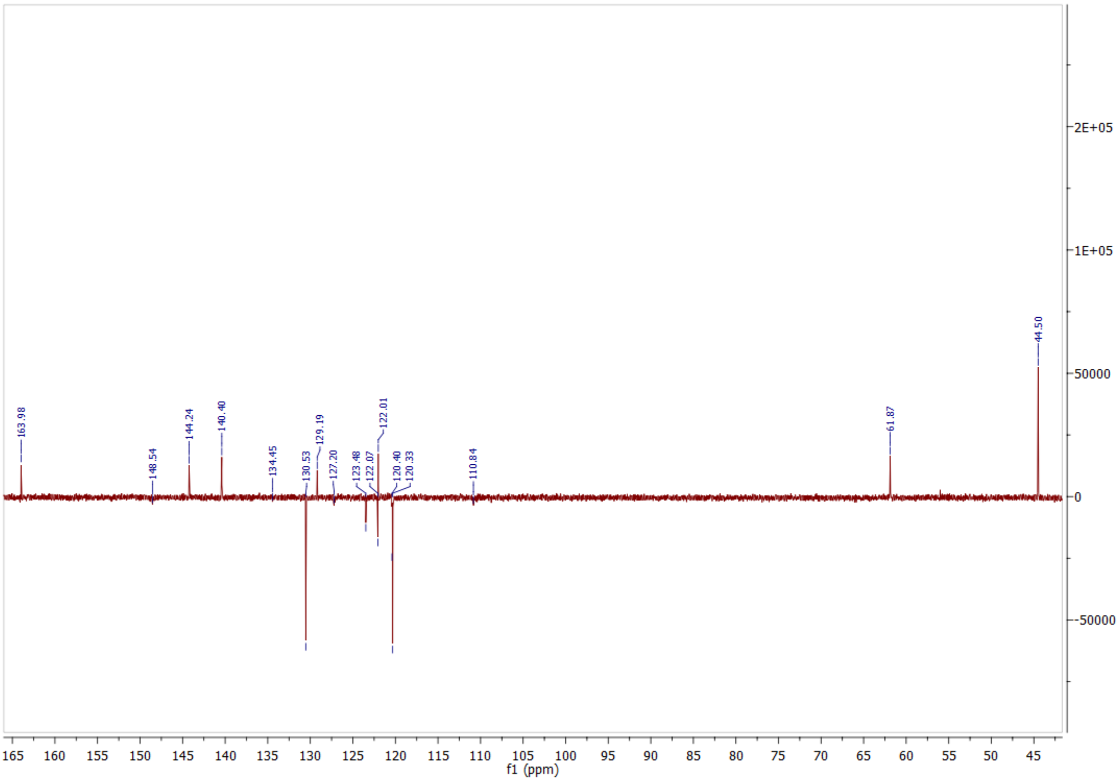

Figure S49: a) ^{1}H NMR and b) ^{13}C NMR of compd. **10**.

a)

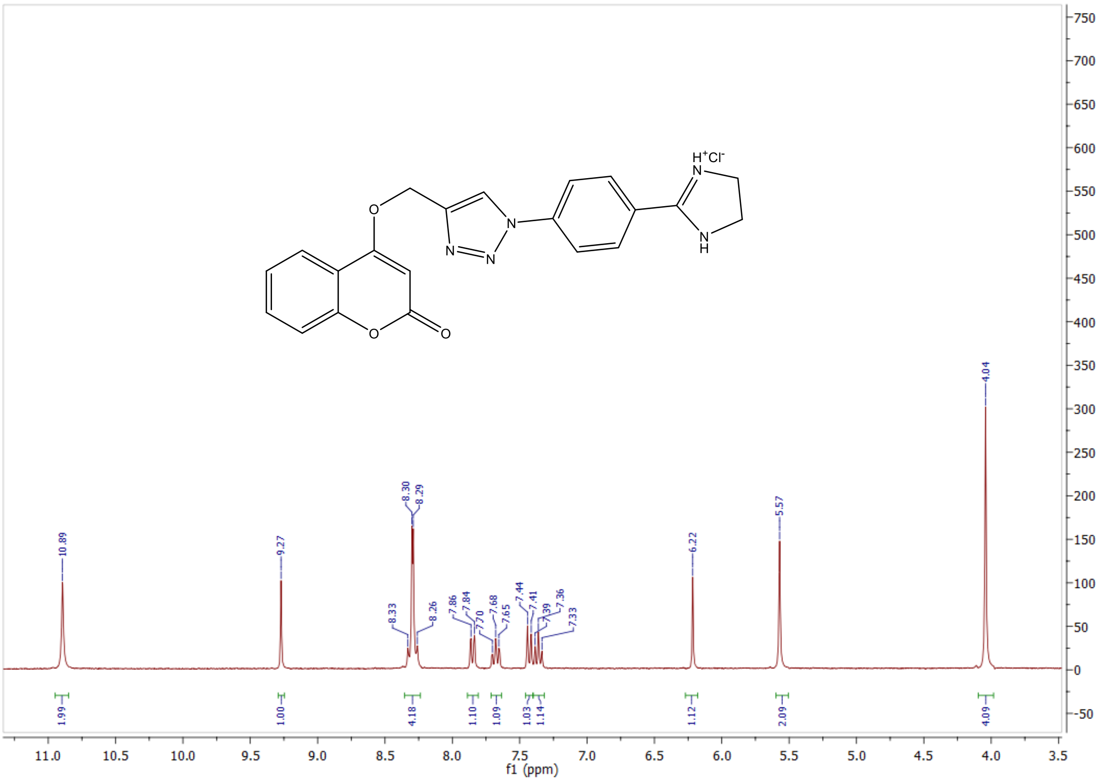

b)

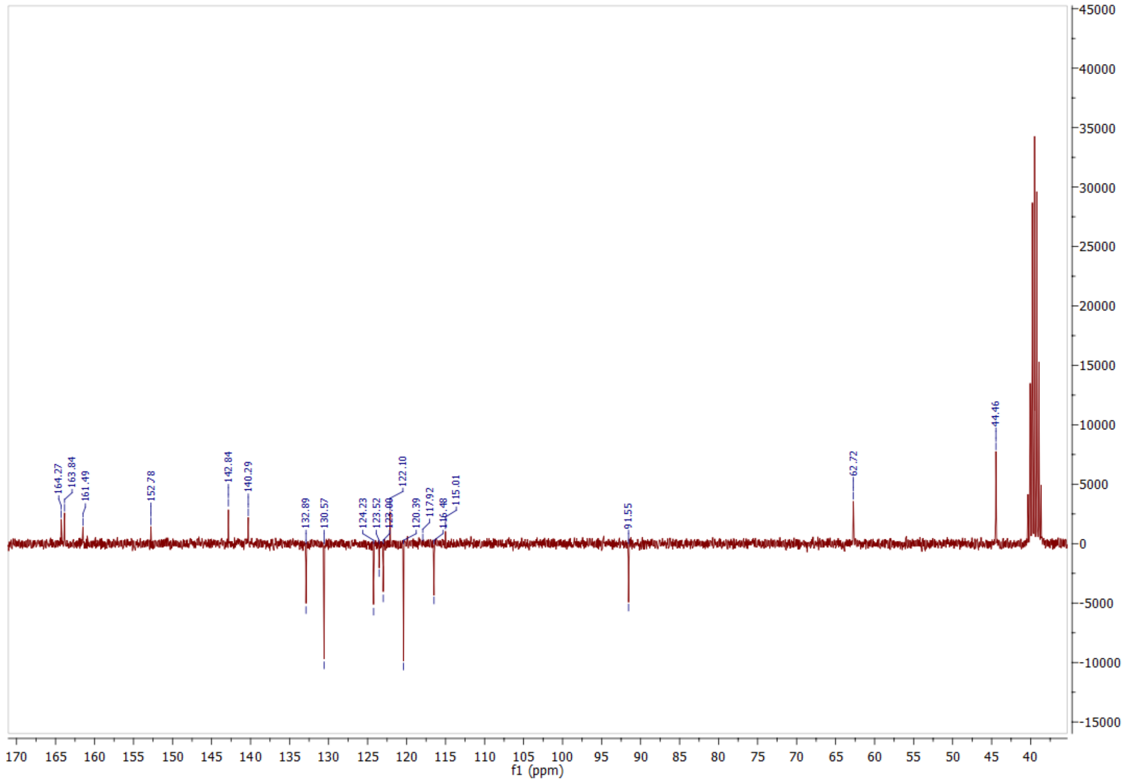

Figure S50: a) ^{1}H NMR and b) ^{13}C NMR of compd. **11**.

a)

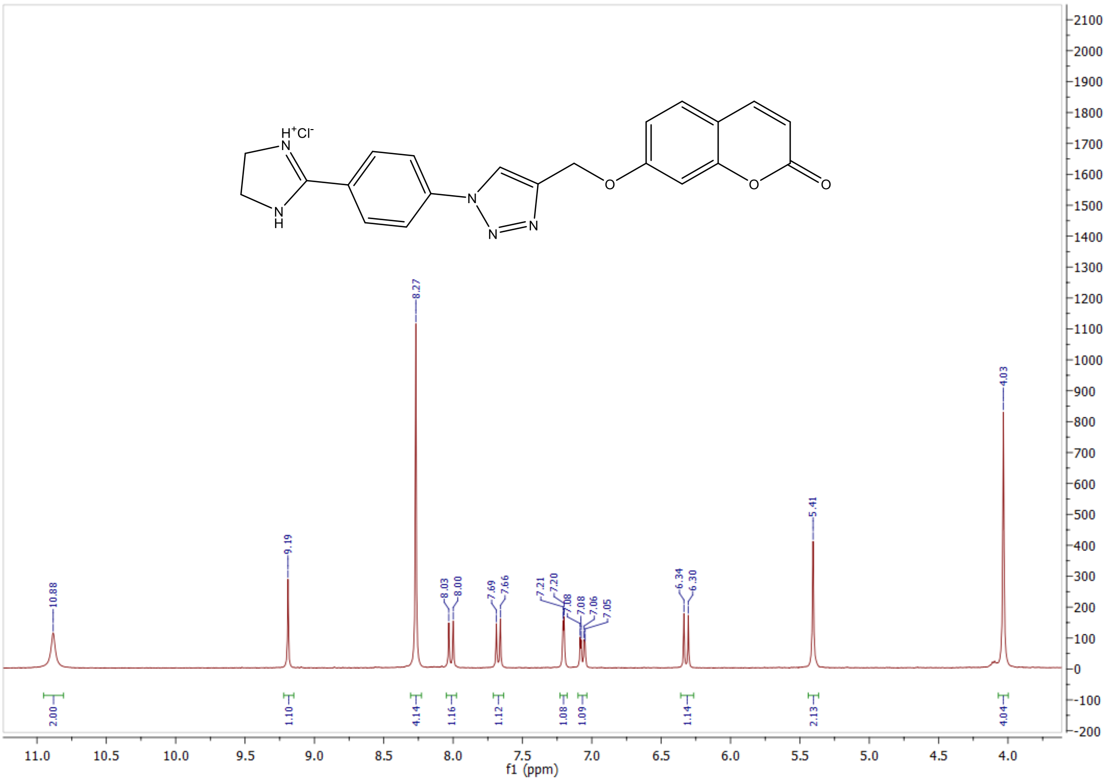

b)

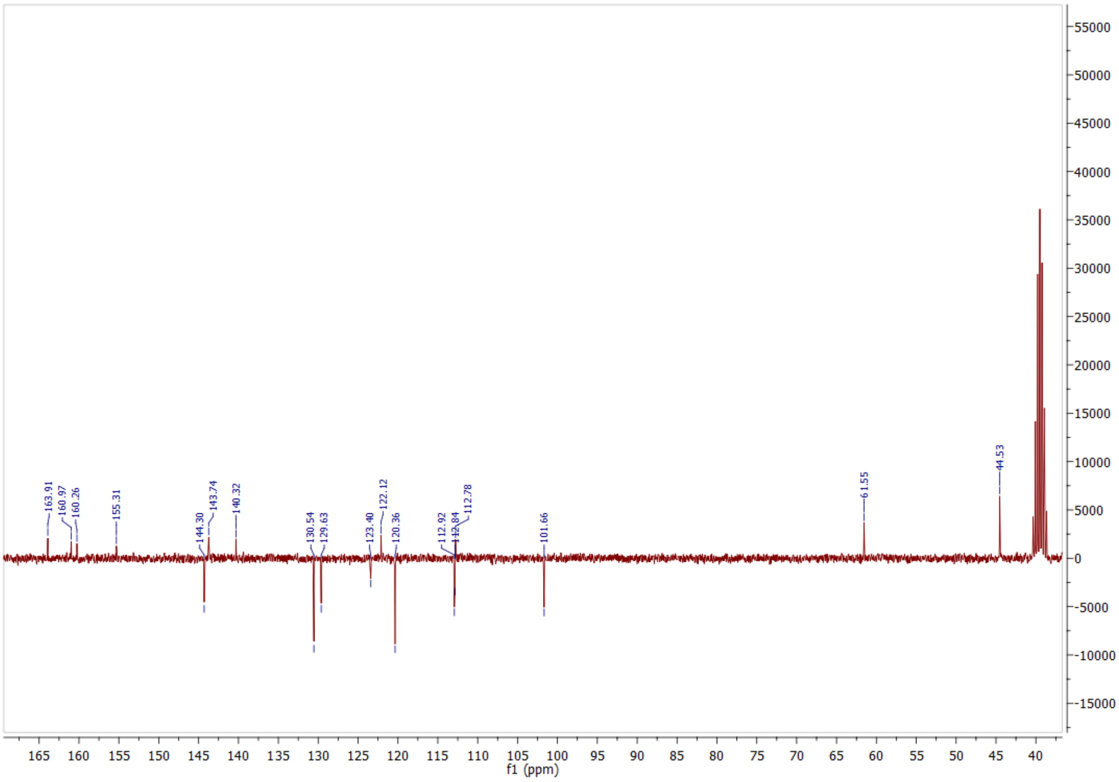

Figure S51: a) ^{1}H NMR and b) ^{13}C NMR of compd. **12**.

a)

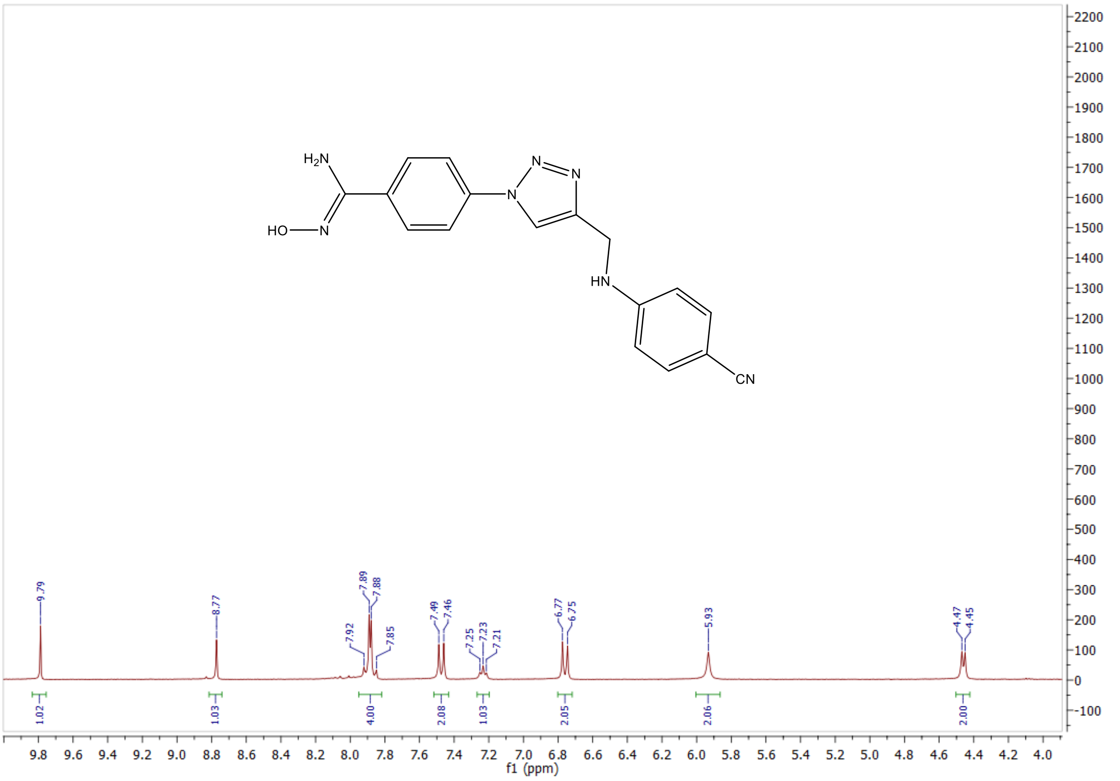

b)

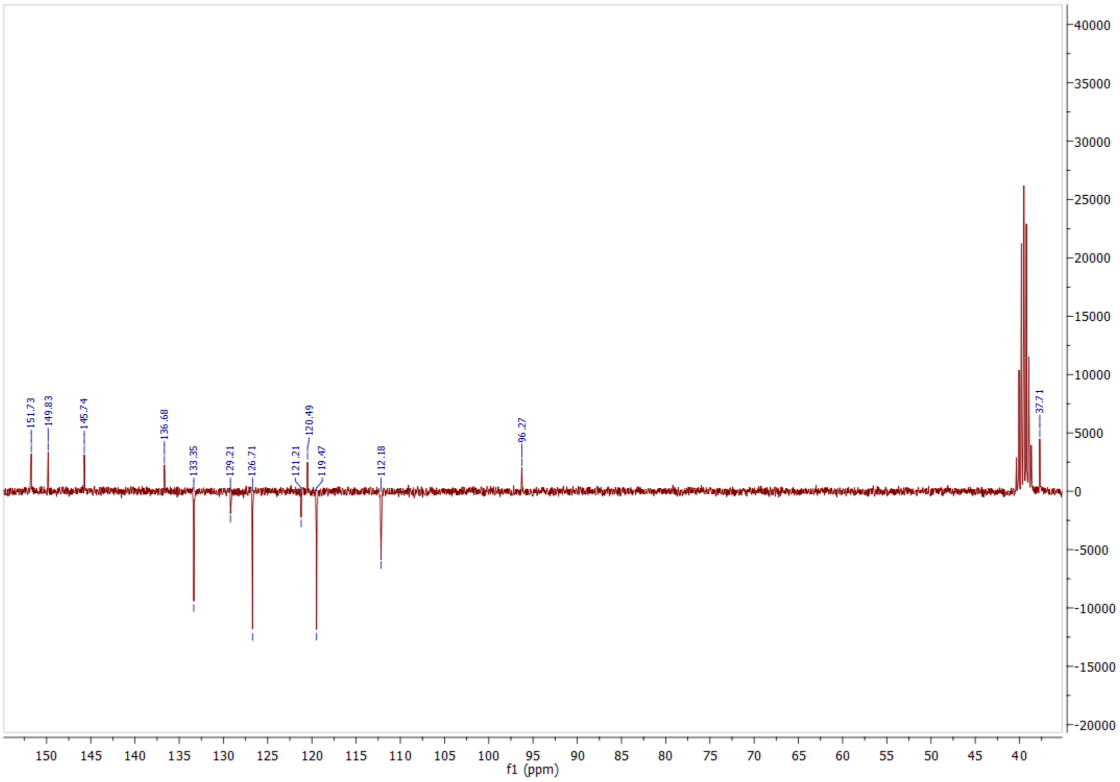

Figure S52: a) ^{1}H NMR and b) ^{13}C NMR of compd. **13**.

a)

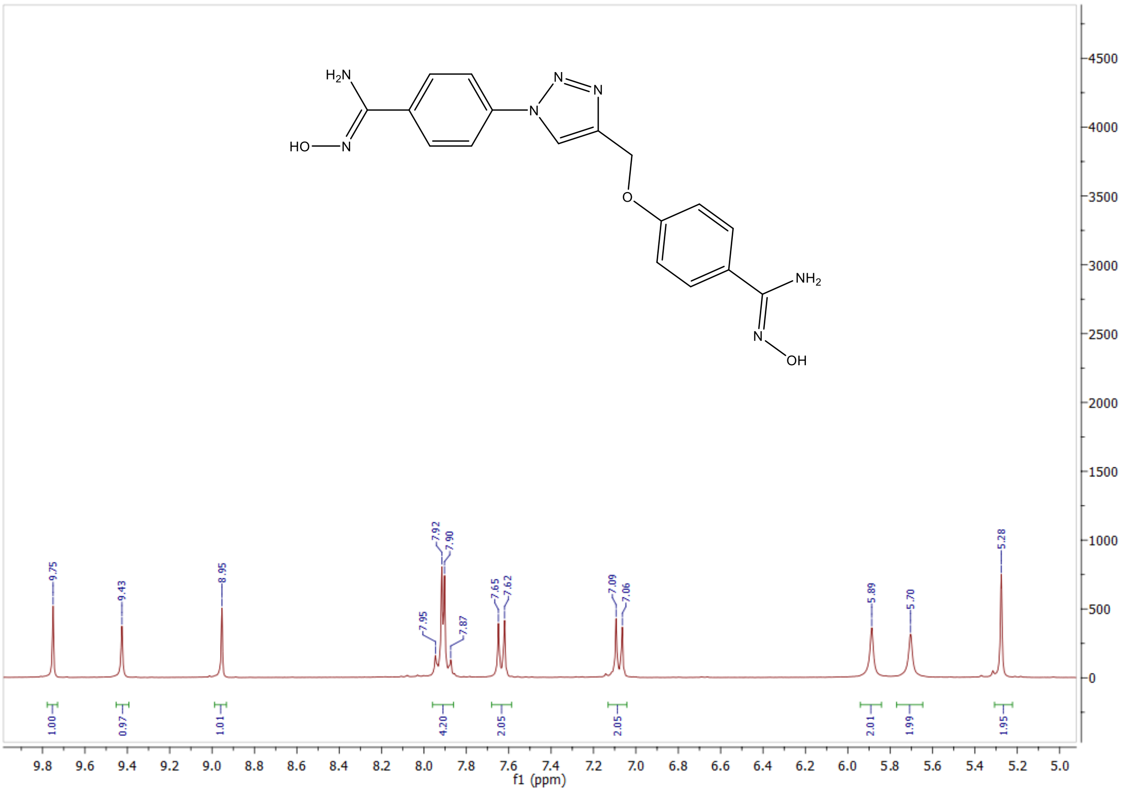

b)

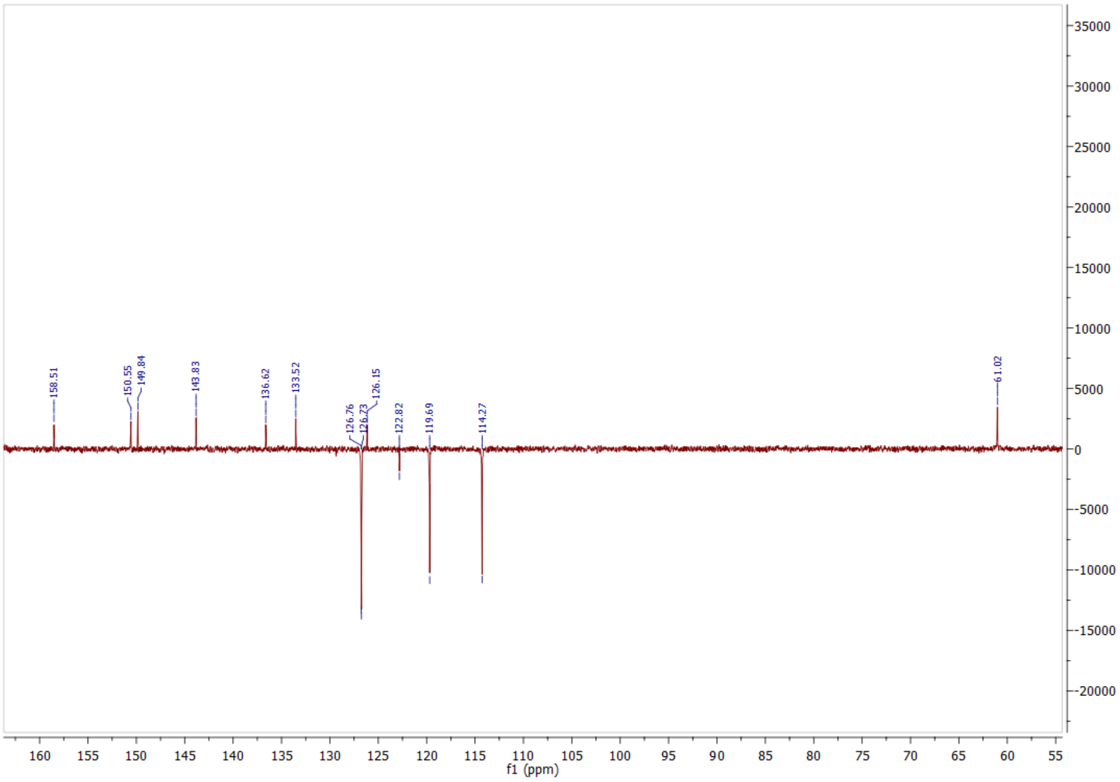

Figure S53: a) ^{1}H NMR and b) ^{13}C NMR of compd. **14**.

a)

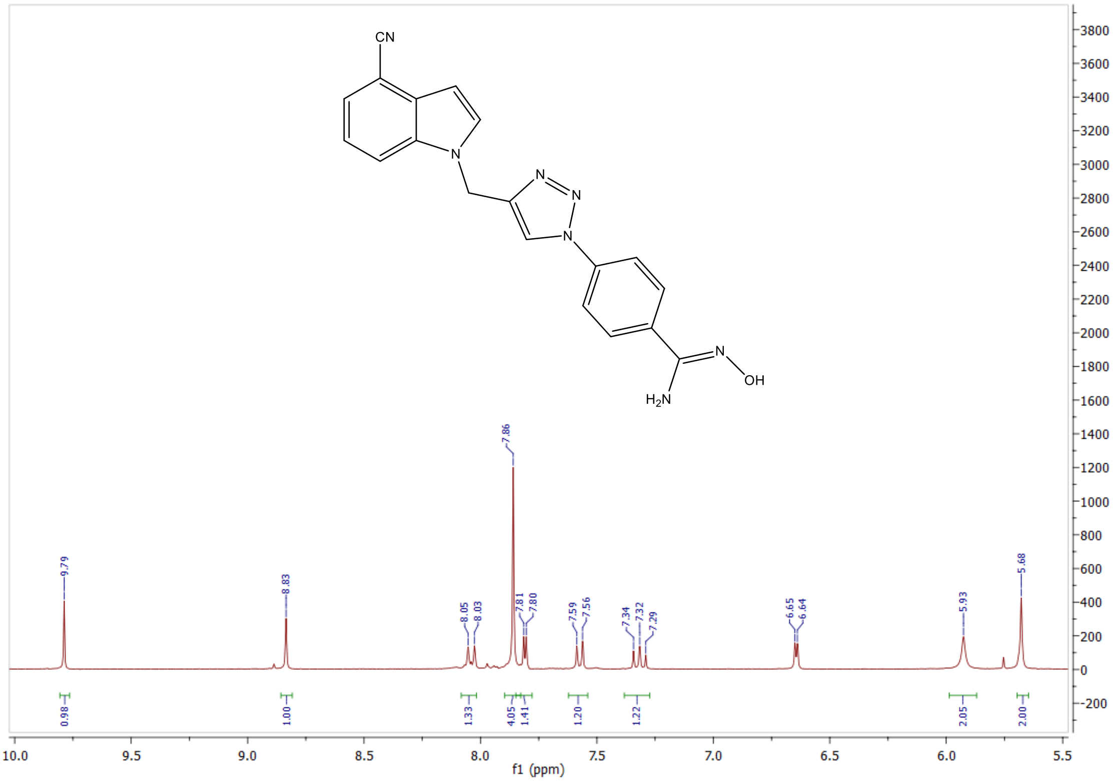

b)

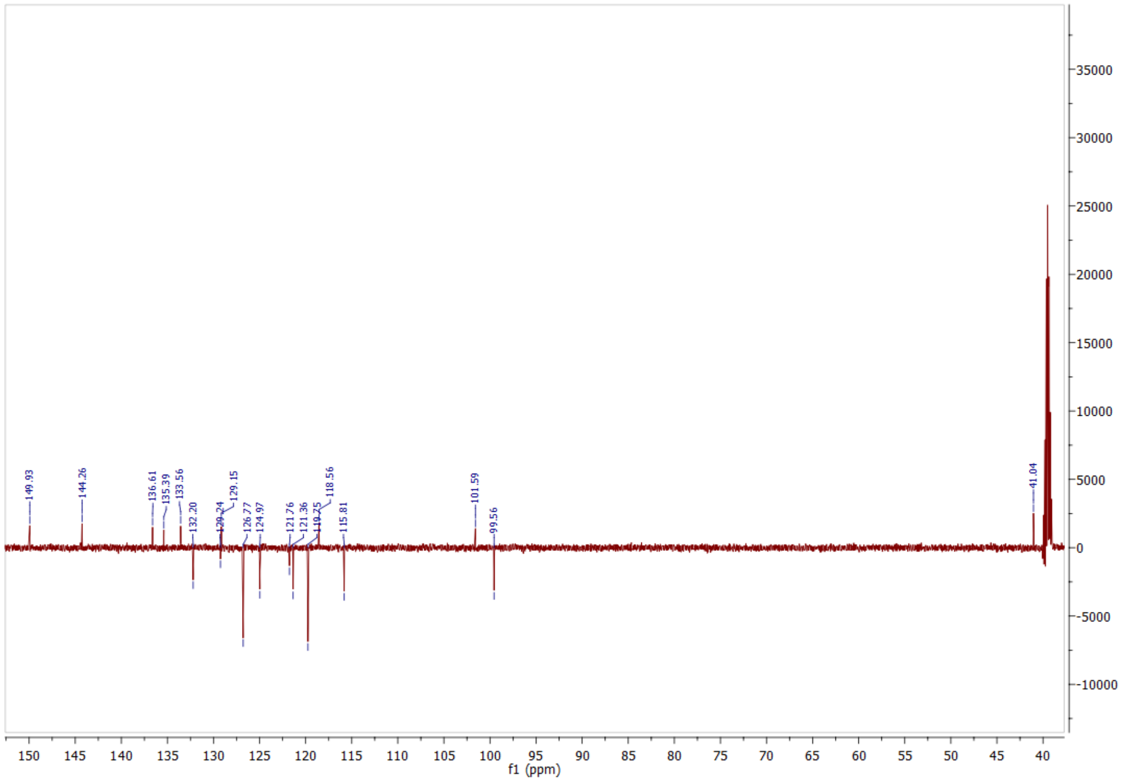

Figure S54: a) ^{1}H NMR and b) ^{13}C NMR of compd. **15**.

a)

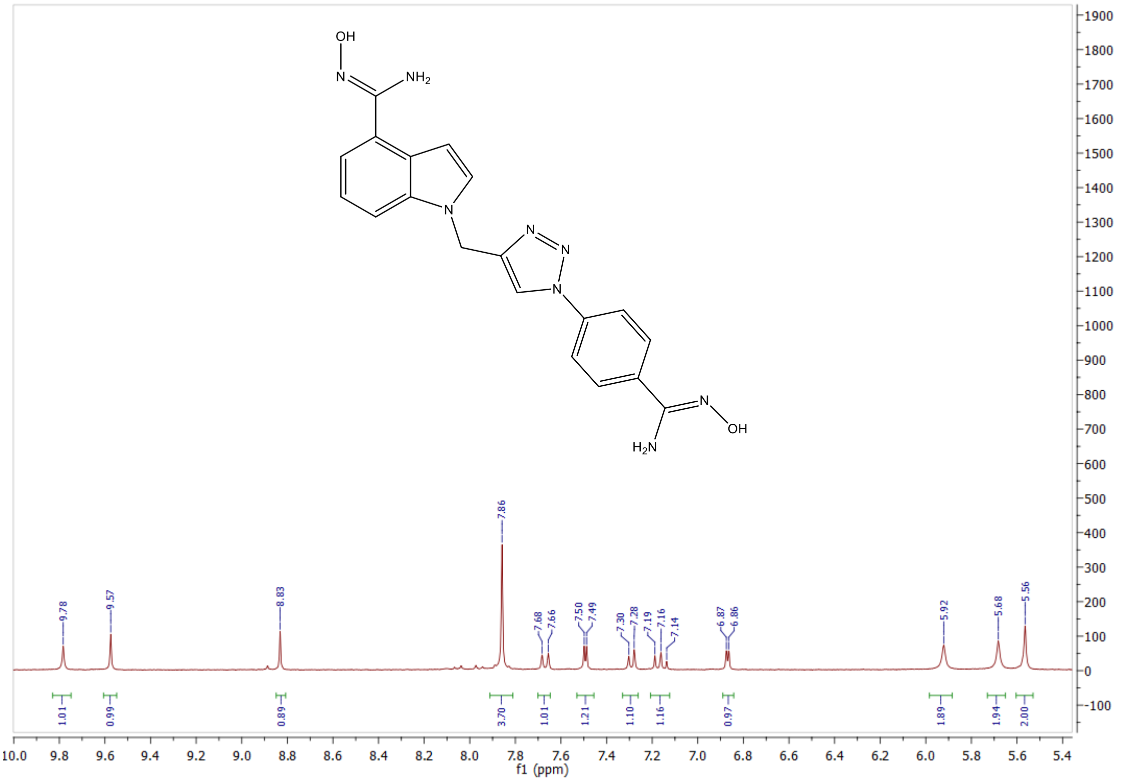

b)

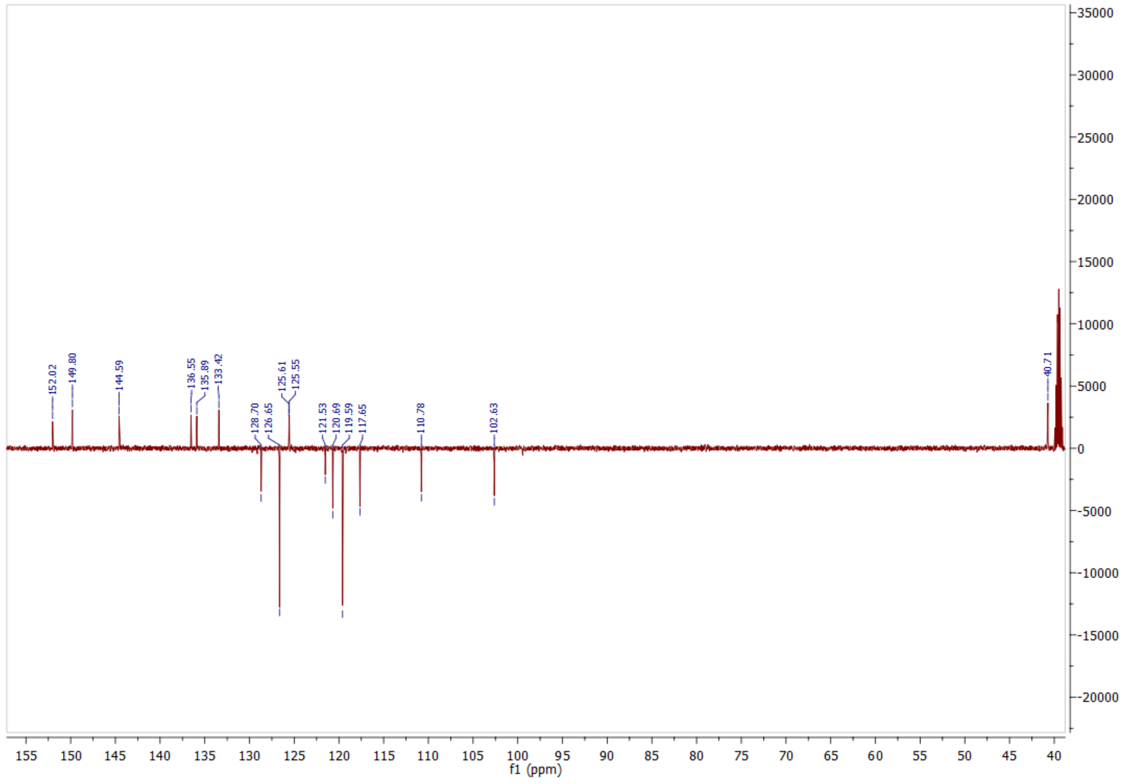

Figure S55: a) ^{1}H NMR and b) ^{13}C NMR of compd. **16**.

a)

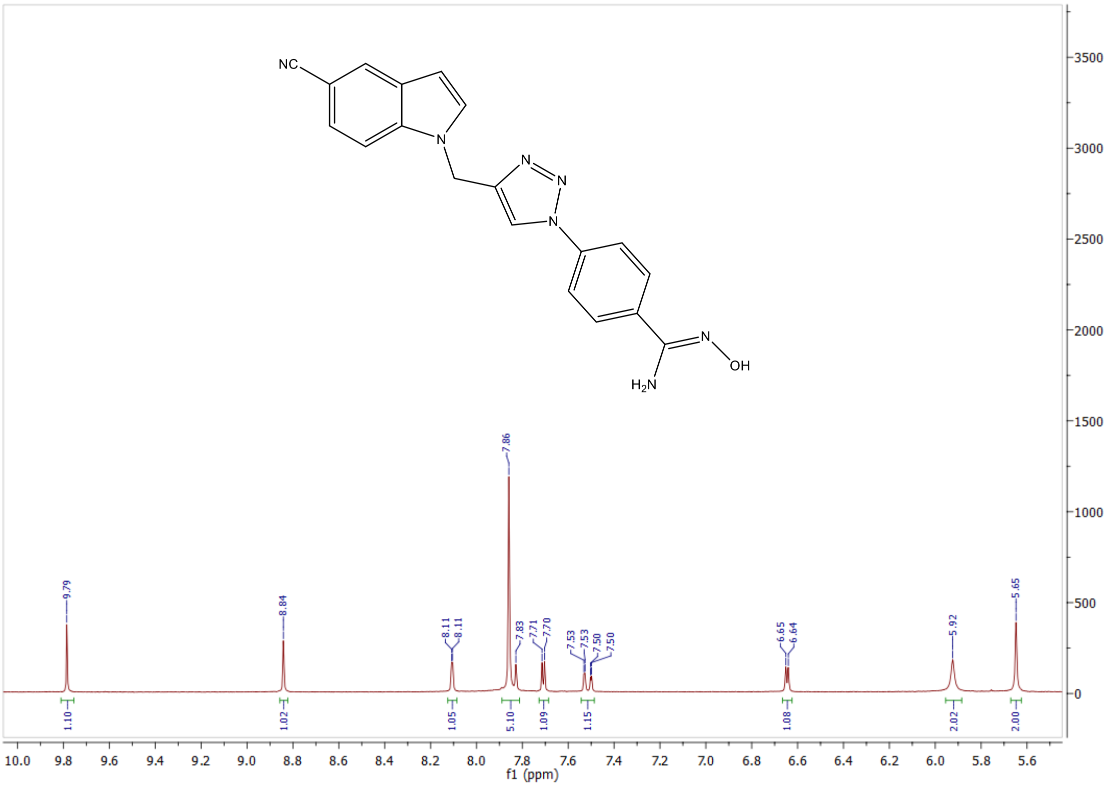

b)

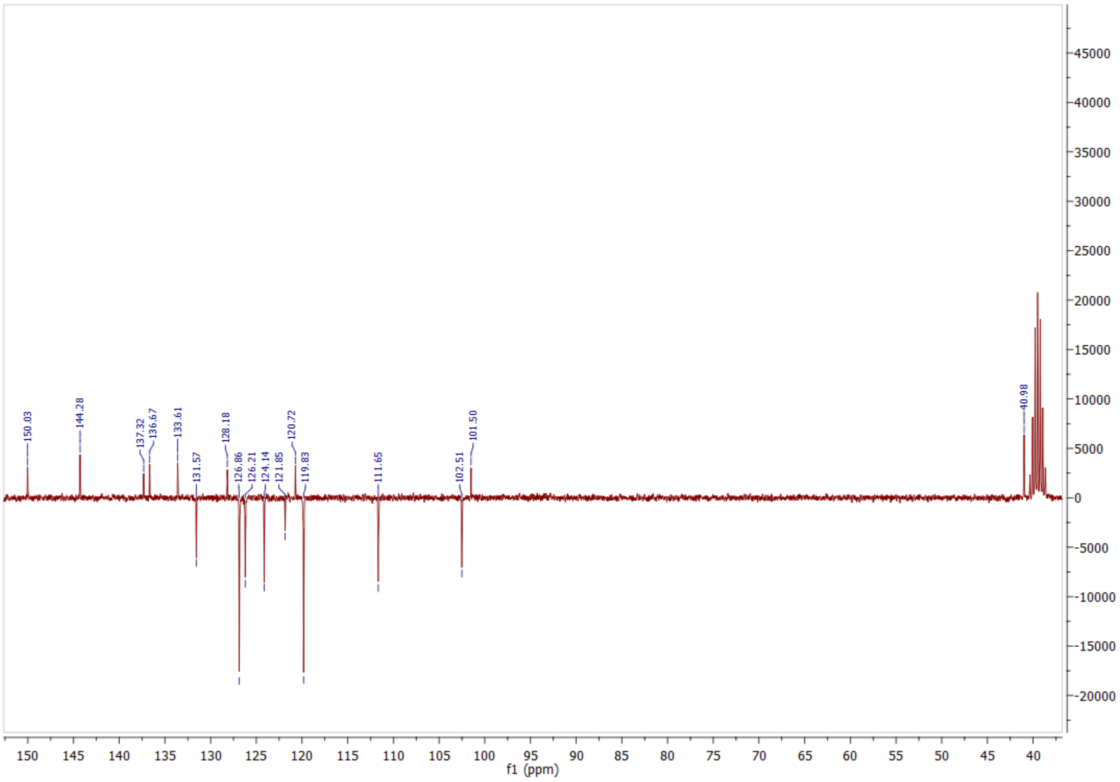

Figure S56: a) ^{1}H NMR and b) ^{13}C NMR of compd. **17**.

a)

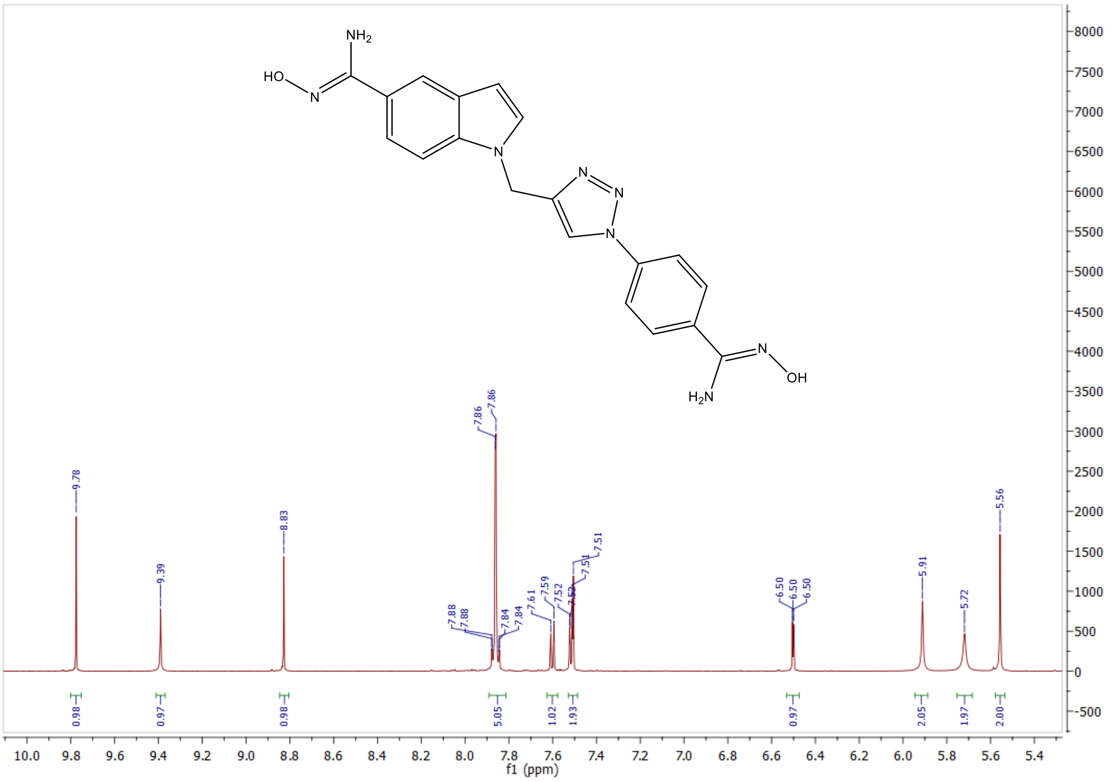

b)

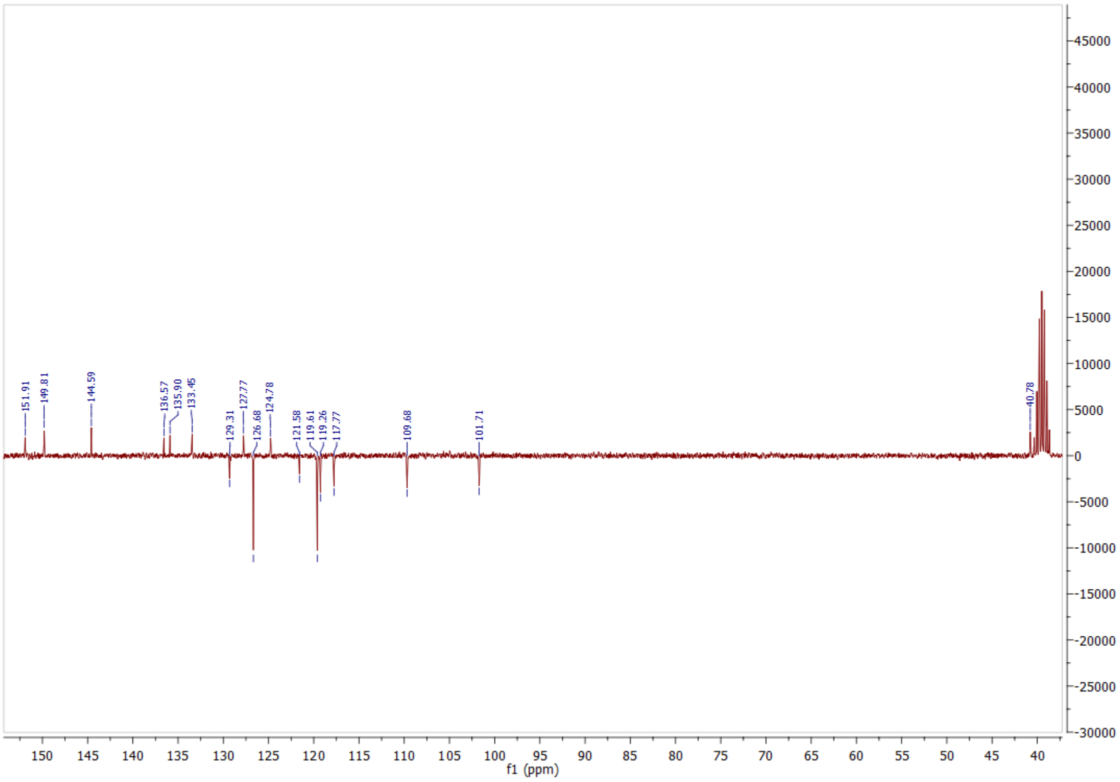

Figure S57: a) ^{1}H NMR and b) ^{13}C NMR of compd. **18**.

a)

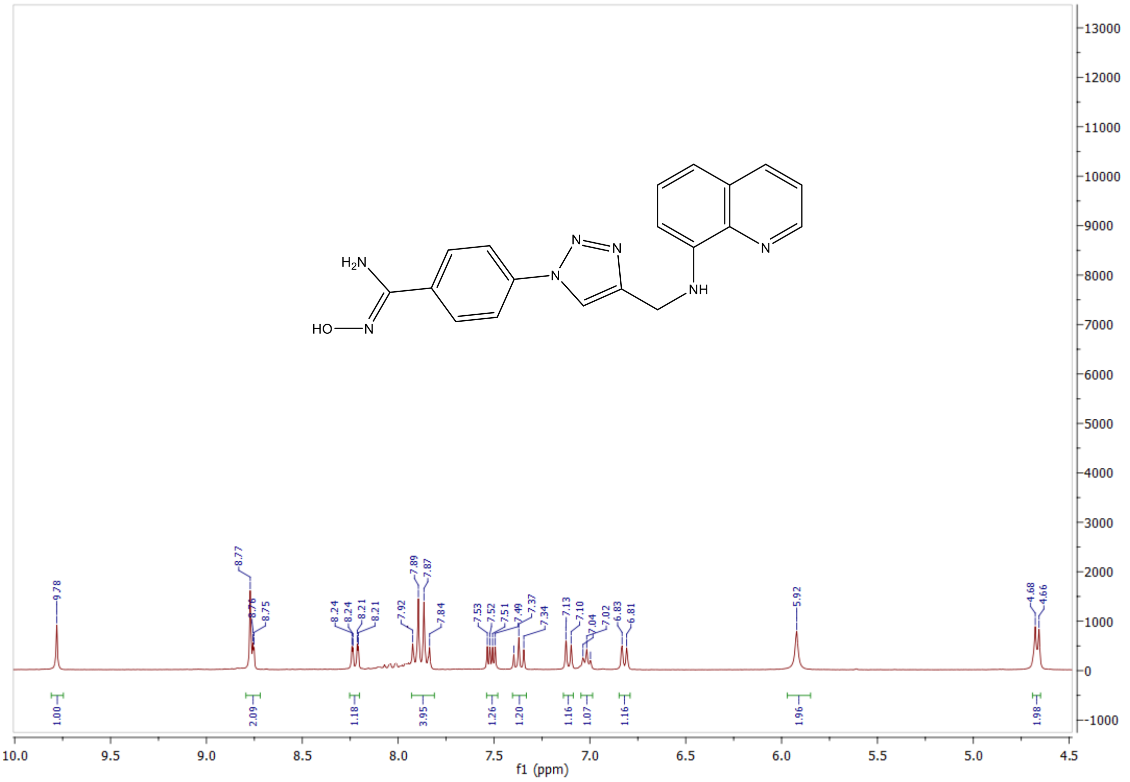

b)

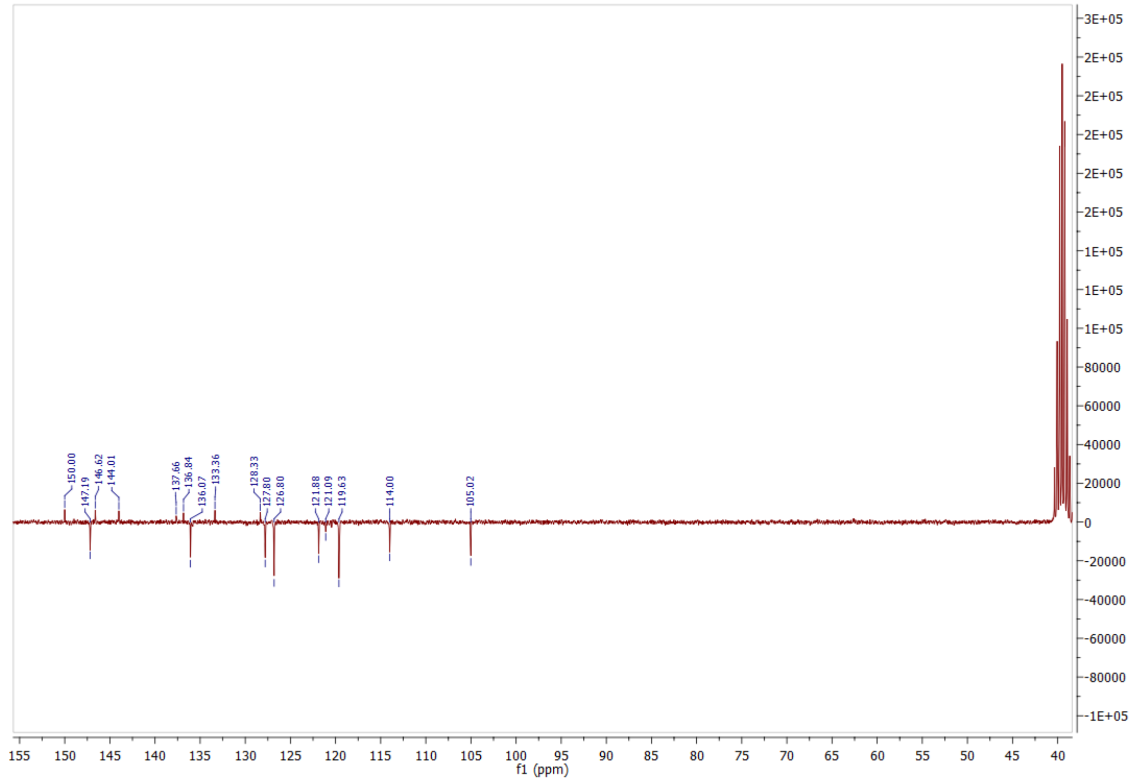

Figure S58: a) ^{1}H NMR and b) ^{13}C NMR of compd. **19**.

a)

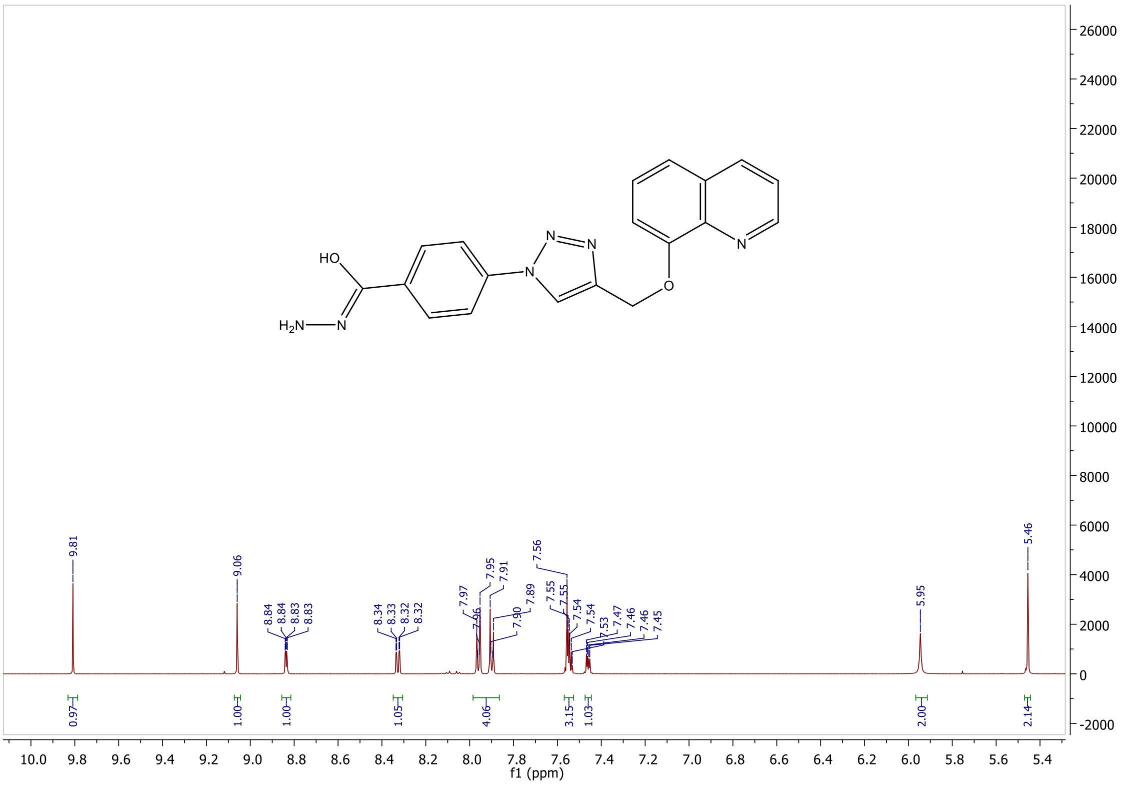

b)

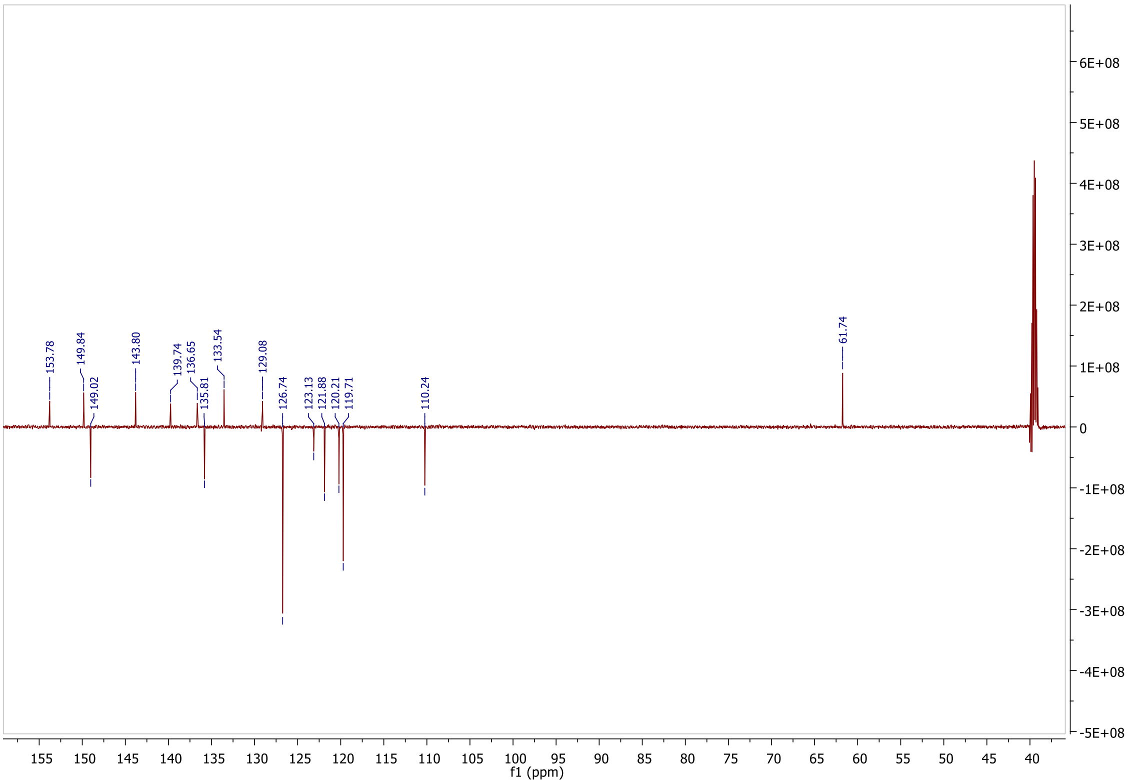

Figure S59: a) ^{1}H NMR and b) ^{13}C NMR of compd. **20**.

a)

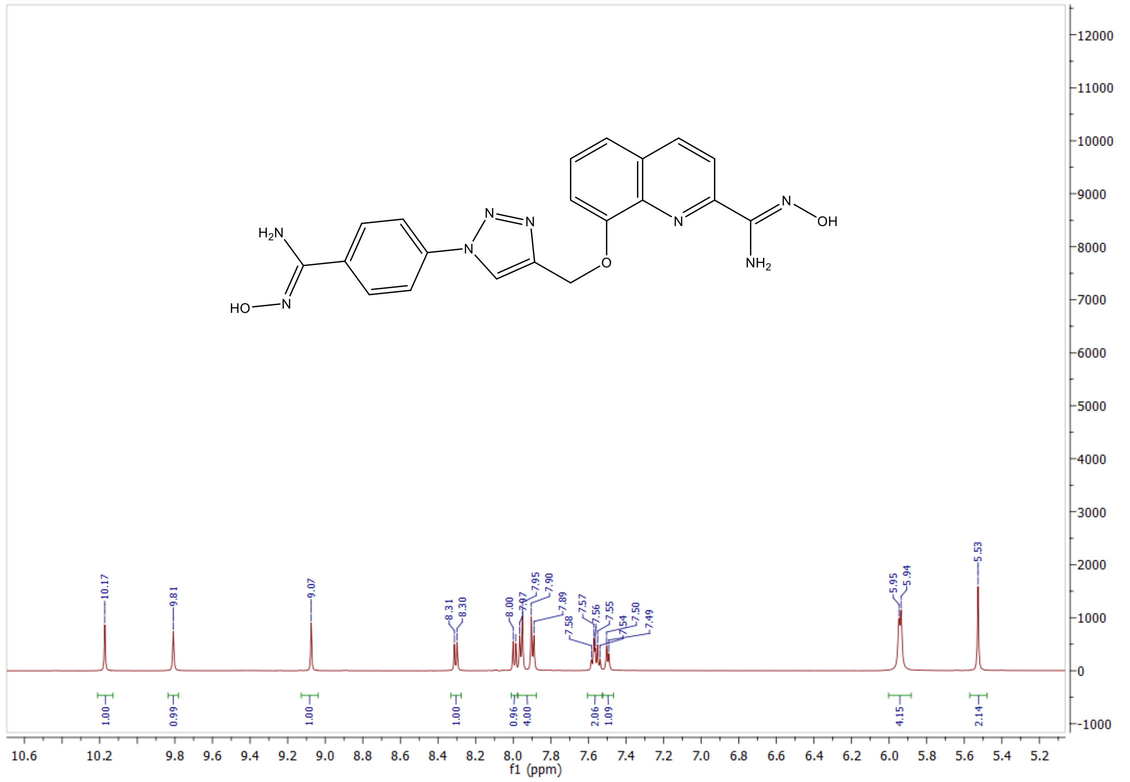

b)

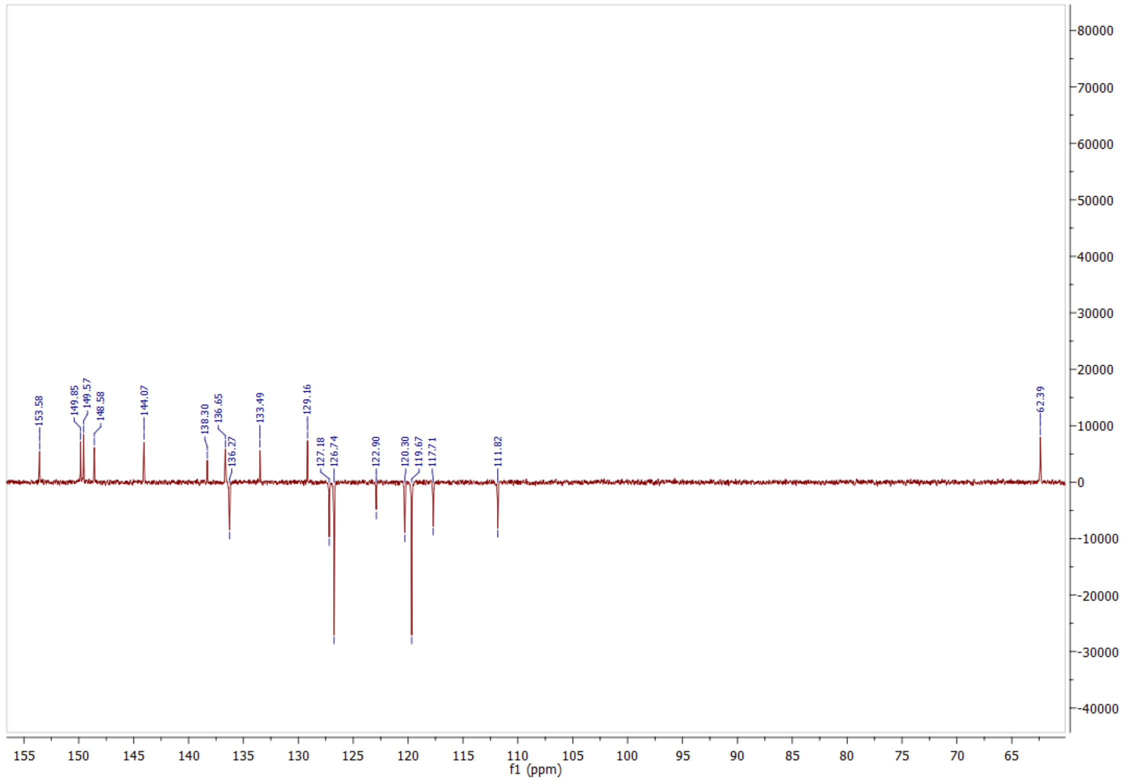

Figure S60: a) ^{1}H NMR and b) ^{13}C NMR of compd. **21**.

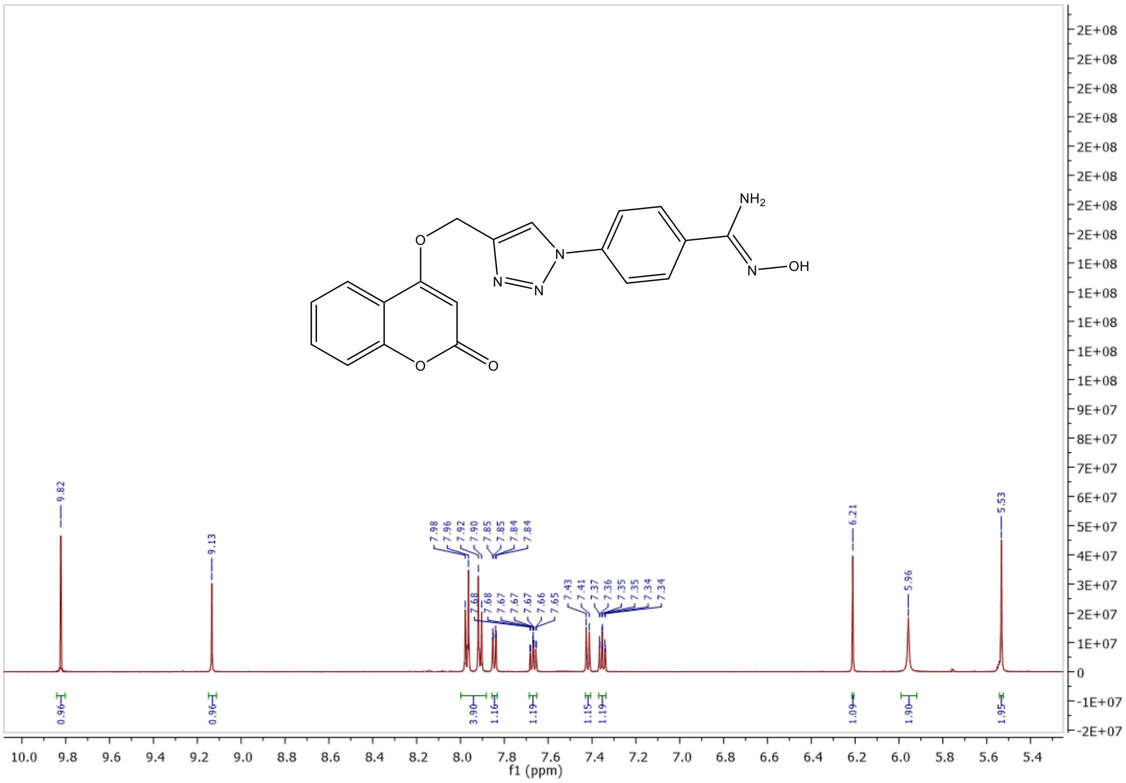

a)

b)

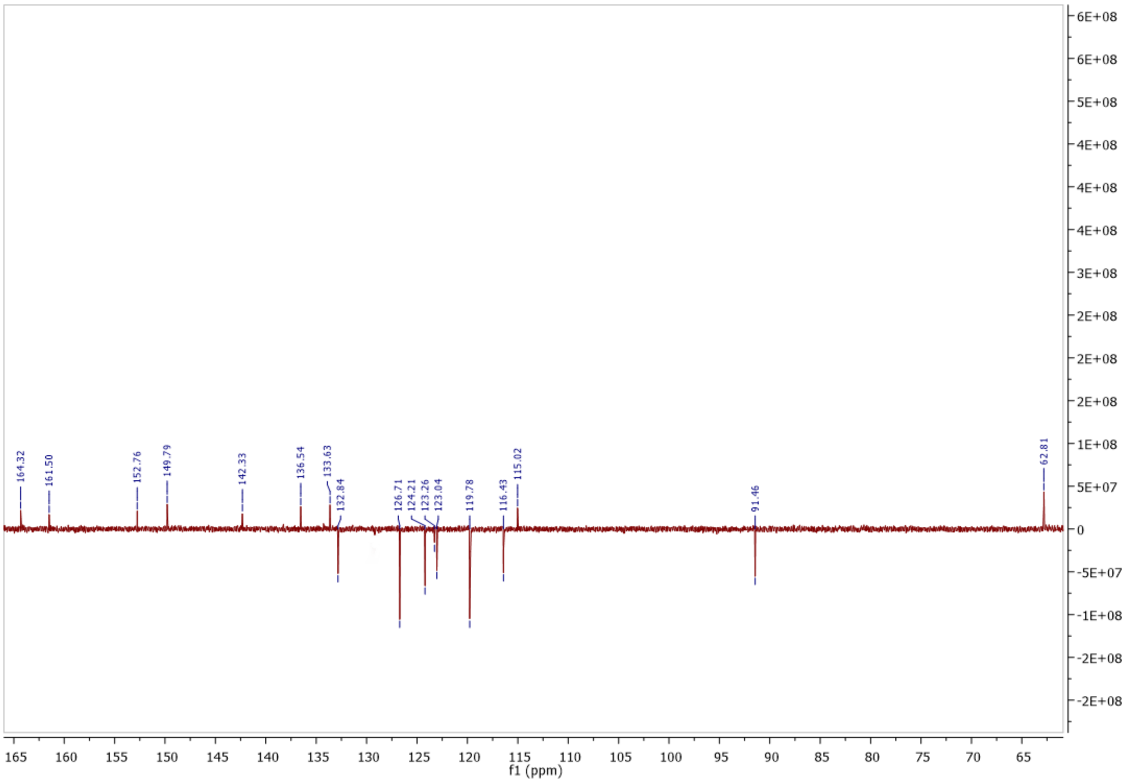

Figure S61: a) ^{1}H NMR and b) ^{13}C NMR of compd. **22**.

a)

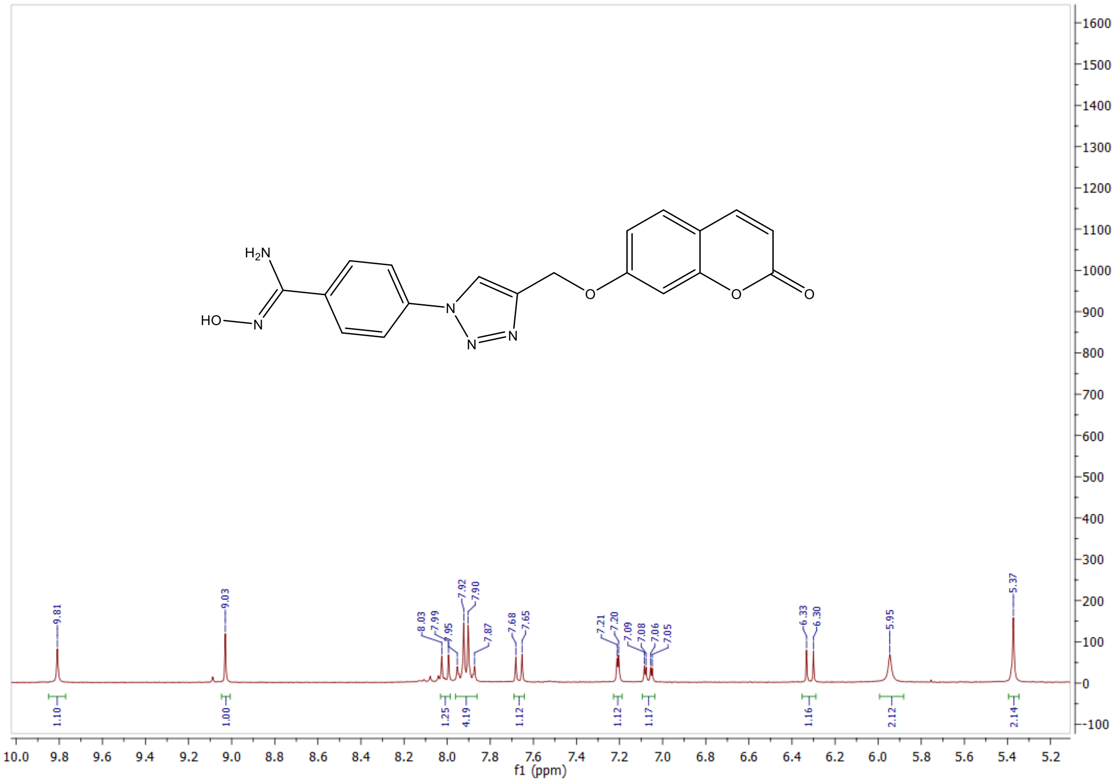

b)

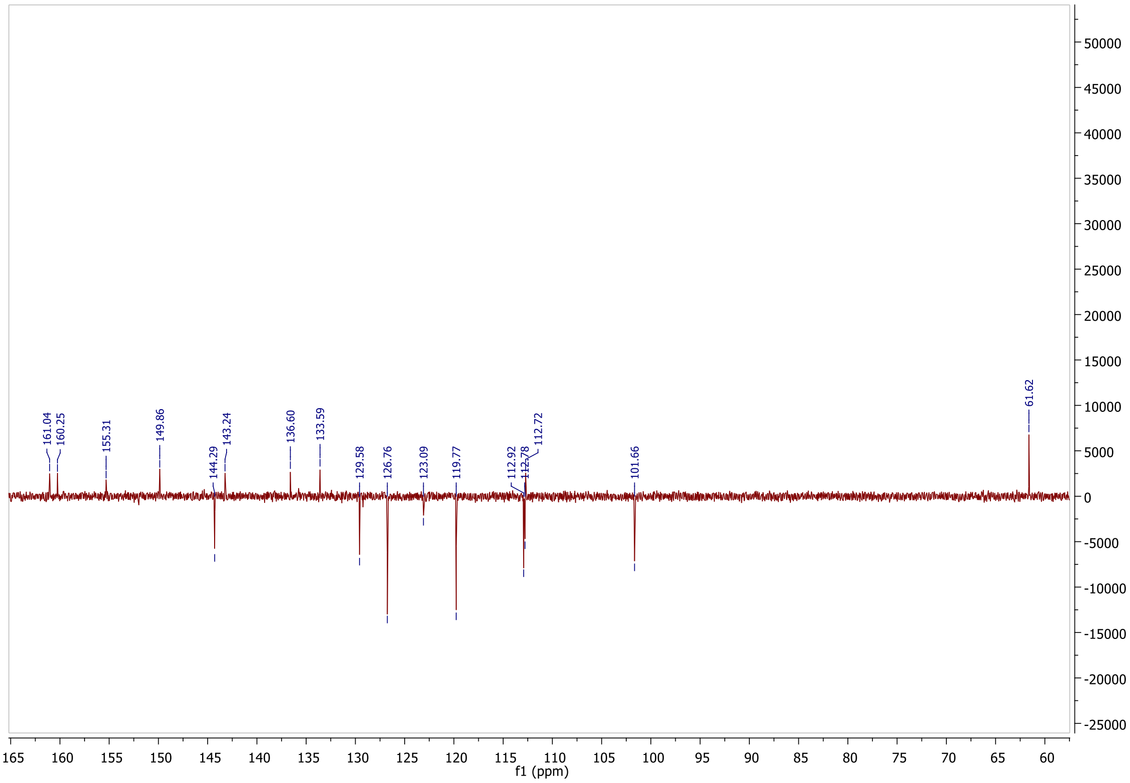

Supplement: Supplementary file 1 [file molecules-26-07060-s001.zip › molecules-1459358-supplementary.pdf]
